# Dihydropyrimidinone–isatin hybrids as potent inducers of cell-cycle arrest and caspase-dependent apoptosis in MV4-11 acute myeloid leukemia cells

**DOI:** 10.1039/d6ra03517e

**Published:** 2026-08-05

**Authors:** Preeti, Asif Raza, Jashandeep Kaur, Arun K. Sharma, Vipan Kumar

**Affiliations:** a Department of Chemistry, Guru Nanak Dev University Amritsar 143005 Punjab India vipan_org@yahoo.com; b Department of Molecular and Precision Medicine, Penn State Cancer Institute, The Pennsylvania State University College of Medicine Hershey PA 17033 USA asharma1@pennstatehealth.psu.edu

## Abstract

A novel series of 1*H*-1,2,3-triazole-linked dihydropyrimidinone (DHPM)–isatin hybrids was designed, synthesized and evaluated for antiproliferative activity against MV4-11 (acute myeloid leukemia), LN229 (glioblastoma) and SHSY-5Y (neuroblastoma). Among the synthesized derivatives, compound 7k, bearing a propyl-linked dibromoisatin-DHPM core, emerged as the most potent analogue, exhibiting an IC_50_ of 0.8 ± 0.1 µM in MV4-11 cells, significantly surpassing cisplatin (IC_50_ = 4.5 ± 0.2 µM), while showing low toxicity toward HaCaT cells, indicating good selectivity (SI = 37.12) as compared to cisplatin (SI = 7.62). Mechanistic studies demonstrated that 7k induced G0/G1 phase arrest in MV4-11 cells and elevated intracellular ROS levels, promoting mitochondrial dysfunction. The ROS induction strongly correlated with caspase activation and enhanced apoptotic cell death. Overall, the cytotoxic effects of 7k on MV4-11 cells are attributed to the coordinated induction of ROS-mediated oxidative stress, G0/G1 cell cycle arrest, and caspase-dependent apoptosis, highlighting its promising antiproliferative potential.

## Abstract 2

1*H*-1,2,3-triazole-linked DHPM–isatin hybrids exhibit potent antiproliferative activity against acute myeloid leukemia *via* ROS-induced oxidative stress, G2/M cell cycle arrest, and caspase-mediated apoptosis.

## Graphical abstract

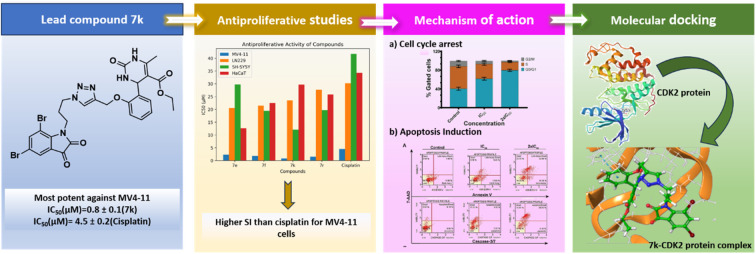

## 1 Introduction

Cancer continues to be a major global health challenge, with its incidence projected to rise by 70% over the next 20 years. Noncommunicable diseases (NCDs) account for 71% of worldwide deaths, while in India they contribute to 63% of fatalities, with cancer representing 9% of all deaths. Evidence from population- and hospital-based cancer registries indicates a steady increase in cancer cases, and the Indian Council of Medical Research (ICMR) estimates that cases will rise from 13.9 lakh in 2020 to 15.7 lakh by 2025.^1^ Although many anticancer drugs have been developed, their inability to distinguish between healthy and malignant cells often results in severe side effects. Additionally, emergence of resistance to existing chemotherapeutic agents is increasingly common. Therefore, there is an urgent need for new anticancer therapies that offer high specificity and improved effectiveness.^2^

Heterocyclic compounds play a crucial role in various scientific fields particularly in medicinal chemistry and biochemistry due to their structural diversity and adaptability. Several studies have reported hybrid molecules that have progressed through clinical or preclinical evaluation, highlighting their potential as safer and more effective therapeutics for a broad range of diseases. Notably, more than 75% of FDA-approved drugs incorporate heterocyclic frameworks, with nitrogen-containing heterocycles being the most prevalent.^3^

Isatin (indole-2,3-dione), naturally present in mammalian tissues and fluids, is a highly versatile scaffold widely used in medicinal chemistry. Its structure allows modifications at multiple positions (N-, C2-, C3-, and aromatic rings), enabling extensive exploration of its therapeutic potential. Owing to its ability to function as both an electrophile and a nucleophile, isatin serves as an important building block in organic synthesis.^3^ It also exhibits strong inhibitory activity against several key enzymes and receptors including HDAC, carbonic anhydrase, tyrosine kinase, and tubulin, thereby inducing apoptosis and modulating apoptosis-related genes. Its derivatives display broad biological activities, such as antibacterial, anticancer, antiviral, and analgesic effects. Several isatin-based agents, including indirubin, sunitinib, semaxanib, nintedanib, and hesperidin, have shown promise in treating diverse and drug-resistant cancers, underscoring their value in anticancer drug development.^2,4^ Additionally, several naturally occurring polybrominated indole- and isatin-based metabolites such as Rivularin A, Aetokthonotoxin (AETX), and the Kikai-island indole alkaloids have been reported to exhibit diverse biological activities, highlighting the pharmacological relevance of brominated heterocyclic scaffolds.^5–7^

Dihydropyrimidinone (DHPM) and 1,2,3-triazole moieties represent important pharmacophores in medicinal chemistry, with many derivatives demonstrating antitumor, antiviral, antifungal, and anti-inflammatory activities. The discovery of monastrol, an antimitotic agent that selectively inhibits the Eg5 kinesin and its analogues stimulated extensive research into DHPM-based compounds.^8,9^ Similarly, the 1,2,3-triazole ring found in drugs such as the β-lactam antibiotic cefatrizine is valued for its exceptional stability and resistance to hydrolysis, oxidation, and reduction. Its ability to serve as both a hydrogen-bond donor and acceptor, participate in π–π stacking interactions, and act as a bioisostere for functional groups like amides and esters further enhances its utility in drug design.^8^

**Fig. 1:**
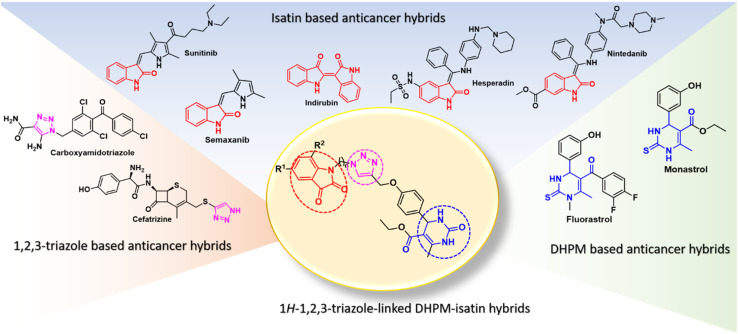
Design rationale for synthesis of 1*H*-1,2,3-triazole-linked DHPM–isatin hybrids.

Given the strong pharmacological significance of isatin, DHPM, and triazole scaffolds, we designed a new series of 1*H*-1,2,3-triazole-linked DHPM–isatin hybrids (Fig. 1) as potential antiproliferative agents using a molecular hybridization strategy. This approach merges two distinct pharmacophoric units into a single molecule, aiming to improve target specificity, enhance therapeutic efficacy, and may help mitigate or delay drug resistance.^3,10^

In our earlier studies,^11–13^ we successfully designed and synthesized a series of isatin-based molecular hybrids by incorporating diverse pharmacophores, including triazolopyrimidine, triclosan and bisnaphthalimide scaffolds through molecular hybridization approach. These compounds were subsequently evaluated against multiple cancer cell lines and demonstrated notable antiproliferative activity, highlighting the therapeutic relevance of the isatin core and validating our design strategy. In this study, a series of novel 1*H*-1,2,3-triazole-tethered DHPM–isatin hybrids were designed, synthesized, and evaluated for their anticancer potential. Among these, the 5,7 dibromo substituted analogue 7k emerged as the most promising lead, exhibiting submicromolar potency and a high selectivity index against MV4-11 acute myeloid leukemia cells relative to cisplatin while showing limited toxicity toward non malignant HaCaT keratinocytes. Mechanistic investigations demonstrate that 7k induces G0/G1 cell cycle arrest, elevates intracellular ROS levels, and causes oxidative DNA damage, culminating in caspase dependent apoptotic cell death in MV4-11 cells. These findings highlight DHPM–isatin hybrids, and 7k in particular, as attractive leads for further development of targeted therapies for acute myeloid leukemia.

## 2 Results and discussion

### 2.1 Synthetic chemistry

**Scheme 1:**
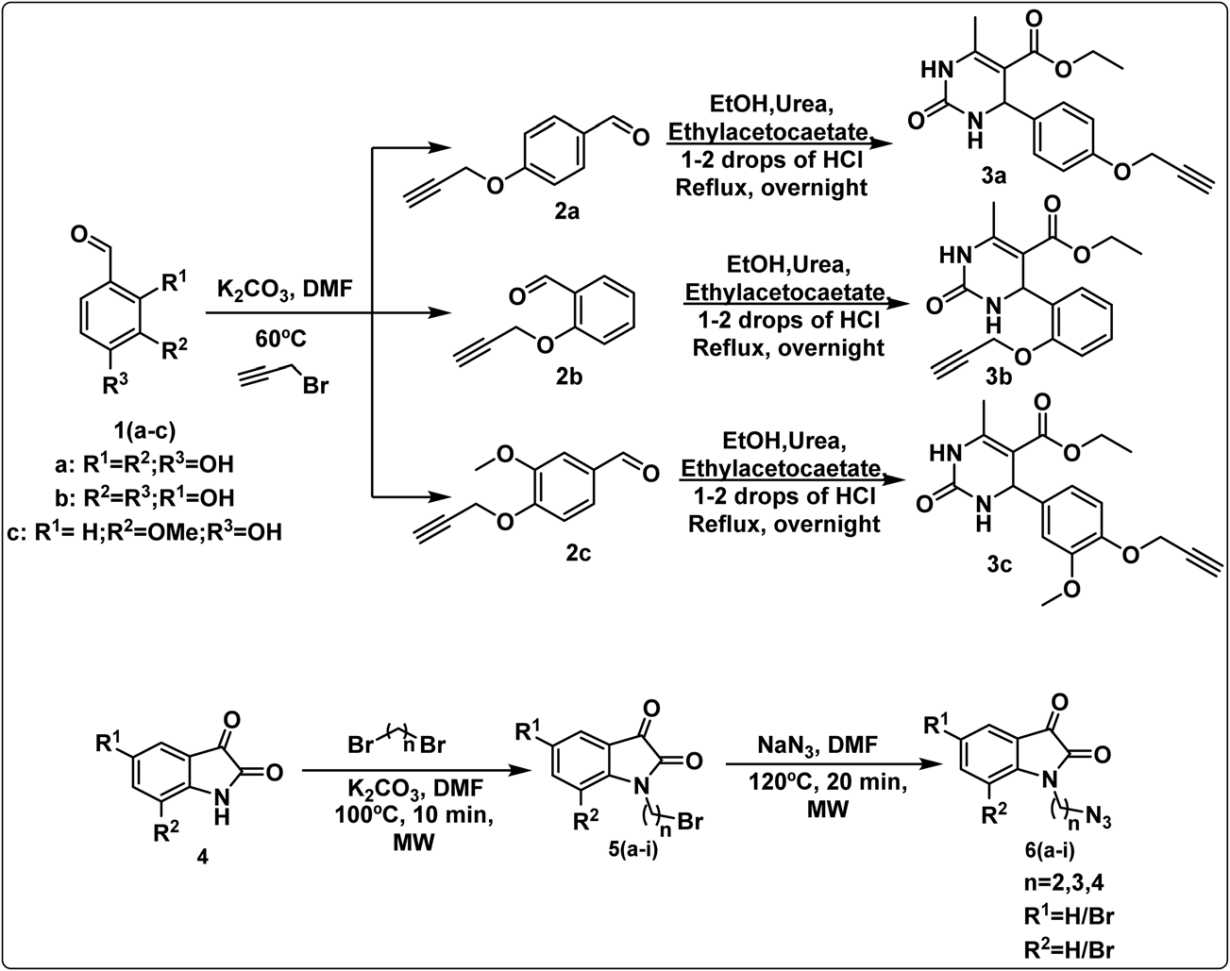
Synthesis of precursors' 3(a–c) and 6(a–i).

The synthetic methodology for the synthesis of desired dihydropyrimidines involved an initial synthesis of the precursor, *O*-propargylated substituted aldehydes, obtained by reacting substituted aldehydes with propargyl bromide in DMF at 100 °C, using K_2_CO_3_ as a base. Subsequent refluxing of the propargylated aldehydes with urea and ethylacetoacetate in ethanol, along with 1–2 drops of HCl overnight resulted in the formation of the desired Biginelli adducts. The structures of the synthesized adducts 3(a–c) were assigned based on spectral data and analytical evidence. The synthesis of *N*-alkyl azido isatin derivatives, the second precursor was synthesized *via* initial base-promoted *N*-alkylation of isatin with dibromoethane in DMF at 60 °C for 2 h to yield the corresponding *N*-alkyl bromo isatin, followed by subsequent reaction with sodium azide in DMF at 60 °C for 3 h resulting in *N*-alkyl azido-isatin derivatives 6(a–i) as shown in Scheme 1.

**Scheme 2:**
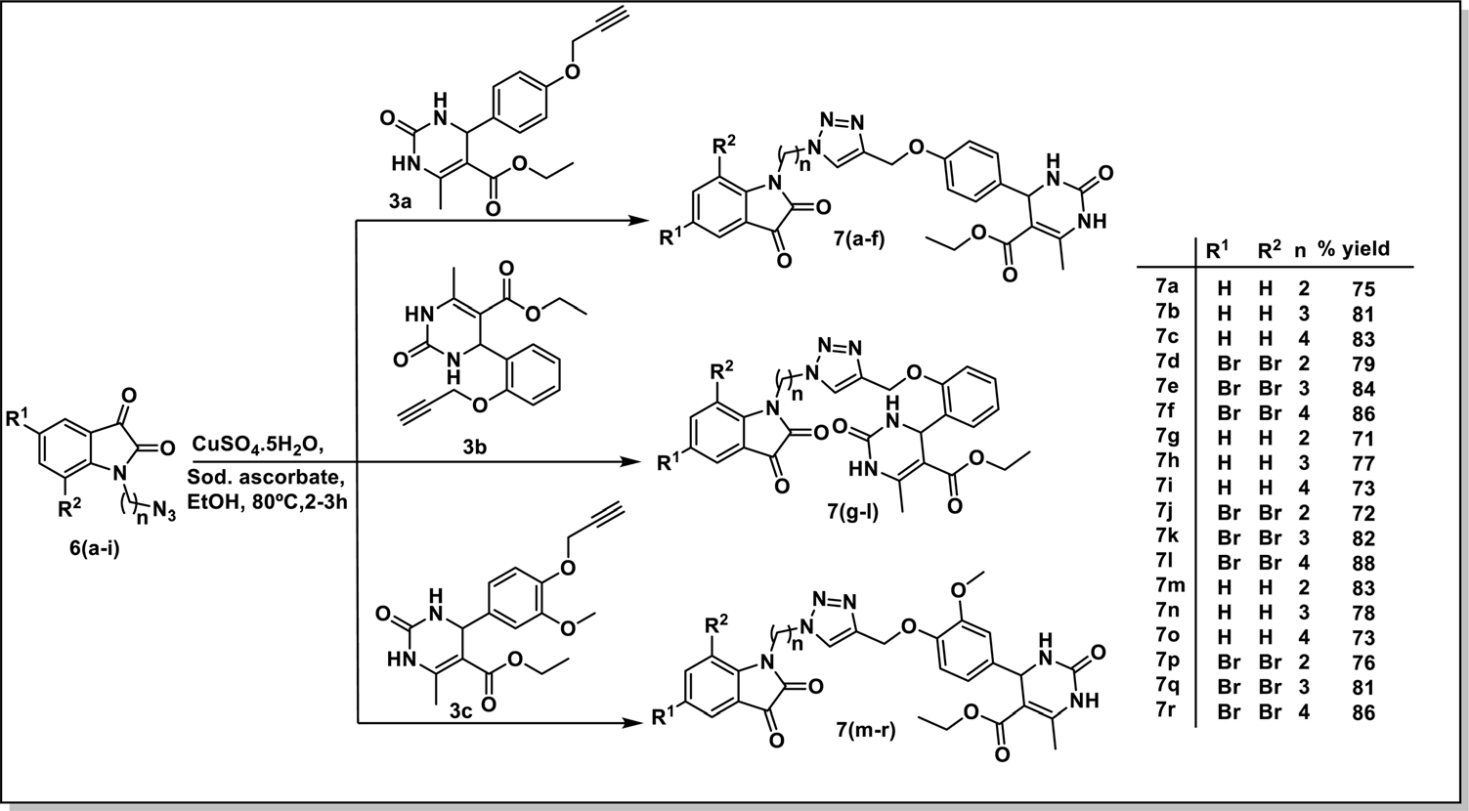
Synthesis of 1*H*-1,2,3-triazole-linked DHPM–isatin hybrids 7(a–r).

The desired hybrids were obtained *via* copper-promoted azide–alkyne cycloaddition (CuAAC) between the alkyne functionalized DHPMs 3(a–c) and the *N*-alkyl azido isatin derivatives 6(a–i), as outlined in Scheme 2. The reaction was performed at 80 °C for 3–4 hours in ethanol using CuSO_4_·5H_2_O as the catalyst and sodium ascorbate as a mild reducing agent.

The structures of the synthesized scaffolds were assigned using standard spectroscopic and analytical techniques. For example, the salient features of ^1^H NMR of 7g showed characteristic singlets at *δ* 5.19, 5.55 and 2.33 corresponding to O–C**H**_2_–CH, CH_2_–C**H**–NH and NH–C–C**H**3. Its ^13^C NMR spectrum exhibited absorptions at *δ* 183.27 and 165.81 corresponding to keto carbonyl (C=O) group of the isatin, and ester (C=O) attached to DHPM respectively, thus, validating the assigned structure. HRMS calcd for C_27_H_26_N_6_O_6_ [M + H]^+^ 531.1992 and found 531.1996.

### 2.2 *In vitro* antiproliferative evaluation

Acute myeloid leukemia (AML) and glioblastoma (GBM) are aggressive malignancies arising from immature myeloid and glial cells, respectively. AML is among the most common hematologic cancers in adults, while GBM represents the most prevalent primary brain tumor.^14,15^ Despite differences in origin, both diseases share characteristics linked to poor prognosis. Survival outcomes remain poor; AML exhibits a 5 year survival rate of about 28% in patients under 40 and under 10% in older adults, while standard therapy for GBM yields a 2 year survival rate of only 26.5%.^16–18^

Neuroblastoma (NB), in contrast, is the most common extra cranial solid tumor of childhood and arises from embryonal neuroendocrine cells derived from the neural crest. It can arise anywhere along the sympathetic nervous system, most frequently in the adrenal glands. NB presents with highly variable symptoms, ranging from mild abdominal distension to severe illness due to metastatic spread. Low-risk patients experience excellent cure rates, whereas high-risk groups show minimal therapeutic progress. This disparity underscores the urgent need for novel targeted therapies to improve survival in high-risk NB.^19^

**Fig. 2:**
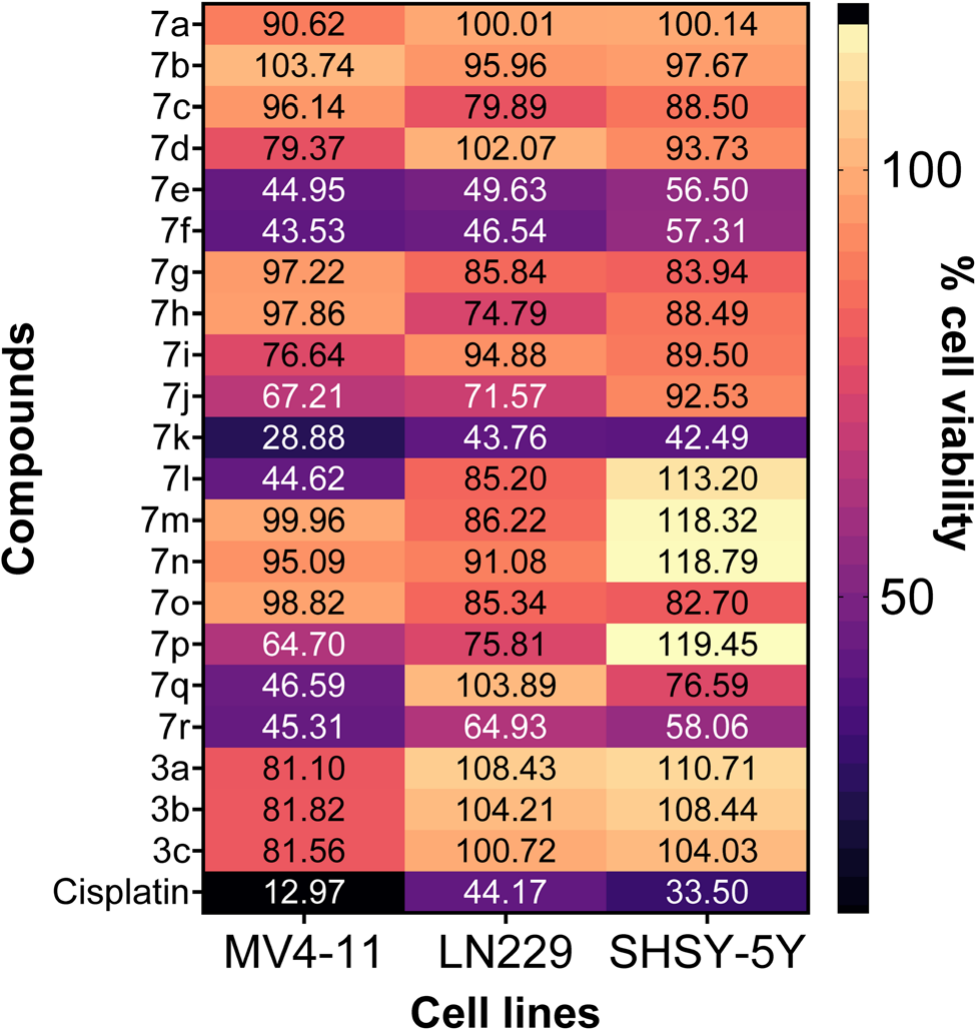
Cell growth percentage of MV4-11, LN229, and SHSY-5Y cells treated with 25 µM of the reported compounds after 48 h of treatment.

All the synthesized hybrids were screened for their antiproliferative effects against three human cancer cell lines, MV4-11 (acute myeloid leukemia), LN229 (glioblastoma), and SHSY-5Y (neuroblastoma), along with the non-malignant human keratinocyte line HaCaT, using the MTS cell viability assay. Cells were exposed to 25 µM of each compound for 48 hours (Fig. 2). The results, expressed as the percentage of viable cells, represent the mean ± SD of at least three independent experiments performed in triplicate. Derivatives showing more than 50% growth inhibition in all three cancer cell lines were further evaluated in dose–response assays (1–50 µM, 48 h). The half-maximal inhibitory concentration (IC_50_) values obtained from these experiments are summarized in Table 1.

**Table 1:** IC_50_ values (µM) of the selected novel synthetized compounds against MV4-11, LN229, SHSY-5Y, and HaCaT cell lines

Compounds	MV4-11	LN229	SHSY-5Y	HaCaT
7e	2.3 ± 0.6	20.5 ± 1.3	29.8 ± 2.9	12.6 ± 1.6
7f	1.8 ± 0.3	21.5 ± 1.7	19.4 ± 1.5	22.5 ± 1.7
7k	0.8 ± 0.1	23.5 ± 2.2	12.1 ± 1.2	29.7 ± 3.1
7r	1.5 ± 0.2	27.7 ± 3.0	19.7 ± 1.0	25.8 ± 2.8
TMZ	>100	>100	>100	>100
Cisplatin	4.5 ± 0.2	30.2 ± 3.8	41.7 ± 2.3	34.3 ± 4.2

Cell viability studies revealed that the synthesized compounds displayed markedly higher activity against MV4-11 cells compared with LN229 and SHSY-5Y cells. Systematic analysis of substituent effects showed that bromo substitution at the C-5 and C-7 positions of the isatin core consistently enhanced anticancer activity relative to un-substituted analogs. In particular, the dibromo-substituted derivatives 7e, 7f, 7k, and 7r demonstrated strong potency against MV4-11 cells, even surpassing the standard drug cisplatin (Table 1). Furthermore, compounds with propyl and butyl linkers exhibited improved activity compared with their ethyl counterparts. Among all synthesized hybrids, compound 7k (*n* = 3) was identified as the most potent, with an IC_50_ of 0.8 ± 0.1 µM in MV4-11 cells, significantly outperforming cisplatin (IC_50_ = 4.5 ± 0.2 µM). These observations highlight the importance of halogen substitution patterns and spacer length in modulating the antiproliferative activity of this class of triazole-tethered DHPM isatin hybrids (Table 1).

To determine whether the synthesized compounds were cytotoxic or antiproliferative, they were also tested on the non-cancerous human epidermal keratinocyte line HaCaT. The most active compound, 7k, selectively targeted cancer cells with acceptable cytotoxicity toward HaCaT cells (IC_50_ = 29.7 ± 3.1 µM). Compound 7e, 7f, 7k and 7r showed higher selectivity against MV4-11 cell lines as compared to LN229 and SHSY-5Y cell lines as listed in Table 2. Among these, 7k exhibited highest SI of 37.12 surpassing the standard cisplatin (SI = 7.62) highlighting its greater selectivity towards the MV4-11 (acute myeloid leukemia) cancer cells.

**Table 2:** The selectivity index (SI) values of the best identified compounds against MV4-11, LN229 and SHSY-5Y cell lines

Compounds	Selectivity index (SI)		
	MV4-11	LN229	SHSY-5Y
7e	5.47	0.61	0.42
7f	12.5	1.04	1.1
7k	37.12	1.26	2.45
7r	17.2	0.93	1.30
Cisplatin	7.62	1.13	0.82

**Fig. 3:**
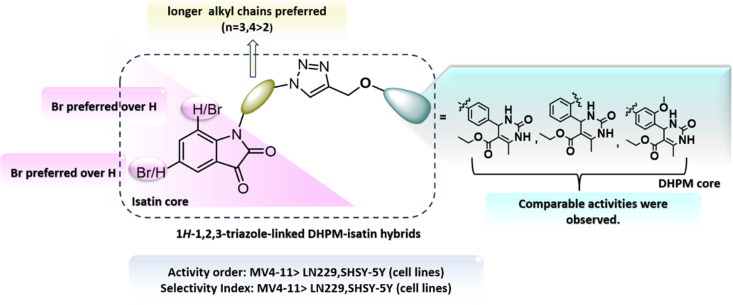
Generalized graphical representation of SAR.

The structure–activity relationship (SAR) analysis (Fig. 3) based on the cell viability and IC_50_ values presented in Fig. 2 and Table 1, respectively revealed distinct trends influenced by halogen substitution and alkyl chain length.

1. All synthesized hybrids showed superior potency and selectivity toward MV4-11 cells compared to LN229 and SHSY-5Y cells.

2. The dibromo-substitution at the C-5 and C-7 positions of the isatin significantly enhanced activity outperforming unsubstituted analogs as well as the corresponding parent molecules.

3. For both H- and Br-substituted analogs, the incorporation of longer alkyl chain linkers (*n* = 3, 4) resulted in improved antiproliferative activities.

4. In contrast, modifications on the DHPM core using different aldehydes (4-OH benzaldehyde, 2-OH benzaldehyde, and vanillin) produced similar activity profiles, suggesting that DHPM-ring substitutions exert minimal influence compared with the effects on isatin and linker optimization.

Notably, compound 7k, exhibited the most pronounced cytotoxic effect across all tested cancer lines while maintaining relatively low toxicity toward HaCaT cells. This selectivity suggests that 7k may exert its antitumor activity through mechanisms involving apoptosis signaling.

### 2.3 Apoptosis assay

Apoptosis is a vital mechanism for maintaining tissue homeostasis by removing unnecessary or damaged cells. It is primarily regulated through two major signaling pathways: the intrinsic (mitochondrial) and extrinsic (death receptor) pathways. These pathways rely on initiator caspases, such as caspase-8 and -9, and executioner caspases, including caspase-3, -6, and -7, to drive the apoptotic cascade. In the extrinsic pathway, ligands like TNF trigger the formation of the death-inducing signalling complex (DISC), leading to caspase-8 activation and subsequent downstream events.^20,21^ Once activated, caspases induce DNA fragmentation and systematic breakdown of cellular structures. Disruption of apoptotic regulation contributes to the development of diseases such as cancer, autoimmune conditions, neurodegenerative disorders, and cardiovascular diseases.^22–24^

**Fig. 4:**
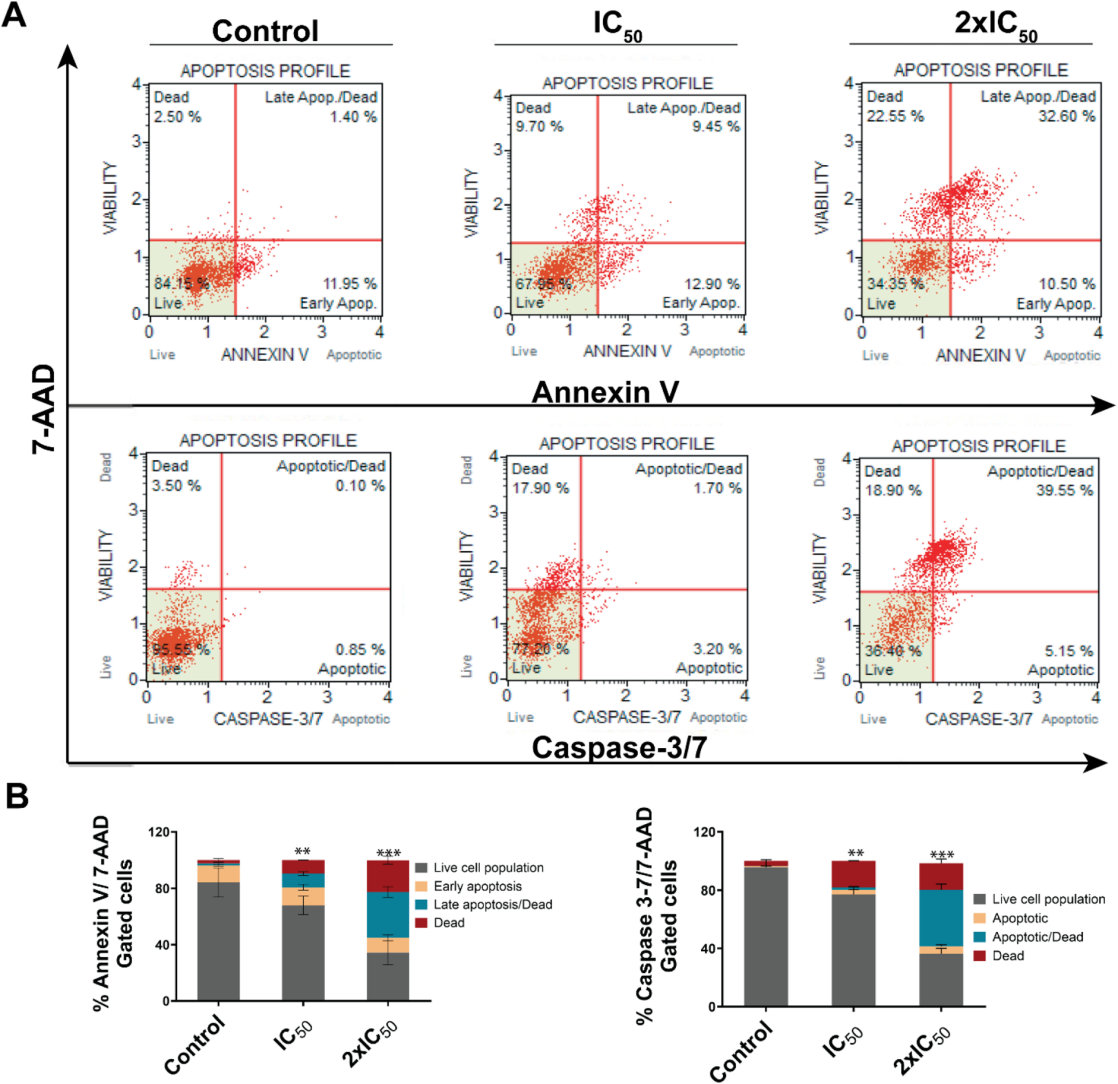
Compound 7k induced apoptotic cell death in MV4-11 cells. Cells were treated with IC_50_ and twice the IC_50_ values of compound 7k for 48 h and examined on a Muse™ automated cell analyzer with the Annexin V & Dead Cell, and caspase 3/7 apoptosis assays (A). Quantification of the cell population with the Annexin V & Dead Cell, and caspase 3/7 (B) assays. Data is presented as the mean ± SD of at least three independent experiments.

Flow cytometry offers a fast, reliable, and reproducible method for assessing and quantifying cell-cycle parameters in surviving cell populations. To confirm whether the cytotoxic effect of 7k was associated with apoptosis, MV4-11 leukemia cells were treated with 7k at its IC_50_ and 2 × IC_50_ concentrations for 48 hours, and analyzed using Annexin V & Dead Cell and caspase 3/7 assay kits on a Muse™ automated cell analyzer (Fig. 4). In the Annexin V & Dead Cell assay, treatment with 7k resulted in a marked decrease in viable cell populations and a corresponding rise in apoptotic cells. Specifically, cells exposed to 7k at the IC_50_ concentration showed approximately 67.95% live cells and 22.35% apoptotic cells, whereas exposure to 2 × IC_50_ reduced viability to about 34.35%, with 43.10% of cells undergoing apoptosis. These results demonstrate a strong, concentration-dependent induction of apoptotic cell death.

Similarly, the caspase 3/7 assay revealed activation of the executioner phase of apoptosis. Following 48 h treatment with 7k, 77.2% of cells remained viable at the IC_50_ dose, compared to only 36.40% at 2 × IC_50_, accompanied by significant caspase 3/7 activation in the apoptotic population. The parallel trend observed in both assays underscores that 7k induces caspase-dependent programmed cell death rather than necrosis. Together, these findings suggest that 7k exerts its cytotoxicity through activation of the intrinsic apoptotic pathway.

### 2.4 Cell cycle dysregulation induced by 7k

**Fig. 5:**
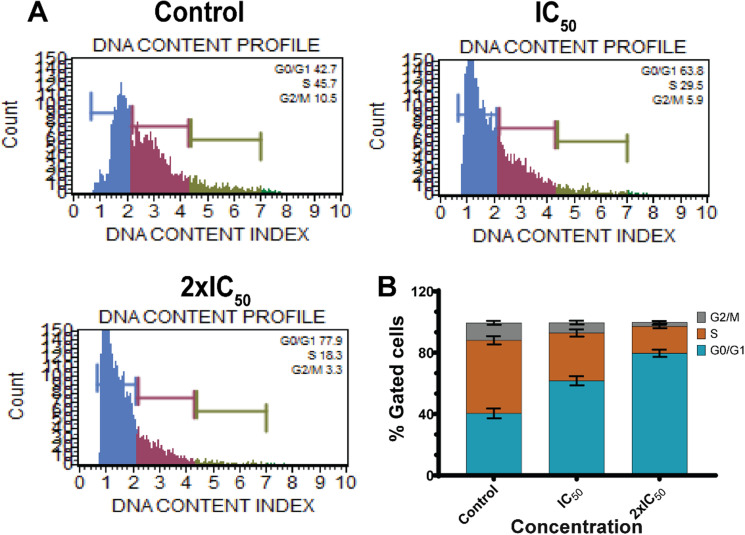
Compound 7k induced cell cycle arrest in MV4-11 cells. MV4-11 cells were treated with IC_50_ and twice the IC_50_ concentrations of compound 7k for 48 h and analyzed using the Muse™ Cell Cycle Assay on a Muse™ automated cell analyzer (A). Representative histograms showing distribution of cells in G0/G1, S, and G2/M phases are presented. Quantitative analysis of cell cycle distribution following treatment is shown (B). Data are expressed as mean ± SD of at least three independent experiments performed in triplicate.

To further elucidate the mechanism underlying the antiproliferative effect of compound 7k, cell cycle distribution was evaluated in MV4-11 cells following 48 h exposure to IC_50_ and 2 × IC_50_ concentrations. Flow cytometric analysis revealed a distinct alteration in cell cycle progression compared with vehicle-treated controls (Fig. 5). Treatment with 7k resulted in a significant accumulation of cells in the G0/G1 phase, accompanied by a concomitant reduction in the S population. The effect was concentration-dependent, with 2 × IC_50_ treatment producing a more pronounced G0/G1 arrest. A modest decrease in G2/M-phase population was also observed, suggesting impaired DNA synthesis and cell cycle progression.

These findings indicate that compound 7k disrupts normal cell cycle regulation, likely interfering with mitotic progression. The observed G0/G1 arrest is consistent with the enhanced cytotoxicity seen in MV4-11 cells and aligns with the strong antiproliferative activity demonstrated in the MTS assay. Cell cycle arrest at the G0/G1 checkpoint is frequently associated with DNA damage response and subsequent activation of apoptotic pathways. Therefore, the G0/G1 accumulation induced by 7k may act upstream of the caspase-dependent apoptosis observed in Annexin V and caspase 3/7 assays, further supporting its mechanism of action as a potent antiproliferative agent in acute myeloid leukemia cells.

### 2.5 ROS generation as an upstream trigger of apoptotic signaling

**Fig. 6:**
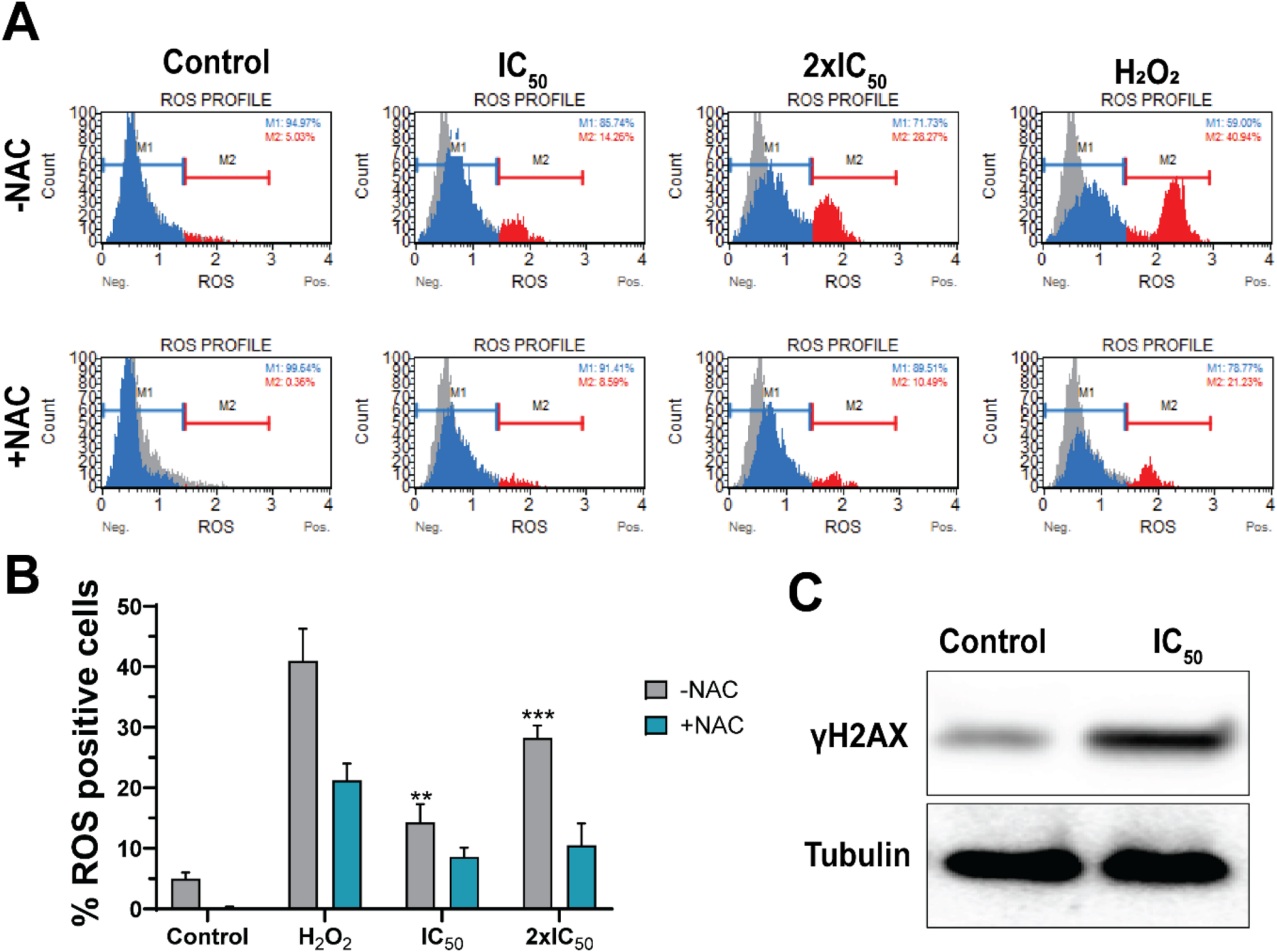
Compound 7k increased intracellular ROS levels in MV4-11 cells. MV4-11 cells were treated with IC_50_ and twice the IC_50_ concentrations of compound 7k for 48 h and analyzed using the Muse™ Oxidative Stress Assay kit on a Muse™ automated cell analyzer (A). Representative dot plots demonstrating ROS-positive and ROS-negative cell populations are shown in presence and absence of NAC. Quantification of intracellular ROS generation following treatment with 7k is presented (B). (C) Western blot analysis of γH2AX as a marker of DNA double-strand breaks in MV4-11 cells treated with vehicle (control) or compound 7k at its IC_50_ concentration for 48 h. α-Tubulin was used as a loading control. Data are expressed as mean ± SD of at least three independent experiments performed in triplicate.

Because oxidative stress is a well-established mediator of apoptosis in cancer cells, we next examined whether 7k induced cytotoxicity was associated with intracellular ROS accumulation. MV4-11 cells treated with 7k for 48 h showed a pronounced increase in ROS-positive populations compared with vehicle-treated controls (Fig. 6A). At the IC_50_ concentration, 7k produced a clear elevation in ROS levels, which was further enhanced at 2 × IC_50_, demonstrating a concentration-dependent effect. Pre-treatment with the antioxidant *N*-acetylcysteine (NAC) markedly reduced the proportion of ROS-positive cells under all conditions, indicating that 7k-induced oxidative stress is largely scavenger sensitive (Fig. 6A and B).

To determine whether this oxidative stress was associated with genotoxic damage, we assessed phosphorylation of histone H2AX (γH2AX), a sensitive marker of DNA double-strand breaks. Western blot analysis revealed a robust increase in γH2AX levels in 7k-treated MV4-11 cells relative to controls (Fig. 6C), consistent with ROS-associated DNA damage. These findings, together with the observed caspase-3/7 activation and increased apoptotic cell populations, suggest that 7k exerts its antiproliferative effects at least in part through ROS-dependent oxidative stress leading to DNA damage, G0/G1 cell-cycle arrest, and subsequent activation of caspase-dependent apoptosis.

### 2.6 Pharmacokinetic studies

The drug-likeness and pharmacokinetic suitability of representative compounds 7e, 7f, 7k, and 7r were evaluated using Lipinski's Rule of Five (Ro5) with ADMET predictions obtained from SwissADME (https://www.swissadme.ch/), ADMETmesh (https://admetmesh.scbdd.com/), and pkCSM (https://biosig.unimelb.edu.au/pkcsm/prediction). Lipinski's rule is widely applied to estimate oral bioavailability and early pharmacokinetic behaviour of small molecules. Table 3 summarizes various physicochemical properties of the representative compounds.

**Table 3:** Physicochemical properties of compounds 7e, 7f, 7k and 7r

	7e	7f	7k	7r
Molecular weight (MW)	702.36	716.387	702.36	746.413
TPSA (Å)	144.75	144.75	144.75	153.95
No. of rotatable bonds (nRot)	10	11	10	12
No. of HBA (nHA)	9	9	9	10
No. of HBD (nHD)	2	2	2	2
Log *P*	3.931	4.228	3.882	4.021

**Fig. 7:**
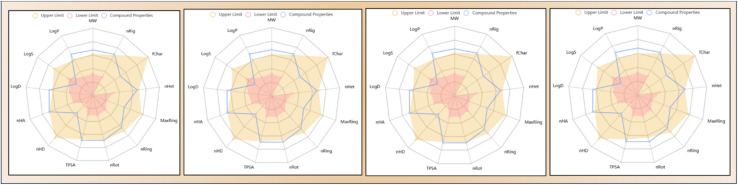
Radar view depicting deviations from typical Ro5 thresholds for compounds 7e, 7f, 7k and 7r.

According to Lipinski's Rule of Five (Ro5), compounds are considered drug-like when they possess molecular weight (MW) <500 Da, log *P* <5, fewer than 5 hydrogen bond donors (HBD), and fewer than 10 hydrogen bond acceptors (HBA). Overall, the evaluation of drug-likeness based on Lipinski's Rule of Five revealed that compounds 7e, 7f, 7k, and 7r exhibit favorable lipophilicity, acceptable hydrogen bonding capacity, and an appropriate number of rotatable bonds, however, they deviate from classical Ro5 criteria primarily due to their higher molecular weights and slightly elevated TPSA values. Therefore, placing these compounds within the beyond Rule of Five (bRo5) chemical space, which accommodates larger and more complex bioactive molecules with potentially suitable pharmacokinetic behaviour. Fig. 7 depicts deviations from typical Ro5 guidelines for compounds 7e, 7f, 7k and 7r. The predicted ADMET profiles further support their reasonable drug-like characteristics and pharmacokinetic feasibility. Nevertheless, further experimental studies are necessary to validate these predictions and fully establish their therapeutic viability.

## 3 Conclusion

In summary, a library of 1*H*-1,2,3-triazole-linked DHPM–isatin hybrids were designed, synthesized and assessed for their antiproliferative activity. All the synthesized hybrids exhibited enhanced antiproliferative potency and selectivity toward MV4-11 cells compared to LN229 and SHSY-5Y cells. Among the series, compounds bearing dibromo substituents at the C-5 and C-7 positions of the isatin moiety showed superior activity relative to their unsubstituted counterparts as well as the corresponding parent molecules. Moreover, the incorporation of longer alkyl chain linkers (*n* = 3, 4) further improved the biological activity, whereas variations in the DHPM ring substitutions had minimal impact on potency. Notably, compound 7k (IC_50_ = 0.8 ± 0.1 µM) demonstrated exceptional potency, particularly against MV4-11 acute myeloid leukemia cells, surpassing the reference drug cisplatin (IC_50_ = 4.5 ± 0.2 µM) while exhibiting reduced toxicity toward non-malignant HaCaT cells. Additionally, 7k displayed SI of 37.12 surpassing the standard cisplatin (SI = 7.62) demonstrating higher selectivity towards MV4-11 (acute myeloid leukemia) cancer cells. Mechanistic studies revealed that 7k induces G0/G1 phase cell cycle arrest leading to apoptosis. Additionally, compound 7k significantly elevated intracellular ROS levels, potentially leading to mitochondrial dysfunction and activation of intrinsic caspase-dependent apoptosis. Collectively, the mechanistic investigations confirm that compound 7k not only inhibits proliferation but actively triggers programmed cell death through interconnected oxidative and cell cycle regulatory pathways, highlighting its potent antiproliferative activity.

## 4 Experimental section

### 4.1 General information

^1^H NMR and ^13^C NMR spectra were recorded in deuterochloroform (CDCl_3_) with Bruker 500 (500 MHz) spectrometer using TMS as an internal standard. Chemical shift values are expressed as parts per million downfield from TMS, and *J* values are in hertz. Splitting patterns are indicated as s: singlet, d: doublet, t: triplet, m: multiplet, dd: double doublet, ddd: doublet of a doublet of a doublet, and br: broad peak. High-resolution mass spectra were recorded on a Bruker-micrOTOF-Q II spectrometer.

### 4.2 Synthesis of propargylated aldehyde precursors 2(a–c)

A stirred solution of the substituted aldehyde (1 equiv.) in DMF was treated with K_2_CO_3_ (1.5 equiv.) and heated at 100 °C for 30 minutes to generate the corresponding anion. Propargyl bromide (1.5 equiv.) was then added dropwise, and the reaction progress was monitored by TLC. After completion, water was added to the mixture, resulting in the formation of precipitates. The precipitates were filtered under high vacuum and air-dried to yield compounds 2(a–c).

### 4.3 Synthesis of propargylated DHPMs precursors 3(a–c)

A solution of thiourea (1 equiv), ethyl acetoacetate (1.5 equiv.) in ethanol and corresponding propargylated aldehyde (1 equiv.) with 1–2 drops of HCl was refluxed overnight. The reaction progress was monitored by TLC. Upon completion, the reaction mixture was cooled to room temperature and then placed in ice bath for 2 h, resulting in formation of precipitates. The resulting precipitates were filtered under high vacuum, washed with cold ethanol, and air-dried.

### 4.4 Synthesis of *N*-bromoalkyl-isatin precursors' 5(a–i)

To a solution of isatin in DMF and potassium carbonate was added at 80 °C for 30 min resulting in the formation of intense purple colored anion. Then, corresponding dibromoalkane was added to the reaction mixture and the mixture was stirred for about 1 h. The reaction was monitored by TLC. Upon completion, the reaction mixture was subjected to workup using ethyl acetate and water. The organic layer, extracted in ethyl acetate, was dried using anhydrous sodium sulphate to remove any residual aqueous content. Finally, the ethyl acetate was evaporated, yielding the desired precursors 5(a–f), which was then purified using column chromatography using ethyl acetate : hexane (20 : 80) mixture as eluent.

### 4.5 Synthesis of *N*-azidoalkyl-isatin precursors' 6(a–i)

The corresponding purified precursors 5a–f were then treated with sodium azide using DMF as solvent and the mixture was stirred for about 1 h at 80 °C. TLC determined the completion of the reaction. The reaction was monitored by TLC. Upon completion, ice-cold water was added to the reaction mixture following which the precipitates were then filtered and air-dried. The structure of the compound analysed with the help of spectral data.

### 4.6 Synthesis of DHPM–isatin hybrids 7(a–r)

A catalytic amount of CuSO_4_·5H_2_O and sodium ascorbate were added to a well-stirred solution of azidoalkyl isatin 6a (1 mmol) and propargylated naphthalimide 3a (1 mmol) in EtOH : H_2_O (8 : 2, v/v) mixture. The reaction was refluxed overnight and the progress of reaction was monitored by TLC. On completion, ice cold water was added leading to precipitation of desired conjugates. The resulting compounds were purified *via* column chromatography technique using silica gel (60–120 mesh) and ethylacetate : hexane (90 : 10) as the eluent mixture.

#### 4.6.1 Ethyl 4-(4-((1-(2-(2,3-dioxoindolin-1-yl)ethyl)-1*H*-1,2,3-triazol-4-yl)methoxy)phenyl)-6-methyl-2-oxo-1,2,3,4-tetrahydropyrimidine-5-carboxylate (7a)

Orange solid; mp: 104–106 °C; ^1^H NMR (500 MHz, CDCl_3_) *δ* 8.14 (s, 1H, –N**H**-exchangeable with D_2_O), 7.67 (s, 1H, triazole-**H**), 7.54 (d, *J* = 7.5 Hz, 1H, Ar–**H**), 7.38 (t, *J* = 7.2 Hz, 1H, Ar–**H**), 7.18 (d, *J* = 8.4 Hz, 2H, Ar–**H**), 7.03 (t, *J* = 7.5 Hz, 1H, Ar–**H**), 6.79 (d, *J* = 8.3 Hz, 2H, Ar–**H**), 6.52 (d, *J* = 7.9 Hz, 1H, Ar–**H**), 6.16 (s, 1H, –N**H**), 5.33 (s, 1H, –C**H**), 5.06 (s, 2H, O–C**H**_2_), 4.70 (t, *J* = 5.9 Hz, 2H, N–C**H**_2_), 4.23 (t, *J* = 5.9 Hz, 2H, N–C**H**2), 4.06 (q, *J* = 7.3 Hz, 2H, O–C**H**_2_–CH_3_), 2.32 (s, 3H, C–C**H**_3_), 1.16 (t, *J* = 7.1 Hz, 3H, O–CH_2_–C**H**_3_). ^13^C NMR (125 MHz, CDCl_3_) *δ* 183.25, 165.71, 158.43, 157.81, 150.49, 146.01, 144.31, 138.59, 136.75, 127.94, 125.66, 123.98, 122.83, 117.61, 114.88, 110.14, 101.61, 61.99, 60.07, 55.05, 49.43, 39.19, 18.68, 14.20. HRMS calcd for C_27_H_26_N_6_O_6_ [M + H]^+^ 531.1992 and found 531.1204.

#### 4.6.2 Ethyl 4-(4-((1-(3-(2,3-dioxoindolin-1-yl)propyl)-1*H*-1,2,3-triazol-4-yl)methoxy)phenyl)-6-methyl-2-oxo-1,2,3,4-tetrahydropyrimidine-5-carboxylate (7b)

Yellow solid; mp: 101–103 °C; ^1^H NMR (500 MHz, CDCl_3_) *δ* 8.03 (s, 1H, –N**H**-exchangeable with D_2_O), 7.79 (s, 1H, triazole-**H**), 7.61–7.57 (m, 2H, Ar–**H**), 7.22 (d, *J* = 8.3 Hz, 2H, Ar–**H**), 7.14 (t, *J* = 7.5 Hz, 1H, Ar–**H**), 6.91 (d, *J* = 6.4 Hz, 3H, Ar–**H**), 6.89 (s, 1H, Ar–**H**), 5.94 (s, 1H, –N**H**), 5.34 (s, 1H, –C**H**), 5.17 (s, 2H, O–C**H**_2_), 4.44 (t, *J* = 6.5 Hz, 2H, N–C**H**_2_), 4.09–4.04 (m, 2H, O–C**H**_2_–CH_3_), 3.79 (t, *J* = 6.7 Hz, 2H, N–C**H**_2_), 2.40 (p, *J* = 6.7 Hz, 2H, H_3_C–C**H**_2_–CH_3_), 2.33 (s, 3H, C–C**H**_3_), 1.16 (t, *J* = 7.1 Hz, 3H, O–CH_2_–C**H**_3_). ^13^C NMR (125 MHz, CDCl_3_) *δ* 182.95, 165.69, 158.64, 157.81, 150.20, 145.96, 144.12, 138.65, 136.74, 127.93, 125.70, 124.17, 123.79, 117.65, 114.92, 110.14, 101.61, 61.91, 60.06, 55.06, 47.67, 37.44, 27.74, 18.68, 14.20. HRMS calcd for C_28_H_28_N_6_O_6_ [M + H]^+^ 545.2148 and found 545.2157.

#### 4.6.3 Ethyl 4-(4-((1-(4-(2,3-dioxoindolin-1-yl)butyl)-1*H*-1,2,3-triazol-4-yl)methoxy)phenyl)-6-methyl-2-oxo-1,2,3,4-tetrahydropyrimidine-5-carboxylate (7c)

Orange solid; mp: 106–108 °C; ^1^H NMR (500 MHz, CDCl_3_) *δ* 8.05 (s, 1H, –N**H**-exchangeable with D_2_O), 7.55 (s, 1H, triazole-**H**), 7.54 (s, 1H, Ar–**H**), 7.53–7.49 (m, 1H, Ar–**H**), 7.15 (d, *J* = 8.4 Hz, 2H, Ar–**H**), 7.05 (t, *J* = 7.5 Hz, 1H, Ar–**H**), 6.82 (d, *J* = 8.1 Hz, 3H, Ar–**H**), 5.93 (s, 1H, –N**H**), 5.27 (s, 1H, –C**H**), 5.08 (s, 2H, O–C**H**_2_), 4.37 (t, *J* = 6.9 Hz, 2H, N–C**H**_2_), 4.02–3.97 (m, 2H, O–C**H**_2_–CH_3_), 3.68 (t, *J* = 7.0 Hz, 2H, N–C**H**_2_), 2.26 (s, 3H, C–C**H**_3_), 1.93 (p, *J* = 7.1 Hz, 2H, CH_2_–C**H**_2_), 1.66 (p, *J* = 7.1 Hz, 2H C**H**_2_–CH_2_), 1.09 (t, *J* = 7.1 Hz, 3H, O–CH_2_–C**H**_3_). ^13^C NMR (125 MHz, CDCl_3_) *δ* 183.24, 165.70, 158.42, 157.80, 150.48, 146.00, 144.30, 138.58, 136.74, 127.93, 125.65, 123.97, 122.82, 117.60, 114.87, 110.13, 101.60, 61.98, 60.06, 55.04, 49.42, 39.18, 27.39, 24.11, 18.67, 14.19. HRMS calcd for C_29_H_30_N_6_O_6_ [M + H]^+^ 559.2305 and found 559.2313.

#### 4.6.4 Ethyl 4-(4-((1-(2-(5,7-dibromo-2,3-dioxoindolin-1-yl)ethyl)-1*H*-1,2,3-triazol-4-yl)methoxy)phenyl)-6-methyl-2-oxo-1,2,3,4-tetrahydropyrimidine-5-carboxylate (7d)

Pale yellow solid; mp: 115–117 °C; ^1^H NMR (500 MHz, CDCl_3_) *δ* 7.78 (s, 1H, –N**H**-exchangeable with D_2_O), 7.61 (s, 1H, triazole-**H**), 7.53 (s, 1H, Ar–**H**), 7.16 (d, *J* = 8.4 Hz, 2H, Ar–**H**), 6.84 (d, *J* = 8.7 Hz, 2H, Ar–**H**), 6.81 (s, 1H, Ar–**H**), 5.38 (s, 1H, –N**H**), 5.29 (s, 1H, –C**H**), 5.12 (s, 2H, O–C**H**_2_), 4.37 (t, *J* = 6.9 Hz, 2H, N–C**H**_2_), 4.08 (t, *J* = 7.3 Hz, 2H, N–C**H**_2_), 4.02–3.99 (m, 2H, O–C**H**_2_–CH_3_), 2.28 (s, 3H, C–C**H**_3_), 1.11 (t, *J* = 7.1 Hz, 3H, O–CH_2_–C**H**_3_). ^13^C NMR (125 MHz, CDCl_3_) *δ* 183.22, 165.68, 158.40, 157.78, 150.46, 145.98, 144.28, 138.56, 136.72, 127.91, 125.63, 123.95, 122.80, 117.58, 114.85, 110.11, 101.58, 61.96, 60.04, 55.02, 49.40, 39.16, 18.65, 14.17. HRMS calcd for C_27_H_24_Br_2_N_6_O_6_ [M + H]^+^ 687.0202, [M + H + 2]^+^ 689.0128, [M + H + 4]^+^ 691.0161 and found 687.0213, 689.0134 and 691.0172.

#### 4.6.5 Ethyl 4-(4-((1-(3-(5,7-dibromo-2,3-dioxoindolin-1-yl)propyl)-1*H*-1,2,3-triazol-4-yl)methoxy)phenyl)-6-methyl-2-oxo-1,2,3,4-tetrahydropyrimidine-5-carboxylate (7e)

Orange solid; mp: 120–122 °C; ^1^H NMR (500 MHz, CDCl_3_) *δ* 7.87 (s, 1H, –N**H**-exchangeable with D_2_O), 7.74 (s, 1H, triazole-**H**), 7.70 (d, *J* = 2.0 Hz, 1H, Ar–**H**), 7.26 (d, *J* = 8.5 Hz, 3H, Ar–**H**), 6.94 (d, *J* = 8.6 Hz, 2H, Ar–**H**), 5.59 (s, 1H, –N**H**), 5.37 (s, 1H, –C**H**), 5.20 (s, 2H, O–C**H**_2_), 4.50 (t, *J* = 6.6 Hz, 2H, N–C**H**_2_), 4.23 (t, *J* = 7.1 Hz, 2H, N–C**H**_2_), 4.11–4.08 (m, 2H, O–C**H**_2_–CH_3_), 2.44 (p, *J* = 6.9 Hz, 2H, H_3_C–C**H**_2_–CH_3_), 2.37 (s, 3H, C–C**H**_3_), 1.20 (t, *J* = 7.1 Hz, 3H, O–CH_2_–C**H**_3_). ^13^C NMR (125 MHz, CDCl_3_) *δ* 182.97, 165.71, 158.66, 157.83, 150.22, 145.98, 144.14, 138.67, 136.76, 127.95, 125.72, 124.19, 123.81, 117.67, 114.94, 110.16, 101.63, 61.93, 60.08, 55.08, 47.69, 37.46, 27.76, 18.70, 14.22. HRMS calcd for C_28_H_26_Br_2_N_6_O_6_ [M + H]^+^ 701.0359, [M + H + 2]^+^ 703.0338, [M + H + 4]^+^ 705.0318 and found 701.0369, 703.0347 and 705.0326.

#### 4.6.6 Ethyl 4-(4-((1-(4-(5,7-dibromo-2,3-dioxoindolin-1-yl)butyl)-1*H*-1,2,3-triazol-4-yl)methoxy)phenyl)-6-methyl-2-oxo-1,2,3,4-tetrahydropyrimidine-5-carboxylate (7f)

Reddish-orange solid; mp: 113–115 °C; ^1^H NMR (500 MHz, CDCl_3_) *δ* 7.87 (s, 1H, –N**H**-exchangeable with D_2_O), 7.71 (s, 1H, triazole-**H**), 7.63 (s, 1H, Ar–**H**), 7.26 (d, *J* = 8.4 Hz, 2H, Ar–**H**), 6.94 (d, *J* = 8.7 Hz, 2H, Ar–**H**), 6.90 (s, 1H, Ar–**H**), 5.47 (s, 1H, –N**H**), 5.38 (s, 1H, –C**H**), 5.21 (s, 2H, O–C**H**_2_), 4.46 (t, *J* = 6.9 Hz, 2H, N–C**H**_2_), 4.18 (t, *J* = 7.3 Hz, 2H, N–C**H**_2_), 4.12–4.09 (m, 2H, O–C**H**_2_–CH_3_), 2.37 (s, 3H, C–C**H**_3_), 2.05–2.00 (m, 2H, H_2_C–C**H**_2_), 1.83–1.79 (m, 2H, **H**_2_C–CH_2_), 1.20 (t, *J* = 7.1 Hz, 3H, O–CH_2_–C**H**_3_). ^13^C NMR (125 MHz, CDCl_3_) *δ* 183.26, 165.72, 158.44, 157.82, 150.50, 146.02, 144.32, 138.60, 136.76, 127.95, 125.67, 123.99, 122.84, 117.62, 114.89, 110.15, 101.62, 62.00, 60.08, 55.06, 49.44, 39.20, 27.41, 24.13, 18.69, 14.21. HRMS calcd for C_29_H_28_Br_2_N_6_O_6_ [M + H]^+^ 715.0515, [M + H + 2]^+^ 717.0495, [M + H + 4]^+^ 719.0447 and found 715.0529, 717.0518 and 719.0454.

#### 4.6.7 Ethyl 4-(2-((1-(2-(2,3-dioxoindolin-1-yl)ethyl)-1*H*-1,2,3-triazol-4-yl)methoxy)phenyl)-6-methyl-2-oxo-1,2,3,4-tetrahydropyrimidine-5-carboxylate (7g)

Reddish-orange solid; mp: 107–109 °C; ^1^H NMR (500 MHz, CDCl_3_) *δ* 8.18 (s, 1H, –N**H**-exchangeable with D_2_O), 7.95 (s, 1H, triazole-**H**), 7.54 (d, *J* = 7.4 Hz, 1H, Ar–**H**), 7.41 (t, *J* = 7.8 Hz, 1H, Ar–**H**), 7.22 (t, *J* = 7.8 Hz, 1H, Ar–**H**), 7.09 (d, *J* = 7.5 Hz, 1H, Ar–**H**), 7.04 (t, *J* = 7.5 Hz, 1H, Ar–**H**), 6.97 (d, *J* = 8.2 Hz, 1H, Ar–**H**), 6.90 (t, *J* = 7.5 Hz, 1H, Ar–**H**), 6.56 (d, *J* = 8.0 Hz, 1H, Ar–**H**), 6.11 (s, 1H, –N**H**), 5.55 (s, 1H, –C**H**), 5.19 (s, 2H, O–C**H**_2_), 4.71 (t, *J* = 5.8 Hz, 2H, N–C**H**_2_), 4.26 (t, *J* = 5.7 Hz, 2H, N–C**H**_2_), 4.08–4.02 (m, 2H, O–C**H**2–CH_3_), 2.34 (s, 3H, C–C**H**_3_), 1.12 (t, *J* = 7.1 Hz, 3H, O–CH_2_–C**H**_3_). ^13^C NMR (125 MHz, CDCl_3_) *δ* 182.50, 165.79, 158.74, 155.66, 150.03, 147.55, 144.15, 138.52, 130.42, 129.22, 127.64, 125.54, 124.70, 124.12, 121.17, 117.44, 112.23, 109.48, 98.58, 61.88, 59.99, 50.83, 47.94, 40.67, 18.52, 14.17. HRMS calcd for C_27_H_26_N_6_O_6_ [M + H]^+^ 531.1992 and found 531.1201.

#### 4.6.8 Ethyl 4-(2-((1-(3-(2,3-dioxoindolin-1-yl)propyl)-1*H*-1,2,3-triazol-4-yl)methoxy)phenyl)-6-methyl-2-oxo-1,2,3,4-tetrahydropyrimidine-5-carboxylate (7h)

Pale yellow solid; mp: 193–195 °C; ^1^H NMR (500 MHz, CDCl_3_) *δ* 7.90 (s, 1H, –N**H**-exchangeable with D_2_O), 7.62 (dd, *J* = 14.7, 7.1 Hz, 2H, Ar–**H**), 7.30 (s, 1H, triazole-**H**), 7.25 (d, *J* = 8.0 Hz, 1H, Ar–**H**), 7.16 (t, *J* = 7.5 Hz, 1H, Ar–**H**), 7.11 (d, *J* = 7.6 Hz, 1H, Ar–**H**), 7.06 (d, *J* = 8.2 Hz, 1H, Ar–**H**), 6.94–6.90 (m, 2H, Ar–**H**), 6.07 (s, 1H, –N**H**), 5.73 (s, 1H, –C**H**), 5.31 (s, 2H, O–C**H**_2_), 4.46 (t, *J* = 6.5 Hz, 2H, N–C**H**_2_), 4.08–4.05 (m, 2H, O–C**H**_2_–CH_3_), 3.82 (t, *J* = 6.8 Hz, 2H, N–C**H**_2_), 2.44 (p, *J* = 7.2 Hz, 2H, H_3_C–C**H**_2_–CH_3_), 2.40 (s, 3H, C–C**H**_3_), 1.13 (t, *J* = 7.1 Hz, 3H, O–CH_2_–C**H**_3_). ^13^C NMR (125 MHz, CDCl_3_) *δ* 181.11, 165.93, 158.48, 155.78, 147.80, 146.25, 145.26, 143.92, 130.55, 129.33, 127.70, 127.59, 123.94, 121.49, 121.30, 117.37, 112.41, 104.82, 98.71, 62.06, 60.06, 50.82, 47.86, 39.09, 29.99, 18.75, 14.28. HRMS calcd for C_28_H_28_N_6_O_6_ [M + H]^+^ 545.2148 and found 545.2156.

#### 4.6.9 Ethyl 4-(2-((1-(4-(2,3-dioxoindolin-1-yl)butyl)-1*H*-1,2,3-triazol-4-yl)methoxy)phenyl)-6-methyl-2-oxo-1,2,3,4-tetrahydropyrimidine-5-carboxylate (7i)

Reddish-orange solid; mp: 96–98 °C; ^1^H NMR (500 MHz, CDCl_3_) *δ* 8.00 (s, 1H, –N**H**-exchangeable with D_2_O), 7.77 (s, 1H, triazole-**H**), 7.61 (t, *J* = 8.2 Hz, 2H, Ar–**H**), 7.22 (t, *J* = 7.8 Hz, 1H, Ar–**H**), 7.14 (t, *J* = 7.5 Hz, 1H, Ar–**H**), 7.08 (d, *J* = 7.6 Hz, 1H, Ar–**H**), 7.02 (d, *J* = 8.1 Hz, 1H, Ar–**H**), 6.89 (dd, *J* = 12.3, 7.7 Hz, 2H, Ar–**H**), 6.23 (s, 1H, –N**H**), 5.71 (s, 1H, –C**H**), 5.30 (s, 2H, O–C**H**_2_), 4.45 (t, *J* = 6.9 Hz, 2H, N–C**H**_2_), 4.08–4.04 (m, 2H, O–C**H**_2_–CH_3_), 3.72 (t, *J* = 7.0 Hz, 2H, N–C**H**_2_), 2.40 (s, 3H, C–C**H**_3_), 2.01 (p, *J* = 7.2 Hz, 2H, H_2_C–C**H**_2_), 1.72 (p, *J* = 7.3 Hz, 2H, **H**_2_C–CH_2_), 1.12 (t, *J* = 7.1 Hz, 3H, O–CH_2_–C**H**_3_). ^13^C NMR (125 MHz, CDCl_3_) *δ* 183.28, 165.82, 158.44, 155.58, 153.80, 150.50, 147.71, 143.98, 138.54, 130.37, 129.15, 127.35, 125.59, 123.92, 123.06, 121.18, 117.60, 112.38, 110.14, 98.63, 62.03, 59.98, 50.59, 49.47, 39.14, 27.22, 24.11, 18.65, 14.18. HRMS calcd for C_29_H_30_N_6_O_6_ [M + H]^+^ 559.2305 and found 559.2313.

#### 4.6.10 Ethyl 4-(2-((1-(2-(5,7-dibromo-2,3-dioxoindolin-1-yl)ethyl)-1*H*-1,2,3-triazol-4-yl)methoxy)phenyl)-6-methyl-2-oxo-1,2,3,4-tetrahydropyrimidine-5-carboxylate (7j)

Pale yellow solid; mp: 170–172 °C; ^1^H NMR (500 MHz, CDCl_3_) *δ* 7.83 (s, 1H, –N**H**-exchangeable with D_2_O), 7.79 (s, 1H, triazole-**H**), 7.75 (d, *J* = 2.1 Hz, 1H, Ar–**H**), 7.57 (d, *J* = 2.0 Hz, 1H, Ar–**H**), 7.16 (t, *J* = 7.0 Hz, 1H, Ar–**H**), 7.01 (d, *J* = 7.6 Hz, 1H, Ar–**H**), 6.96 (d, *J* = 8.2 Hz, 1H, Ar–**H**), 6.82 (t, *J* = 7.5 Hz, 1H, Ar–**H**), 6.11 (s, 1H, –N**H**), 5.63 (s, 1H, –C**H**), 5.18 (s, 2H, O–C**H**_2_), 4.39 (t, *J* = 6.6 Hz, 2H, N–C**H**_2_), 4.12 (t, *J* = 7.1 Hz, 2H, N–C**H**_2_), 3.98–3.94 (m, 2H, O–C**H**_2_–CH_3_), 2.28 (s, 3H, C–C**H**_3_), 1.03 (t, *J* = 7.1 Hz, 3H, O–CH_2_–C**H**_3_). ^13^C NMR (125 MHz, CDCl_3_) *δ* 182.51, 165.80, 158.75, 155.67, 150.04, 147.56, 144.16, 138.53, 130.43, 129.23, 127.65, 125.55, 124.71, 124.13, 121.18, 117.45, 112.24, 109.49, 98.59, 61.89, 60.00, 50.84, 47.95, 40.68, 18.53, 14.18. HRMS calcd for C_27_H_24_Br_2_N_6_O_6_ [M + H]^+^ 687.0202, [M + H + 2]^+^ 689.0128, [M + H + 4]^+^ 691.01613 and found 687.0207, 689.0137 and 691.0128.

#### 4.6.11 Ethyl 4-(2-((1-(3-(5,7-dibromo-2,3-dioxoindolin-1-yl)propyl)-1*H*-1,2,3-triazol-4-yl)methoxy)phenyl)-6-methyl-2-oxo-1,2,3,4-tetrahydropyrimidine-5-carboxylate (7k)

Orange solid; mp: 116–118 °C; ^1^H NMR (500 MHz, CDCl_3_) ^1^H NMR (500 MHz, CDCl_3_) *δ* 7.83 (s, 1H, –N**H**-exchangeable with D_2_O), 7.79 (s, 1H, triazole-**H**), 7.78–7.69 (m, 1H, Ar–**H**), 7.57 (d, *J* = 2.0 Hz, 1H, Ar–**H**), 7.16 (t, *J* = 7.0 Hz, 1H, Ar–**H**), 7.01 (d, *J* = 7.6 Hz, 1H, Ar–**H**), 6.96 (d, *J* = 8.2 Hz, 1H, Ar–**H**), 6.82 (t, *J* = 7.5 Hz, 1H, Ar–**H**), 6.11 (s, 1H, –N**H**), 5.63 (s, 1H, –C**H**), 5.18 (s, 2H, O–C**H**_2_), 4.39 (t, *J* = 6.6 Hz, 2H, N–C**H**_2_), 4.12 (t, *J* = 7.1 Hz, 2H, N–C**H**_2_), 4.04–3.88 (m, 2H, O–C**H**_2_–CH_3_), 2.34 (p, 2H, H_3_C–C**H**_2_–CH_3_), 2.28 (s, 3H, C–C**H**_3_), 1.03 (t, *J* = 7.1 Hz, 3H, O–CH_2_–C**H**_3_). ^13^C NMR (125 MHz, CDCl_3_) *δ* 181.13, 165.95, 158.50, 155.80, 147.82, 146.27, 145.28, 143.94, 130.57, 129.35, 127.72, 127.61, 123.96, 121.51, 121.32, 117.39, 112.43, 104.84, 98.73, 62.08, 60.08, 50.84, 47.88, 39.11, 30.01, 18.77, 14.30. HRMS calcd for C_28_H_26_Br_2_N_6_O_6_ [M + H]^+^ 701.0359, [M + H + 2]^+^ 703.0338, [M + H + 4]^+^ 705.0318 and found 701.0368, 703.0347 and 705.0329.

#### 4.6.12 Ethyl 4-(2-((1-(4-(5,7-dibromo-2,3-dioxoindolin-1-yl)butyl)-1*H*-1,2,3-triazol-4-yl)methoxy)phenyl)-6-methyl-2-oxo-1,2,3,4-tetrahydropyrimidine-5-carboxylate (7l)

Orange solid; mp: 112–114 °C; ^1^H NMR (500 MHz, CDCl_3_) *δ* 7.84 (s, 1H, –N**H**-exchangeable with D_2_O), 7.77 (s, 1H, triazole-**H**), 7.68 (d, *J* = 2.1 Hz, 1H, Ar–**H**), 7.42 (s, 1H, Ar–**H**), 7.23 (t, *J* = 8.7 Hz, 1H, Ar–**H**), 7.08 (d, *J* = 5.9 Hz, 1H, Ar–**H**), 7.04 (d, *J* = 8.2 Hz, 1H, Ar–**H**), 6.90 (t, *J* = 7.5 Hz, 1H, Ar–**H**), 6.03 (s, 1H, –N**H**), 5.70 (s, 1H, –C**H**), 5.30 (s, 2H, O–C**H**_2_), 4.45 (t, *J* = 6.9 Hz, 2H, N–C**H**_2_), 4.12 (t, *J* = 7.5 Hz, 2H, N–C**H**_2_), 4.09–4.05 (m, 2H, O–C**H**_2_–CH_3_), 2.40 (s, 3H, C–C**H**_3_), 2.03 (p, 2H, C**H**_2_–CH_2_), 1.79 (p, 2H, H_2_C–C**H**_2_), 1.13 (t, *J* = 7.1 Hz, 3H, O–CH_2_–C**H**_3_). ^13^C NMR (125 MHz, CDCl_3_) *δ* 181.33, 165.81, 158.20, 155.64, 147.58, 146.42, 145.14, 143.86, 130.30, 129.17, 127.56, 127.36, 123.12, 121.42, 121.12, 117.06, 112.31, 104.64, 98.64, 61.96, 59.99, 50.78, 49.68, 40.49, 26.97, 26.50, 18.79, 14.19. HRMS calcd for C_29_H_28_Br_2_N_6_O_6_ [M + H]^+^ 715.0515, [M + H + 2]^+^ 717.0495, [M + H + 4]^+^ 719.0474 and found 715.0524, 717.0504 and 719.0486.

#### 4.6.13 Ethyl 4-(4-((1-(2-(2,3-dioxoindolin-1-yl)ethyl)-1*H*-1,2,3-triazol-4-yl)methoxy)-3-methoxyphenyl)-6-methyl-2-oxo-1,2,3,4-tetrahydropyrimidine-5-carboxylate (7m)

Orange solid; mp: 135–137 °C; ^1^H NMR (500 MHz, CDCl_3_) *δ* 7.93 (s, 1H, –N**H**-exchangeable with D_2_O), 7.67 (s, 1H, triazole-**H**), 7.58–7.54 (m, 1H, Ar–**H**), 7.38 (td, *J* = 7.8, 1.3 Hz, 1H, Ar–**H**), 7.04 (t, *J* = 7.5 Hz, 1H, Ar–**H**), 6.85–6.81 (m, 2H, Ar–**H**), 6.78 (dd, *J* = 8.2, 2.1 Hz, 1H, Ar–**H**), 6.55 (d, *J* = 8.0 Hz, 1H, Ar–**H**), 6.06 (s, 1H, –N**H**), 5.37 (s, 1H, –C**H**), 5.17 (s, 2H, O–C**H**_2_), 4.70 (t, *J* = 6.0 Hz, 2H, N–C**H**_2_), 4.24 (t, *J* = 5.9 Hz, 2H, N–C**H**_2_), 4.10 (q, *J* = 7.1 Hz, 2H, O–C**H**_2_–CH_3_), 3.79 (s, 3H, O–C**H**_3_), 2.36 (s, 3H, C–C**H**_3_), 1.20 (t, *J* = 7.1 Hz, 3H, O–CH_2_–C**H**_3_). ^13^C NMR (125 MHz, CDCl_3_) *δ* 183.26, 165.73, 158.41, 153.70, 150.51, 149.58, 147.20, 145.98, 144.43, 138.58, 137.38, 125.65, 123.96, 122.99, 118.78, 117.60, 114.10, 110.16, 101.56, 63.11, 60.13, 55.93, 55.26, 49.42, 39.22, 18.67, 14.25. HRMS calcd for C_28_H_28_N_6_O_7_ [M + H]^+^ 561.2097 and found 561.2105.

#### 4.6.14 Ethyl 4-(4-((1-(3-(2,3-dioxoindolin-1-yl)propyl)-1*H*-1,2,3-triazol-4-yl)methoxy)-2-methoxyphenyl)-2-oxo-1,2,3,4-tetrahydropyrimidine-5-carboxylate (7n)

Orange solid; mp: 116–118 °C; ^1^H NMR (500 MHz, CDCl_3_) *δ* 7.87 (s, 1H, –N**H**-exchangeable with D_2_O), 7.73 (s, 1H, triazole-**H**), 7.71 (d, *J* = 2.1 Hz, 1H, Ar–**H**), 7.30 (s, 3H, Ar–**H**), 6.98 (d, *J* = 7.8 Hz, 1H, Ar–**H**), 6.86 (d, *J* = 2.4 Hz, 2H, Ar–**H**), 6.68 (s, 1H, Ar–**H**), 5.41 (s, 1H, –N**H**), 5.37 (s, 1H, –C**H**), 5.29 (s, 2H, O–C**H**_2_), 4.48 (t, *J* = 6.7 Hz, 2H, N–C**H**_2_), 4.23–4.20 (m, 2H, N–C**H**_2_), 4.13–4.10 (m, 2H, O–C**H**_2_–CH_3_), 3.87 (s, 3H, O–C**H**_3_), 2.44–2.40 (m, 2H, H_3_C–C**H**_2_–CH_3_), 2.37 (s, 3H, C–C**H**_3_), 1.21 (t, 3H, O–CH_2_–C**H**_3_). ^13^C NMR (125 MHz, CDCl_3_) *δ* 183.23, 165.70, 158.38, 153.67, 150.48, 149.54, 147.17, 145.95, 144.39, 138.55, 137.35, 125.61, 123.92, 122.96, 118.75, 117.57, 114.07, 110.13, 101.53, 63.07, 60.10, 55.89, 55.23, 49.39, 39.19, 24.12, 18.64, 14.22. HRMS calcd for C_29_H_30_N_6_O_7_ [M + H]^+^ 575.2254 and found 575.2263.

#### 4.6.15 Ethyl 4-(4-((1-(4-(2,3-dioxoindolin-1-yl)butyl)-1*H*-1,2,3-triazol-4-yl)methoxy)-3-methoxyphenyl)-6-methyl-2-oxo-1,2,3,4-tetrahydropyrimidine-5-carboxylate (7o)

Orange solid; mp: 118–120 °C; ^1^H NMR (500 MHz, CDCl_3_) *δ* 8.21 (s, 1H, –N**H**-exchangeable with D_2_O), 7.65 (s, 1H, triazole-**H**), 7.62 (d, *J* = 7.4 Hz, 1H, Ar–**H**), 7.14 (t, *J* = 7.5 Hz, 1H, Ar–**H**), 6.94 (d, *J* = 8.2 Hz, 1H, Ar–**H**), 6.90 (d, *J* = 7.8 Hz, 1H, Ar–**H**), 6.85 (s, 1H, Ar–**H**), 6.82 (d, *J* = 8.3 Hz, 1H, Ar–**H**), 6.17 (s, 1H, –N**H**), 5.37 (s, 1H, –C**H**), 5.25 (s, 2H, O–C**H**_2_), 4.43 (t, *J* = 6.9 Hz, 2H, N–C**H**_2_), 4.10 (q, *J* = 7.1 Hz, 2H, O–C**H**_2_–CH_3_), 3.82 (s, 3H, O–C**H**_3_), 3.76 (t, *J* = 7.0 Hz, 2H, N–C**H**_2_), 2.36 (s, 3H, C–C**H**_3_), 2.00 (p, *J* = 7.1 Hz, 2H, CH_2_–C**H**_2_), 1.74 (p, *J* = 7.2 Hz, 2H, C**H**_2_–CH_2_), 1.19 (t, *J* = 7.1 Hz, 3H, O–CH_2_–C**H**_3_). ^13^C NMR (125 MHz, CDCl_3_) *δ* 183.25, 165.72, 158.40, 153.69, 150.50, 149.56, 147.19, 145.97, 144.41, 138.57, 137.37, 125.63, 123.94, 122.98, 118.77, 117.59, 114.09, 110.15, 101.55, 63.09, 60.12, 55.91, 55.25, 49.41, 39.21, 27.39, 24.14, 18.66, 14.24. HRMS calcd for C_30_H_32_N_6_O_7_ [M + H]^+^ 589.2410 and found 589.2421.

#### 4.6.16 Ethyl 4-(4-((1-(2-(5,7-dibromo-2,3-dioxoindolin-1-yl)ethyl)-1*H*-1,2,3-triazol-4-yl)methoxy)-3-(l1-oxidanyl)phenyl)-6-methyl-2-oxo-1,2,3,4-tetrahydropyrimidine-5-carboxylate (7p)

Yellow solid; mp: 120–121 °C; ^1^H NMR (500 MHz, CDCl_3_) *δ* 7.87 (s, 1H, –N**H**-exchangeable with D_2_O), 7.73 (s, 1H, triazole-**H**), 7.71 (d, *J* = 2.1 Hz, 1H, Ar–**H**), 7.30 (s, 1H, Ar–**H**), 6.98 (d, *J* = 7.8 Hz, 1H, Ar–**H**), 6.87 (d, *J* = 2.4 Hz, 2H, Ar–**H**), 6.68 (s, 1H, Ar–**H**), 5.41 (s, 1H, –N**H**), 5.37 (s, 1H, –C**H**), 5.29 (s, 2H, O–C**H**_2_), 4.48 (t, *J* = 6.7 Hz, 2H, N–C**H**_2_), 4.23–4.20 (m, 2H, N–C**H**_2_), 4.11 (q, *J* = 7.1 Hz, 2H, O–C**H**_2_–CH_3_), 3.87 (s, 3H, O–C**H**_3_), 2.37 (s, 3H, C–C**H**_3_), 1.21 (s, 3H, O–CH_2_–C**H**_3_). ^13^C NMR (125 MHz, CDCl_3_) *δ* 181.28, 165.70, 158.10, 153.46, 149.54, 147.21, 146.42, 145.95, 145.17, 144.35, 137.34, 127.61, 122.92, 121.36, 118.79, 117.09, 114.07, 110.38, 104.65, 101.53, 63.09, 60.09, 55.92, 55.27, 49.61, 40.54, 18.69, 14.22. HRMS calcd for C_27_H_23_Br_2_N_6_O_7_ [M + H]^+^ 702.0073, [M + H + 2]^+^ 704.0053, [M + H + 4]^+^ 706.0032 and found 702.0085, 704.0064 and 706.0043.

#### 4.6.17 Ethyl 4-(4-((1-(3-(5,7-dibromo-2,3-dioxoindolin-1-yl)propyl)-1*H*-1,2,3-triazol-4-yl)methoxy)-3-methoxyphenyl)-6-methyl-2-oxo-1,2,3,4-tetrahydropyrimidine-5-carboxylate (7q)

Orange solid; mp: 199–201 °C; ^1^H NMR (500 MHz, CDCl_3_) *δ* 7.87 (s, 1H, –N**H**-exchangeable with D_2_O), 7.73 (s, 1H, triazole-**H**), 7.71 (d, *J* = 2.1 Hz, 1H, Ar–**H**), 7.30 (s, 1H, Ar–**H**), 6.98 (d, *J* = 7.8 Hz, 1H, Ar–**H**), 6.87 (d, *J* = 2.4 Hz, 2H, Ar–**H**), 6.68 (s, 1H, Ar–**H**), 5.41 (s, 1H, –N**H**), 5.37 (s, 1H, –C**H**), 5.29 (s, 2H, O–C**H**_2_), 4.48 (t, *J* = 6.7 Hz, 2H, N–C**H**_2_), 4.23–4.20 (m, 2H, N–C**H**_2_), 4.13–4.09 (m, 2H, O–C**H**_2_–CH_3_), 3.87 (s, 3H, O–C**H**_3_), 2.44–2.40 (m, 2H, H_3_C–C**H**_2_–CH_3_), 2.37 (s, 3H, C–C**H**_3_), 1.21 (s, 3H, O–CH_2_–C**H**_3_). ^13^C NMR (125 MHz, CDCl_3_) *δ* 181.32, 165.74, 158.14, 153.50, 149.58, 147.25, 146.46, 145.99, 145.21, 144.39, 137.38, 127.65, 122.96, 121.40, 118.83, 117.13, 114.11, 110.42, 104.69, 101.57, 63.13, 60.13, 55.96, 55.31, 49.65, 40.58, 26.57, 18.73, 14.26. HRMS calcd for C_29_H_28_Br_2_N_6_O_7_ [M + H]^+^ 731.0464, [M + H + 2]^+^ 733.0444, [M + H + 4]^+^ 735.0423 and found 731.0472, 733.0453 and 735.0435.

#### 4.6.18 Ethyl 4-(4-((1-(4-(5,7-dibromo-2,3-dioxoindolin-1-yl)butyl)-1*H*-1,2,3-triazol-4-yl)methoxy)-3-methoxyphenyl)-6-methyl-2-oxo-1,2,3,4-tetrahydropyrimidine-5-carboxylate (7r)

Reddish-orange solid; mp: 114–116 °C; ^1^H NMR (500 MHz, CDCl_3_) *δ* 8.06 (s, 1H, –N**H**-exchangeable with D_2_O), 7.85 (d, *J* = 2.0 Hz, 1H, Ar–**H**), 7.68 (d, *J* = 2.0 Hz, 1H, Ar–**H**), 7.66 (s, 1H, triazole-**H**), 7.29 (s, 1H, Ar–**H**), 6.95 (d, *J* = 8.2 Hz, 1H, Ar–**H**), 6.87–6.81 (m, 2H, Ar–**H**), 6.07 (s, 1H, –N**H**), 5.37 (s, 1H, –C**H**), 5.25 (s, 2H, O–C**H**_2_), 4.42 (t, *J* = 7.0 Hz, 2H, N–C**H**_2_), 4.15 (t, *J* = 7.3 Hz, 2H, N–C**H**_2_), 4.10 (q, *J* = 7.0 Hz, 2H, O–C**H**_2_–CH_3_), 3.83 (s, 3H, O–C**H**_3_), 2.36 (s, 3H, C–C**H**_3_), 2.00 (p, *J* = 7.2 Hz, 2H, CH_2_–C**H**_2_), 1.78 (p, *J* = 7.6 Hz, 2H, C**H**_2_–CH_2_), 1.20 (t, *J* = 7.1 Hz, 3H, O–CH_2_–C**H**_3_). ^13^C NMR (125 MHz, CDCl_3_) *δ* 181.30, 165.72, 158.12, 153.48, 149.56, 147.23, 146.44, 145.97, 145.19, 144.37, 137.36, 127.63, 122.94, 121.38, 118.81, 117.11, 114.09, 110.40, 104.67, 101.55, 63.11, 60.11, 55.94, 55.29, 49.63, 40.56, 27.14, 26.55, 18.71, 14.24. HRMS calcd for C_30_H_30_Br_2_N_6_O_7_ [M + H]^+^ 745.0621, [M + H + 2]^+^ 747.0600, [M + H + 4]^+^ 749.058 and found 745.0632, 747.0611 and 749.067.

## 5 Materials and methods

### 5.1 Cell culture conditions

All cell lines were obtained from the American Type Culture Collection (ATCC, Manassas, VA, USA) and maintained in tissue culture flasks at 37 °C in a humidified atmosphere with 5% CO_2_. The cell lines were grown in DMEM (ThermoFisher Scientific, USA) supplemented with 10% FBS (Gibco) and 1% antibiotics (10.00 units per mL penicillin and 10.00 mg mL^−1^ streptomycin; Gibco). Compound stock solutions were prepared and administered to cells in DMSO. All the cell lines were purchased from ATCC, USA. The antibodies were purchased from Cell Signaling Technologies, USA.

### 5.2 Cell viability assay

The synthesized derivatives were initially dissolved in dimethyl sulfoxide (DMSO) to prepare 10^−2^ M stock solutions, from which serial dilutions were made. Each compound was first screened for antitumor activity at a single concentration of 25 µM using MV4-11, LN229, and SHSY-5Y cancer cell lines. Compounds showing promising inhibitory effects were subsequently evaluated over a concentration range of 1–50 µM in MV4-11, LN229, and SHSY-5Y cells cancer cells, along with HaCaT non-malignant keratinocytes to assess selectivity.

Briefly, cells were seeded in 96-well plates at a density of 1 × 10^4^ cells per well and allowed to adhere for 24 h. Thereafter, they were treated with either vehicle control (DMSO) or the indicated compound concentrations for 48 h, each condition tested in triplicate. Four hours before the end of the incubation period, 20 µL of MTS (3-(4,5-dimethylthiazol-2-yl)-5-(3-carboxymethoxyphenyl)-2-(4-sulfophenyl)-2*H*-tetrazolium; Promega, WI, USA) reagent (5 mg mL^−1^) was added to each well, followed by incubation at 37 °C to allow formazan crystal formation. Absorbance was measured at 490 nm using a microplate reader.

Half-maximal inhibitory concentrations (IC_50_) were calculated using nonlinear regression analysis in GraphPad Prism 7.0 software. Graphical representations were generated using GraphPad Prism 7.0, and all data represents the mean ± SD from at least three independent experiments performed in triplicate.

### 5.3 Apoptosis assays

The pro-apoptotic potential of compound 7k was evaluated using Annexin V & Dead Cell and caspase-3/7 assay kits (EMD Millipore, Darmstadt, Germany). MV4-11 cells were seeded in 6-well plates at a density of 3 × 10^5^ cells per well and cultured at 37 °C in a humidified atmosphere containing 5% CO_2_. After 24 h of incubation, cells were treated with either DMSO (vehicle control) or with 7k at IC_50_ or twice the IC_50_ (2 × IC_50_) values. Treatments were carried out for 48 h. Following incubation, cells were collected and stained with the respective dyes according to the manufacturer's instructions. The samples were analyzed using a Muse™ Cell Analyzer (Merck Millipore, Darmstadt, Germany) equipped with Muse™ software version 1.4. Graphical data visualization was performed using GraphPad Prism 7.0, and results represent mean values from three independent experiments, each performed in triplicate. Error bars indicate standard deviations.

### 5.4 Cell cycle analysis

Cell cycle distribution was analyzed using the Muse™ Cell Cycle Assay Kit (EMD Millipore, Darmstadt, Germany) according to the manufacturer's instructions. MV4-11 cells were seeded in 6-well plates at a density of 3 × 10^5^ cells per well and allowed to incubate for 24 h at 37 °C in a humidified atmosphere containing 5% CO_2_. Cells were then treated with DMSO (vehicle control) or compound 7k at IC_50_ and 2 × IC_50_ concentrations for 48 h. Following treatment, cells were harvested, washed with PBS, and fixed in 70% cold ethanol for at least 3 h at −20 °C. Fixed cells were subsequently washed and stained with the Muse™ Cell Cycle reagent containing propidium iodide (PI) and RNase A to allow DNA content analysis. Samples were incubated at room temperature in the dark for 30 min and analyzed using a Muse™ Cell Analyzer equipped with Muse™ software version 1.4. The percentage of cells in G0/G1, S, and G2/M phases was quantified. All experiments were performed in triplicate, and data are presented as mean ± SD from at least three independent experiments.

### 5.5 Reactive oxygen species (ROS) analysis

Intracellular reactive oxygen species (ROS) levels were determined using the Muse™ Oxidative Stress Assay Kit (EMD Millipore, Darmstadt, Germany). MV4-11 cells were seeded at a density of 3 × 10^5^ cells per well in 6-well plates and incubated for 24 h prior to treatment. Cells were then treated for 48 h with DMSO (vehicle control) or compound 7k at IC_50_ and 2 × IC_50_ concentrations, pre-treated with or without 5 mM of *N*-acetylcysteine (NAC) in the serum-enriched medium for 24 h. After treatment, cells were collected, washed with PBS, and incubated with the oxidative stress reagent, which detects intracellular superoxide production. Following 30 min incubation at 37 °C in the dark, samples were immediately analyzed using a Muse™ Cell Analyzer. The percentage of ROS-positive cells was determined using Muse™ analysis software. All experiments were performed independently at least three times in triplicate. Results are presented as mean ± SD.

### 5.6 Western blot analysis of γH2AX

MV4-11 cells were seeded in 6-well plates and allowed to recover overnight, then treated with vehicle (0.1% DMSO) or compound 7k at its IC_50_ concentration for 48 h. After treatment, cells were harvested, washed twice with cold phosphate-buffered saline (PBS), and lysed on ice in RIPA buffer (50 mM Tris–HCl, pH 7.4, 150 mM NaCl, 1% NP-40, 0.5% sodium deoxycholate, 0.1% SDS) supplemented with protease and phosphatase inhibitor cocktails. Lysates were incubated on ice for 30 min with periodic vortexing and clarified by centrifugation at 12 000×*g* for 15 min at 4 °C. Protein concentrations were determined using the bicinchoninic acid (BCA) assay. Equal amounts of protein (20–30 µg per lane) were resolved by SDS-PAGE on 10–12% polyacrylamide gels and electrotransferred onto PVDF membranes.

Membranes were blocked for 1 h at room temperature in Tris-buffered saline containing 0.1% Tween-20 (TBST) and 5% non-fat dry milk, then incubated overnight at 4 °C with a primary antibody against phospho-histone H2AX (γH2AX, Ser139) diluted 1 : 1000 in TBST containing 5% bovine serum albumin (BSA). After three washes in TBST, membranes were incubated for 1 h at room temperature with HRP-conjugated secondary antibody (1 : 5000 in TBST with 5% milk). Immunoreactive bands were visualized using enhanced chemiluminescence (ECL) and captured on a chemiluminescence imaging system. To verify equal loading, membranes were stripped and reprobed with an *anti*-α-tubulin antibody (1 : 5000), followed by HRP-conjugated secondary antibody and ECL detection.

### 5.7 Statistical analysis

All quantitative data are presented as mean ± standard deviation (SD) from at least three independent experiments, each performed in triplicate unless otherwise specified. For comparisons involving more than two groups (*e.g.*, cell-viability assays, ROS measurements, apoptosis, and cell-cycle distributions), statistical significance was assessed using one-way analysis of variance (ANOVA) followed by Dunnett's post-hoc test, with the vehicle-treated group as the reference. For pairwise comparisons between two groups (*e.g.*, control *vs.* treated in western blot densitometry), an unpaired two-tailed Student's *t*-test was applied. Statistical analyses were performed using GraphPad Prism (version X; GraphPad Software, San Diego, CA, USA). A *p* value < 0.05 was considered statistically significant, with significance levels denoted in the figures as *p* < 0.05 (*), *p* < 0.01 (**), and *p* < 0.001 (***).

## Author contributions

Preeti – synthesis, molecular docking studies, writing (original draft); Asif Raza – biological studies, reviewing the draft; Jashandeep Kaur – synthesis; Arun K Sharma – biological studies, writing (review and editing). Vipan Kumar – conceptualization and designing the work and writing (review and editing).

## Conflicts of interest

There are no conflicts to declare.

## Supplementary Material

RA-OLF-D6RA03517E-s001

## Data Availability

The data associated with this submission is available as electronic supplementary information (SI). Supplementary information is available. See DOI: https://doi.org/10.1039/d6ra03517e.
