# Supplementary material for: Dihydropyrimidinone–isatin hybrids as potent inducers of cell-cycle arrest and caspase-dependent apoptosis in MV4-11 acute myeloid leukemia cells
Source: RSC Adv. 2026 Aug 5;16(42):50055–68. doi: 10.1039/d6ra03517e (PMC13439381; doi:10.1039/d6ra03517e)
Supplement: Supplementary file 1 [file RA-016-D6RA03517E-s001.pdf]

Supplementary Information (SI) for RSC Advances.
This journal is © The Royal Society of Chemistry 2026

Dihydropyrimidinone–Isatin Hybrids as Potent Inducers of Cell-Cycle Arrest and Caspase-Dependent Apoptosis in MV4-11 Acute Myeloid Leukemia Cells

Preeti[a,1], Asif Raza[b,1], Jashandeep Kaur[a], Arun K Sharma[*b], Vipan Kumar[*a]

[a] Department of Chemistry, Guru Nanak Dev University, Amritsar 143005, Punjab, India.
[b] Department of Molecular and Precision Medicine, Penn State Cancer Institute, The Pennsylvania State University College of Medicine, Hershey, PA 17033.
*Corresponding authors (asharma1@pennstatehealth.psu.edu (A.K. Sharma), vipan_org@yahoo.com (Vipan Kumar)
[1] These authors contributed equally

Contents

1. Molecular Docking
2. Molecular Docking Protocols
3. Scanned copies of ^{1}H and ^{13}C NMR spectra of synthesized compounds.
4. References

1. Molecular Docking

Cyclin-dependent kinases (CDKs) are key regulators of the cell cycle and play a crucial role in controlling several fundamental cellular processes, including apoptosis, transcription, and exocytosis. Owing to their central involvement in cell proliferation and survival, CDKs have emerged as highly attractive molecular targets for the development of chemotherapeutic agents. Inhibition of these kinases can effectively disrupt uncontrolled cell division and trigger programmed cell death, making them valuable targets in chemotherapy.[1]

The significant antiproliferative activity of compounds **7e**, **7f**, **7k**, and **7r**, together with their ability to disrupt cell cycle progression and promote apoptosis, encouraged further investigation of their binding interactions with the CDK2 protein kinase. Sunitinib, a known CDK inhibitor, was used as a reference ligand in this study. The primary aim of this study was to gain deeper insights into the molecular recognition and binding modes of the synthesized compounds within the CDK2 active site. For this purpose, the three-dimensional crystal structure of CDK2 was obtained from the Protein Data Bank (PDB ID: 2BHE). The 2D and 3D representation in **Figure 1** provides a clear visualization of various types of observed interactions between the studied compounds and the protein's active sites while **Table 1** summarizes the docking calculations.

The docking study revealed that all compounds exhibited favorable interactions within the active site of CDK2, such as hydrophobic, polar, and electrostatic interactions, as depicted in

Figure 1. For **7e**, hydrogen bond was observed between LYS20 amino acid residue and carbonyl oxygen of the DHPM ring. In case **7f**, two hydrogen bonding interactions were observed: one between GLY13 amino acid residue and keto oxygen of isatin ring and another between LYS20 amino acid residue and carbonyl oxygen of the DHPM ring, suggesting enhanced binding stabilization through multiple interactions. For compound **7k**, π-π stacking interaction was identified between the benzene ring linked to the DHPM core and the aromatic residue PHE80, highlighting the importance of aromatic interactions in reinforcing ligand binding. Meanwhile compound **7r**, exhibited three hydrogen bonding interactions: between GLY13 amino acid residue and keto oxygen of isatin, ILE10 amino acid residue and NH of DHPM ring and LEU83 amino acid residue and N of triazole ring which collectively contribute to its strong binding affinity.

The enhanced activity of the 5,7-dibromo-substituted derivatives may be partly explained by their binding mode within the CDK2 active site. Docking analysis predicted that the dibrominated isatin ring is oriented toward the solvent-exposed region of the CDK2 binding pocket, minimizing steric interference and promoting a favorable ligand conformation for optimal active-site interactions. Furthermore, the increased lipophilicity and polarizability imparted by the bromine atoms may enhance hydrophobic and van der Waals contacts. These observations provide a plausible structural basis for the enhanced antiproliferative activity exhibited by the 5,7-dibromo-substituted analogues. Overall, the docking results indicate that the synthesized compounds establish stable and energetically favorable interactions within the CDK2 active site. Notably, their docking scores were found to be comparable to that of Sunitinib, supporting CDK2 as a plausible molecular target, however, biochemical kinase assays will be required to determine whether these hybrids truly inhibit CDK2 activity and to what extent.

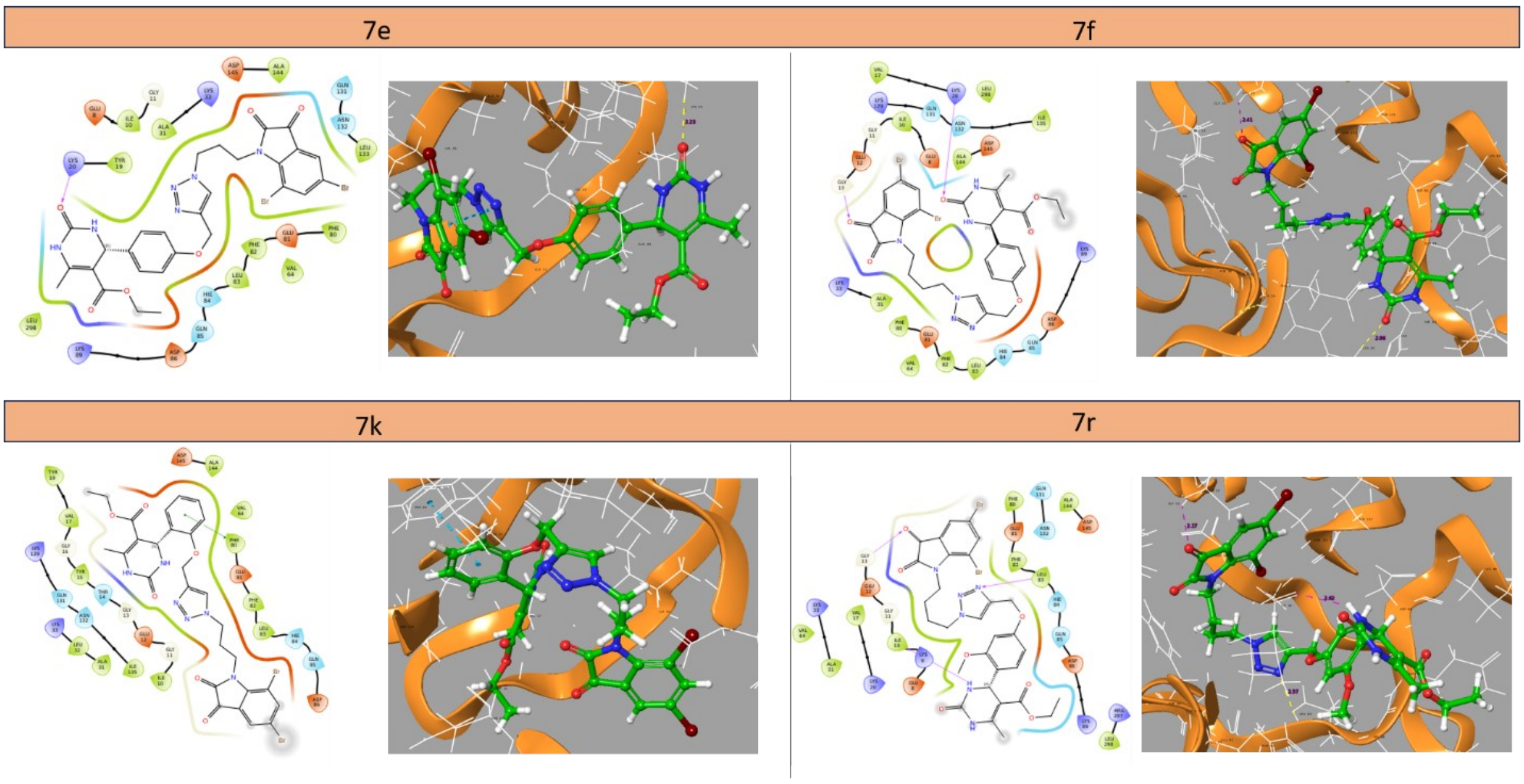

Figure 1. 2D and 3D interactions of CDK2 protein with compounds **7e**, **7f**, **7k** and **7r**, respectively.

Table 1. Docking calculations of compounds **7e**, **7f**, **7k** and **7r.**

Compounds	XP GScore	Docking Score	Glide Score	Glide emodel
7e	-4.115	-4.115	-4.115	-89.668
7f	-5.172	-5.172	-5.172	-108.222
7k	-5.560	-5.560	-5.560	-89.668
7r	-5.900	-5.900	-5.900	-10.575
Sunitinib	-6.537	-6.537	-6.537	-64.053

2. Molecular docking protocols

The molecular docking was performed using Schrodinger (Schrödinger Release 2022-1: Maestro, Schrödinger, LLC, New York, NY). Crystal co-ordinates of CDK2 protein (PDB ID: 2BHE) were downloaded from the protein data bank (https:// www.rcsb.org). In the first step, bond orders were assigned, and hydrogens were added by the pre-process option. All water molecules were deleted. The heteroatoms are ionized by epic at biological pH to consider the protein permeability and drug solubility and then the H-bonds were optimized to reduce the steric clashes by histidine, aspartate, glutamate, and hydroxyl containing amino acids. The complete protein structure was minimized by using OPLS 2005 force field. Ligand preparation is generally required because molecules lack 3D coordinates, ionization, stereochemistry, and tautomers. Thus, before docking, the least energy state of the ligand was needed to be prepared.

Ligprep tool of Schrödinger was used to prepare the least energy state of the ligand which converts 1D/2D structures to 3D. Finally, this ligand molecule was minimized with the help of the OPLS 2005 force field. For docking, the grids were generated by using the grid-based energy descriptor which had a default set of options with van der Waals radius of 1.0. The glide docking of the molecules was carried out using the previously prepared receptor grid and the ligand molecules. The favourable interactions between ligand molecules and the receptor were scored using the Glide ligand docking program. All the docking calculations were performed using extra precision (XP) mode and the OPLS-2005 force field. Thus, H-bonding, hydrophobic interactions, and pi–pi stacking between the enzyme and compound were determined.[2-6]

3. Scanned ^{1}H and ^{13}C spectra of the synthesized compounds.

Ethyl 4-(4-((1-(2-(2,3-dioxoindolin-1-yl)ethyl)-1H-1,2,3-triazol-4-yl)methoxy)phenyl)-6-methyl-2-oxo-1,2,3,4-tetrahydropyrimidine-5-carboxylate (7a):

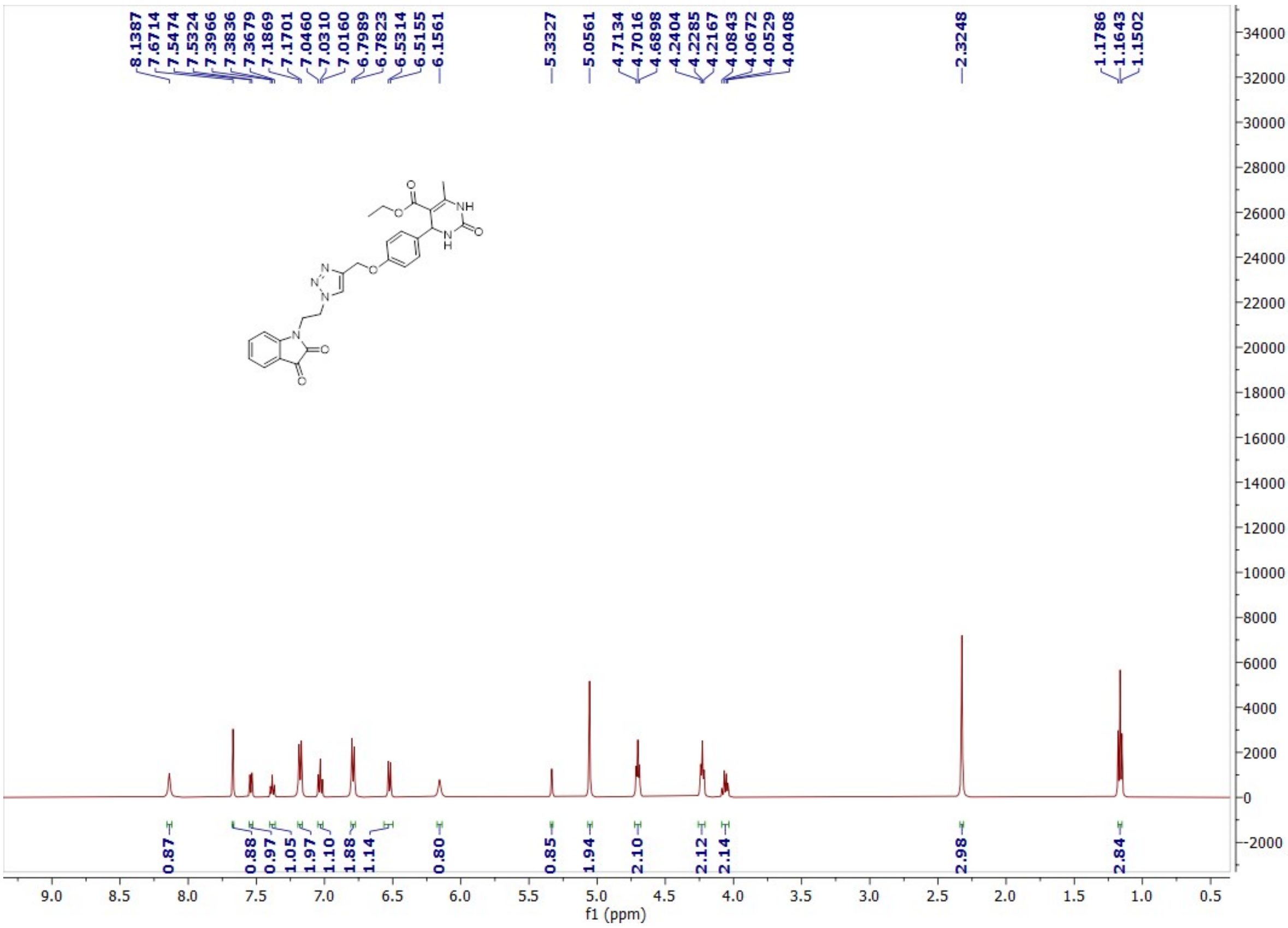

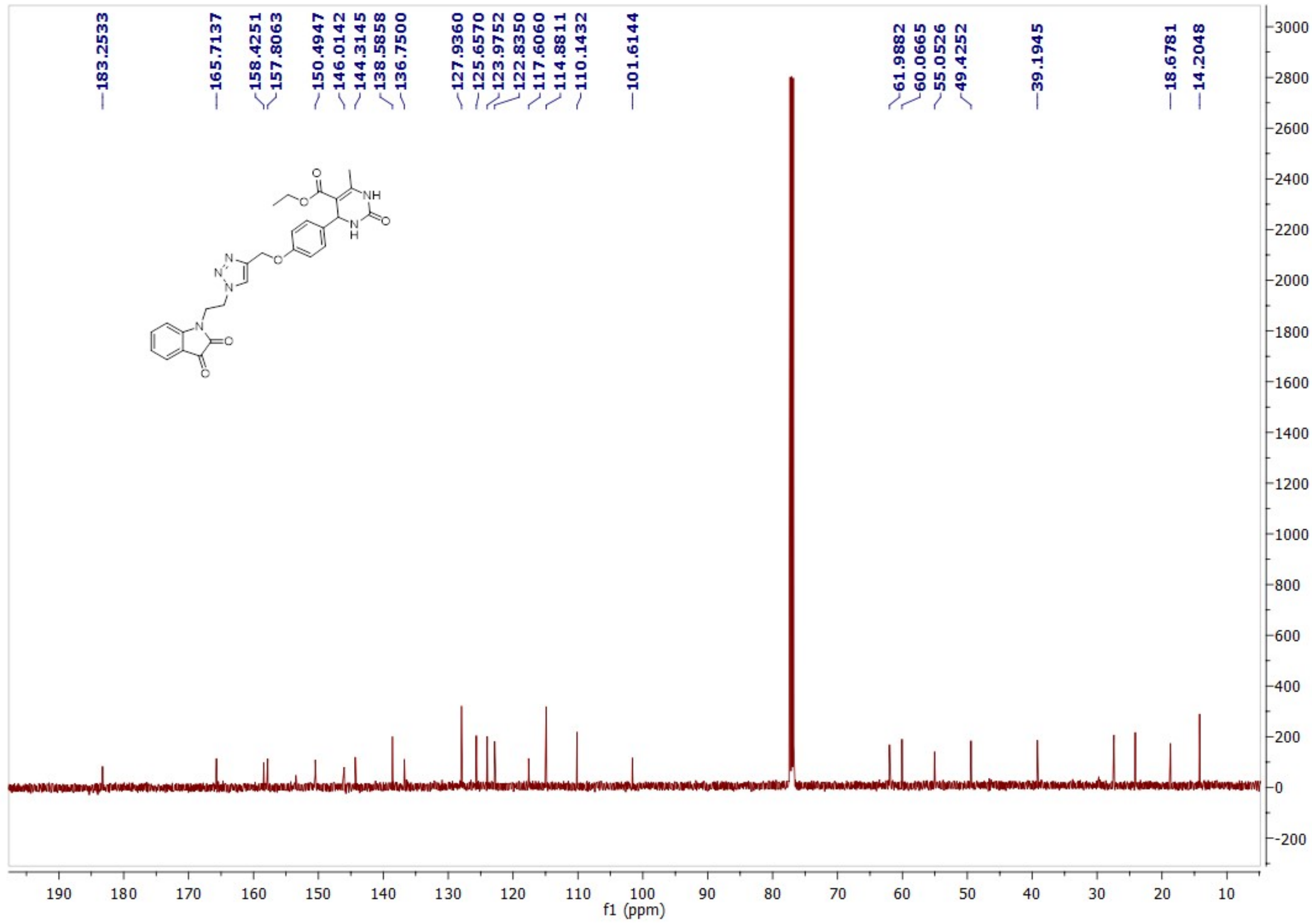

Ethyl 4-(4-((1-(3-(2,3-dioxoindolin-1-yl)propyl)-1H-1,2,3-triazol-4-yl)methoxy)phenyl)-6-methyl-2-oxo-1,2,3,4-tetrahydropyrimidine-5-carboxylate (7b):

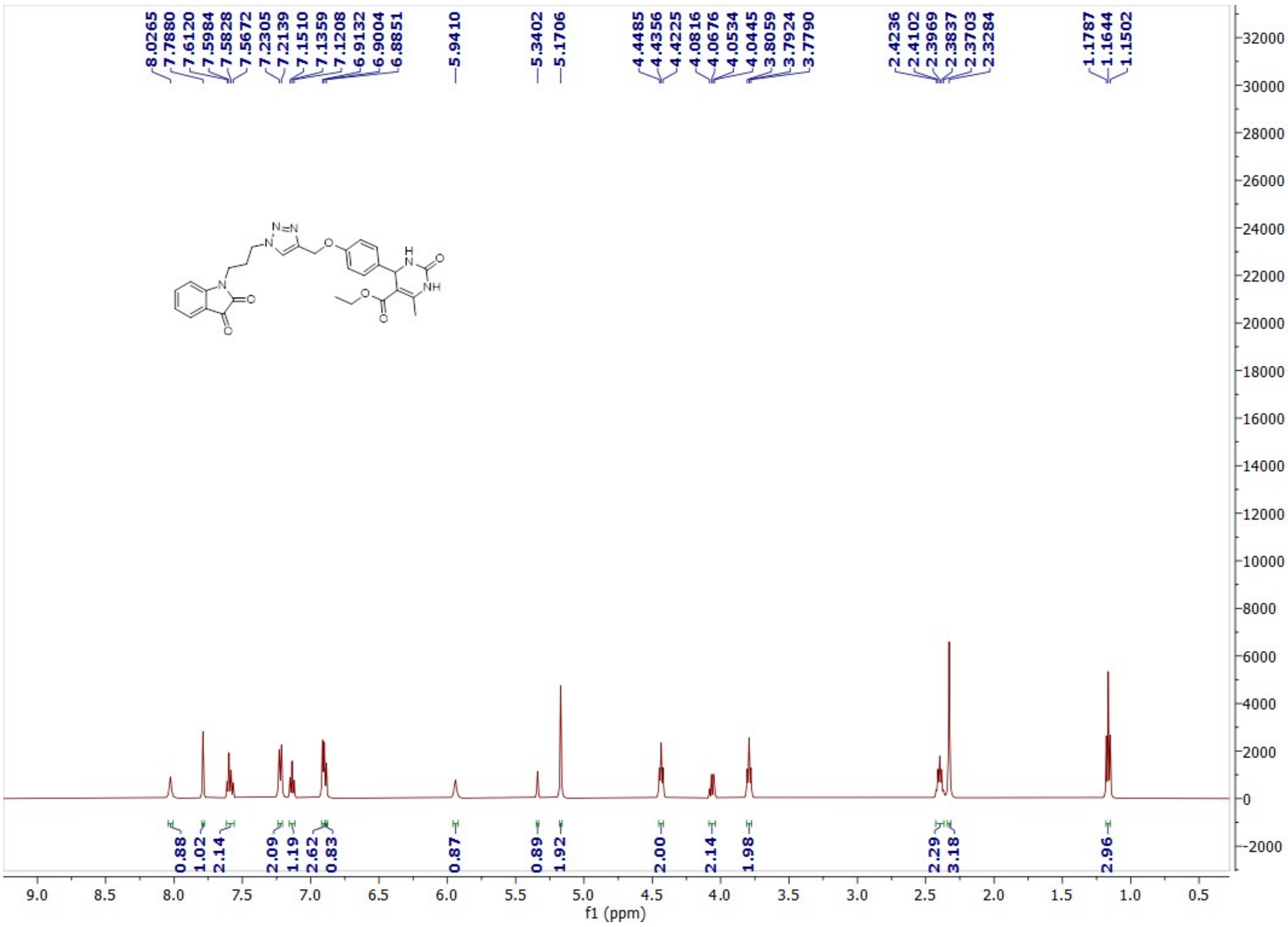
8.0265
7.7880
7.6120
7.5984
7.5828
7.5672
7.2305
7.2139
7.1510
7.1359
7.1208
6.9132
6.9004
6.8851
5.9410
5.3402
5.1706
4.4485
4.4356
4.4225
4.0816
4.0676
4.0534
4.0445
3.8059
3.7924
3.7790
2.4236
2.4102
2.3969
2.3837
2.3703
2.3284
1.1787
1.1644
1.1502
0.88
1.02
2.14
2.09
1.19
2.62
0.83
0.87
0.89
1.92
2.00
2.14
1.98
2.29
3.18
2.96
f1 (ppm)

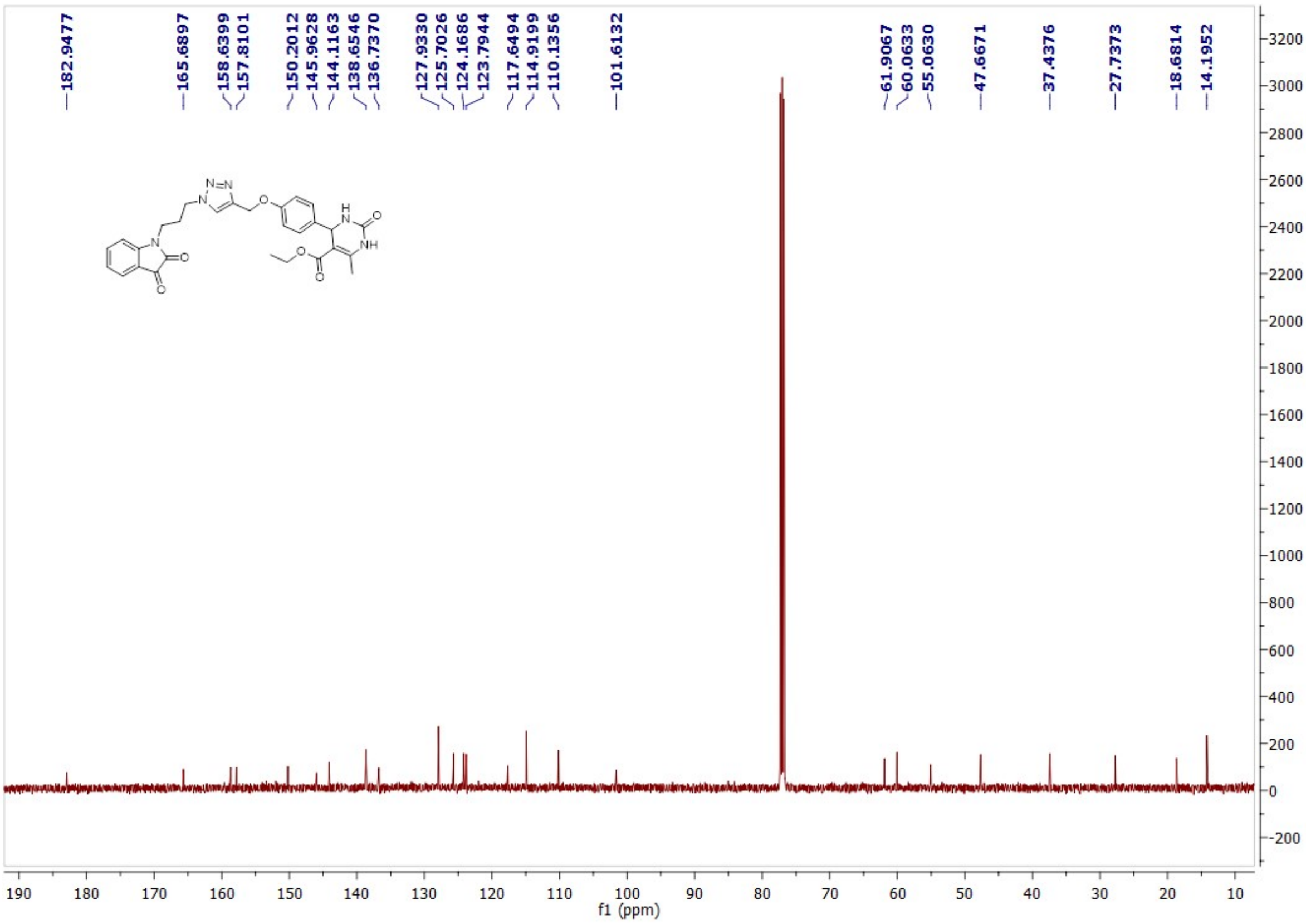
182.9477
165.6897
158.6399
157.8101
150.2012
145.9628
144.1163
138.6546
136.7370
127.9330
125.7026
124.1686
123.7944
117.6494
114.9199
110.1356
101.6132
61.9067
60.0633
55.0630
47.6671
37.4376
27.7373
18.6814
14.1952
f1 (ppm)

Ethyl 4-(4-((1-(4-(2,3-dioxoindolin-1-yl)butyl)-1H-1,2,3-triazol-4-yl)methoxy)phenyl)-6-methyl-2-oxo-1,2,3,4-tetrahydropyrimidine-5-carboxylate (7c):

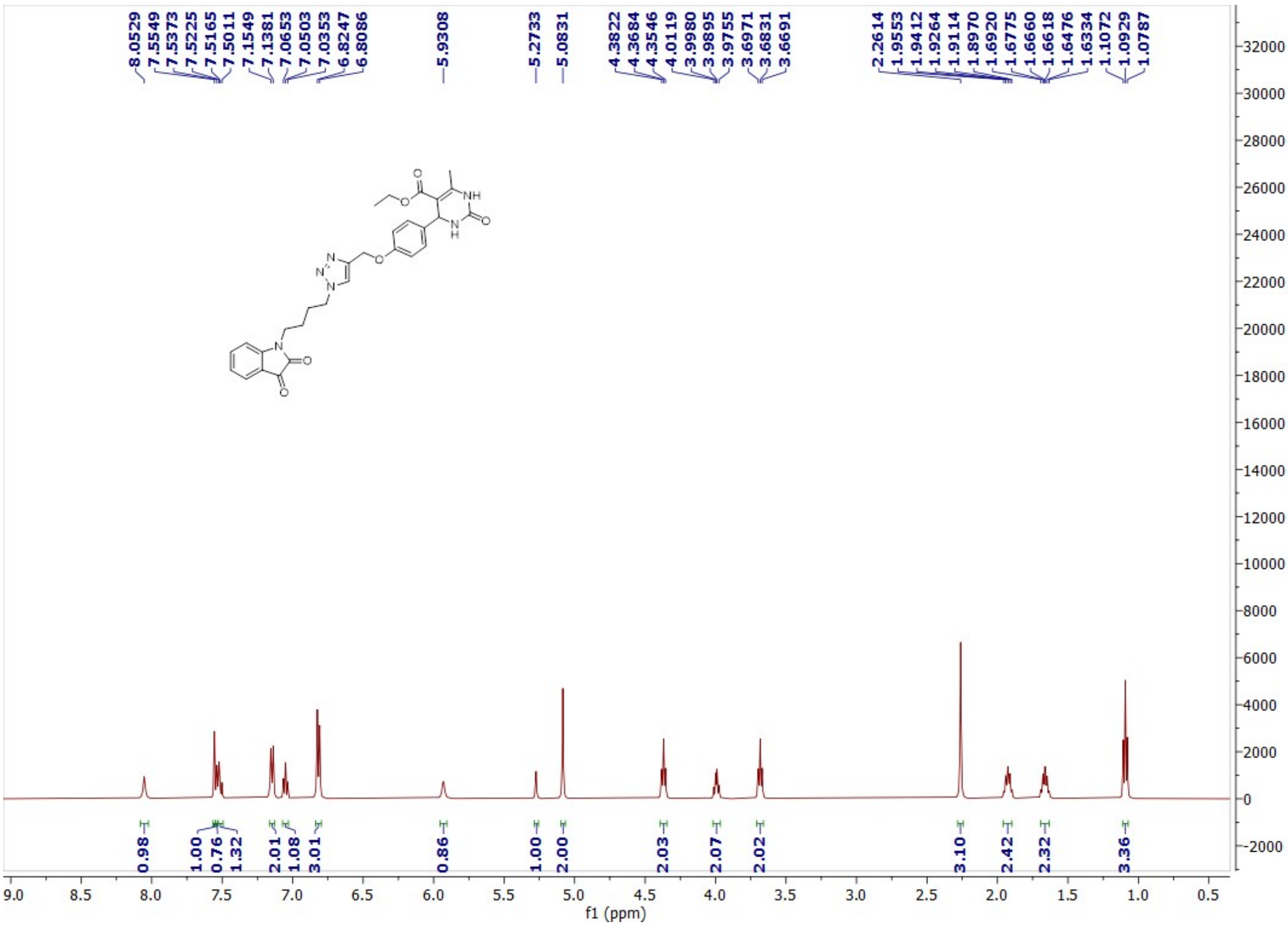

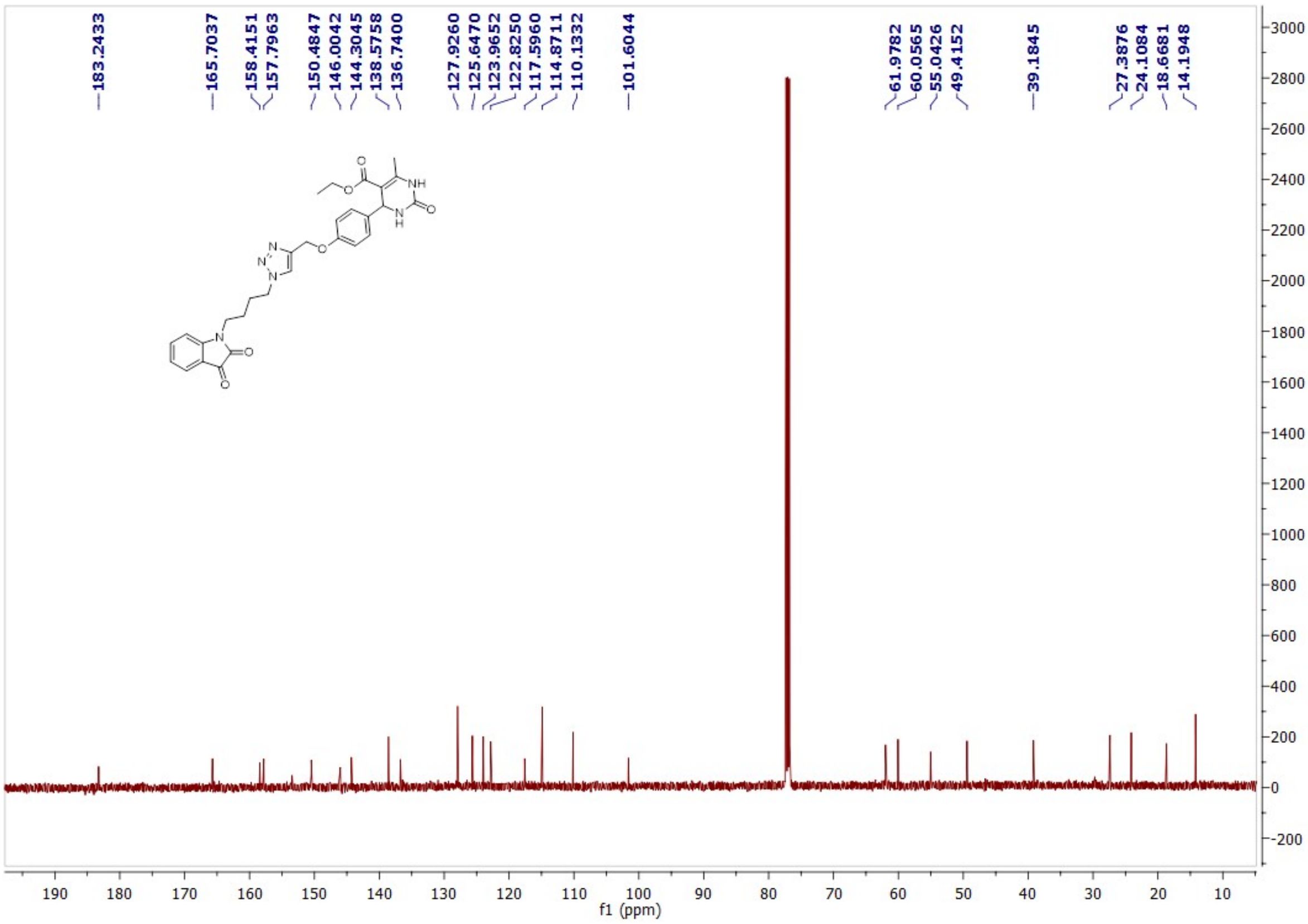

Ethyl 4-(4-((1-(2-(5,7-dibromo-2,3-dioxoindolin-1-yl)ethyl)-1H-1,2,3-triazol-4-yl)methoxy)phenyl)-6-methyl-2-oxo-1,2,3,4-tetrahydropyrimidine-5-carboxylate (7d):

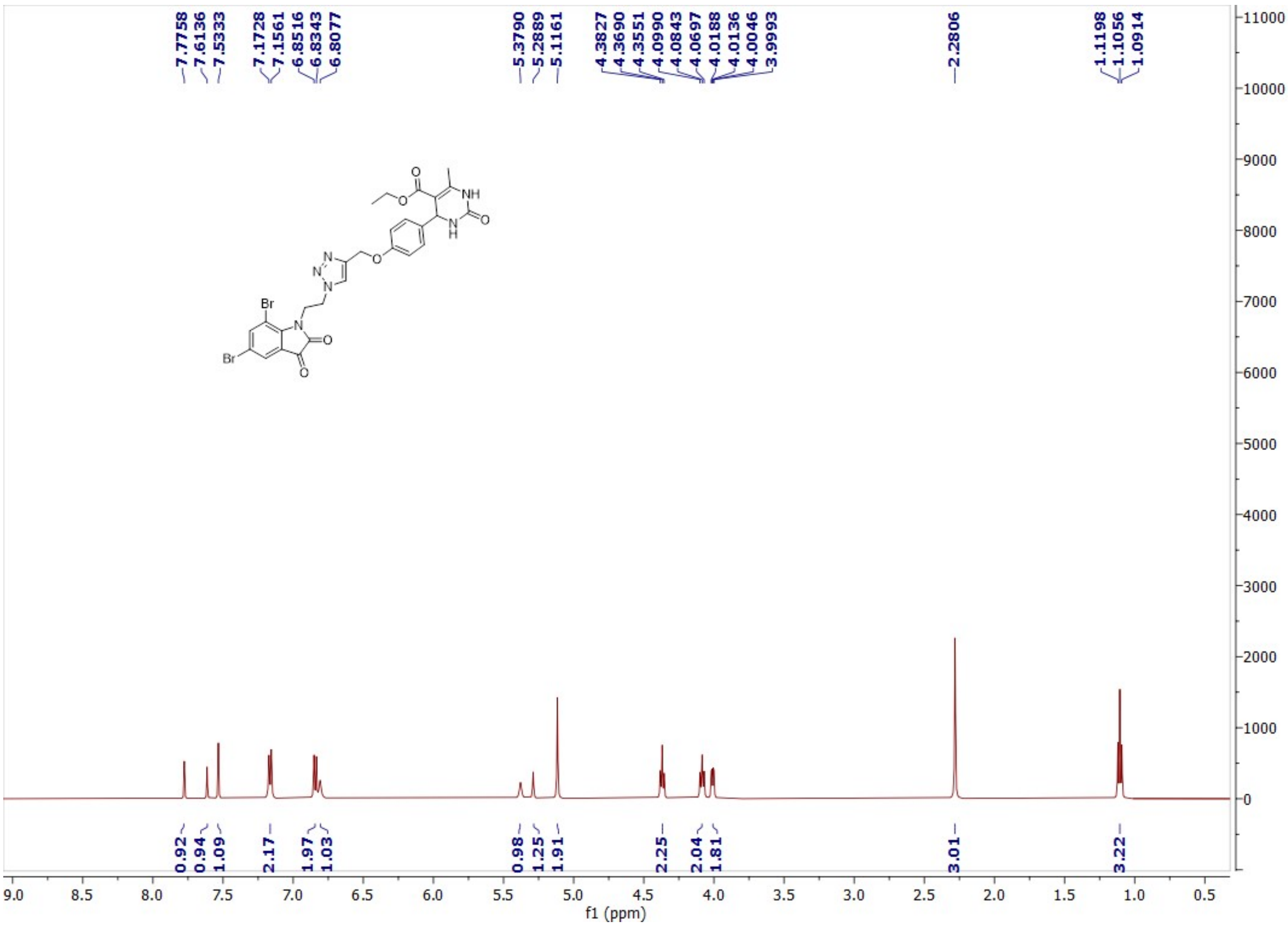

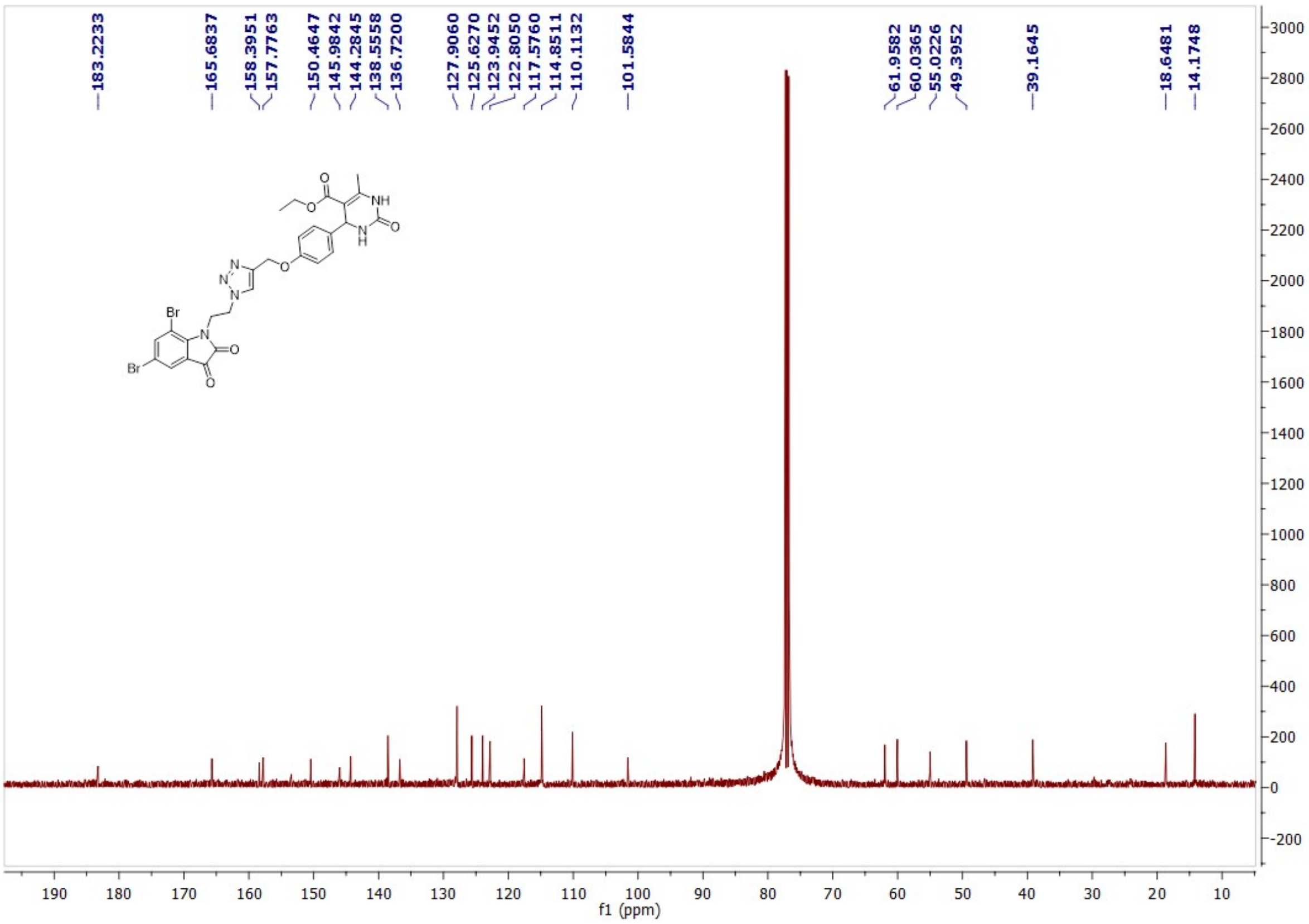

Ethyl 4-(4-((1-(3-(5,7-dibromo-2,3-dioxoindolin-1-yl)propyl)-1H-1,2,3-triazol-4-yl)methoxy)phenyl)-6-methyl-2-oxo-1,2,3,4-tetrahydropyrimidine-5-carboxylate (7e):

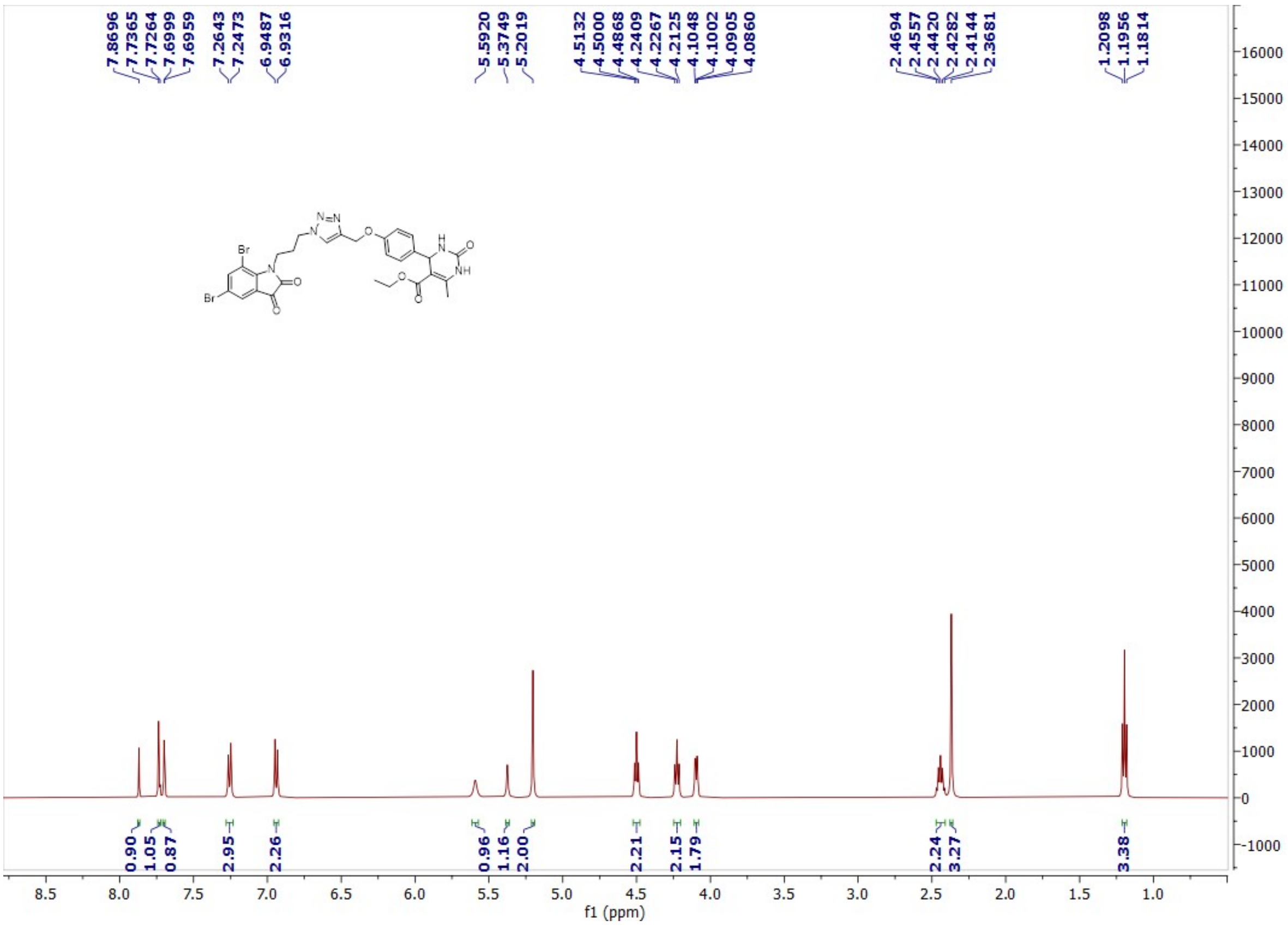

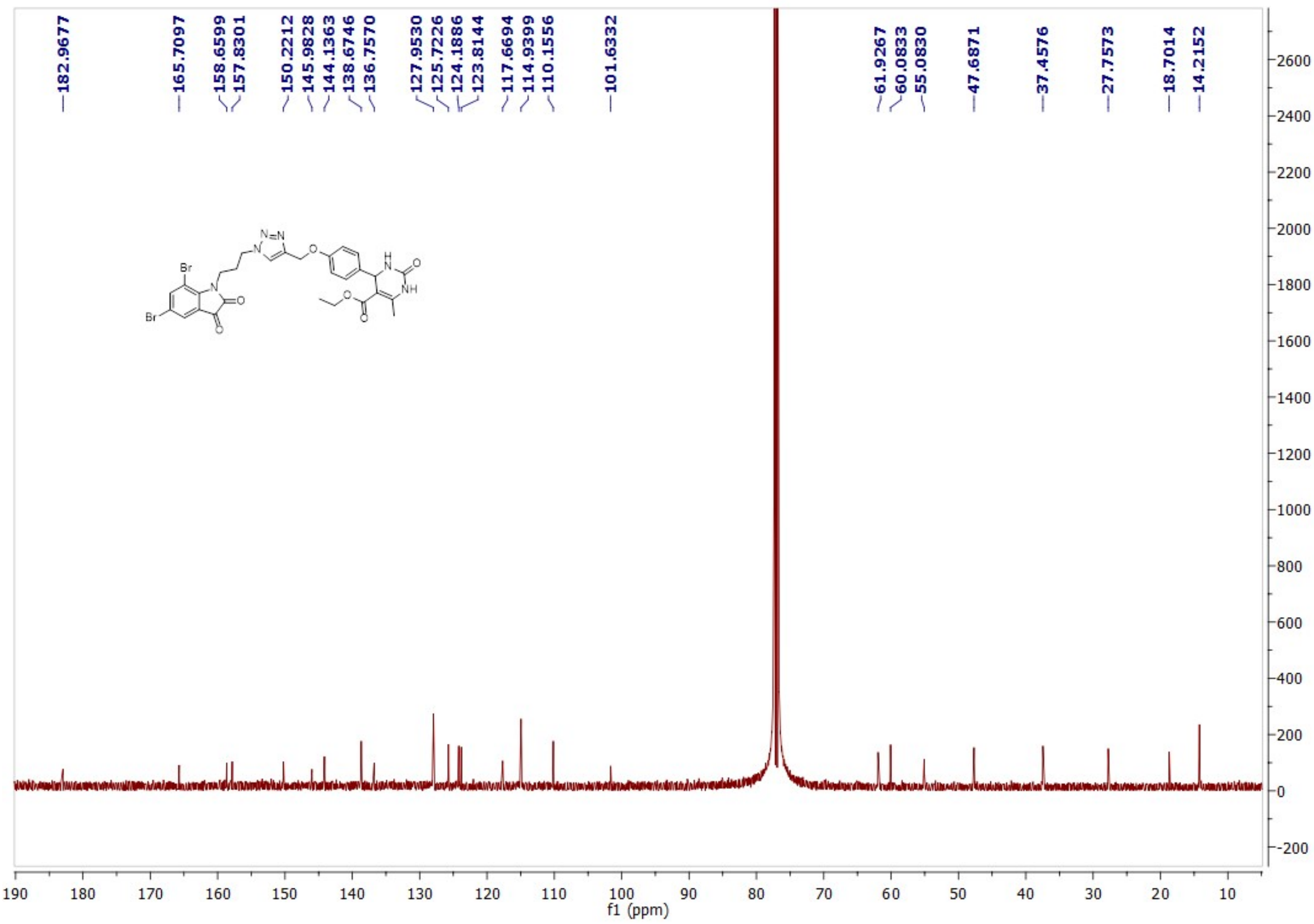

Ethyl 4-(4-((1-(4-(5,7-dibromo-2,3-dioxoindolin-1-yl)butyl)-1H-1,2,3-triazol-4-yl)methoxy)phenyl)-6-methyl-2-oxo-1,2,3,4-tetrahydropyrimidine-5-carboxylate (7f):

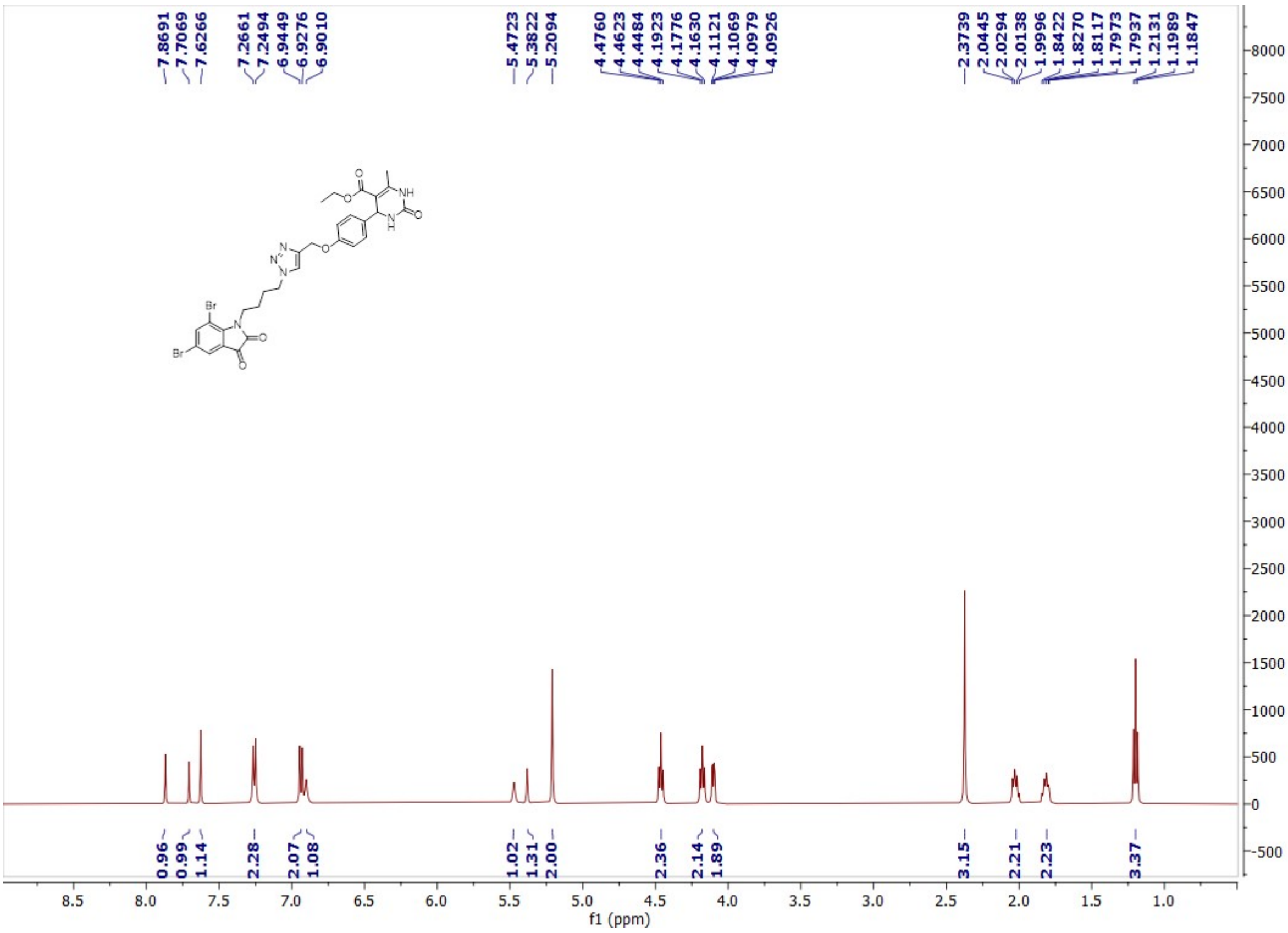

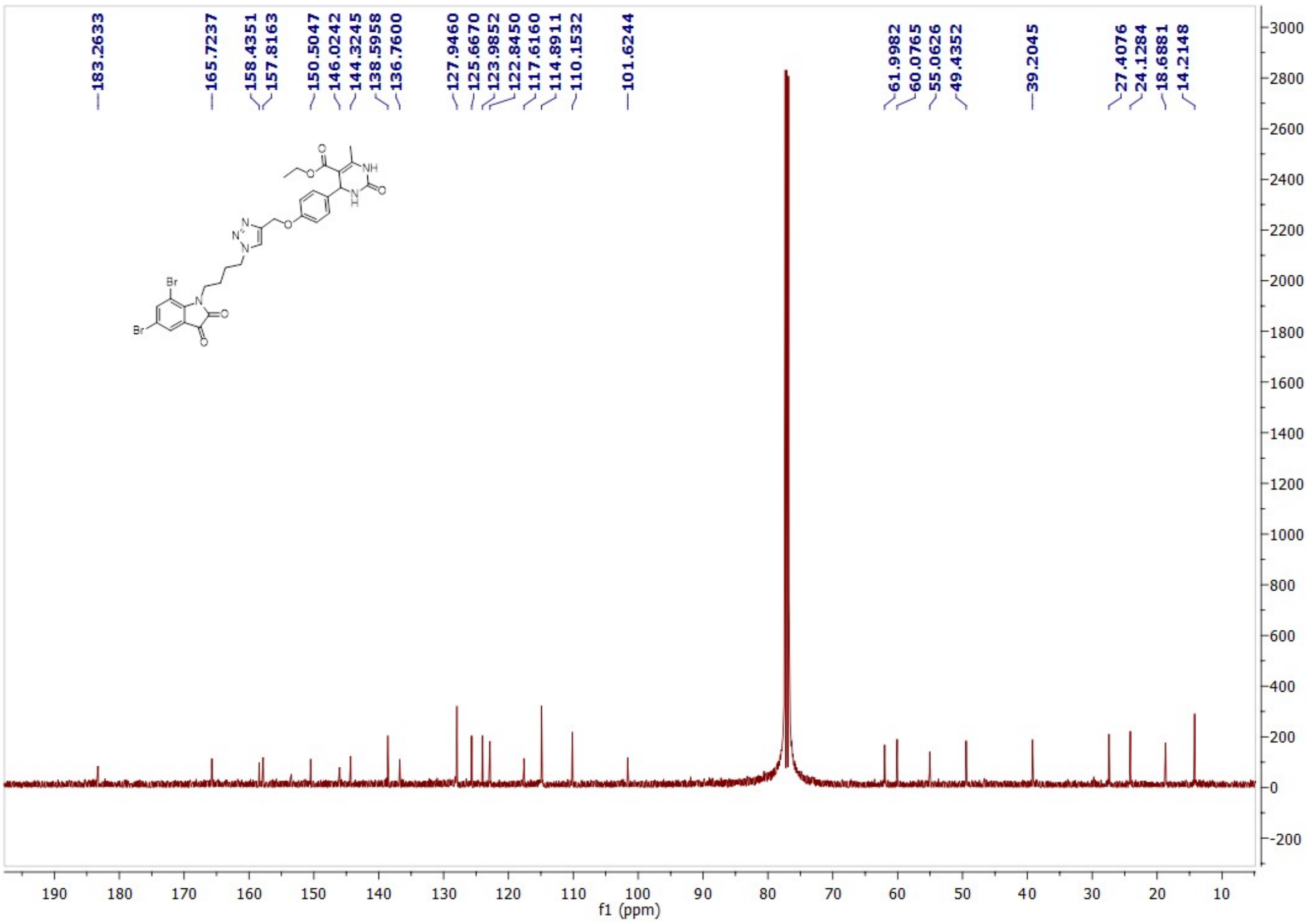

Ethyl 4-(2-((1-(2-(2,3-dioxoindolin-1-yl)ethyl)-1H-1,2,3-triazol-4-yl)methoxy)phenyl)-6-methyl-2-oxo-1,2,3,4-tetrahydropyrimidine-5-carboxylate (7g):

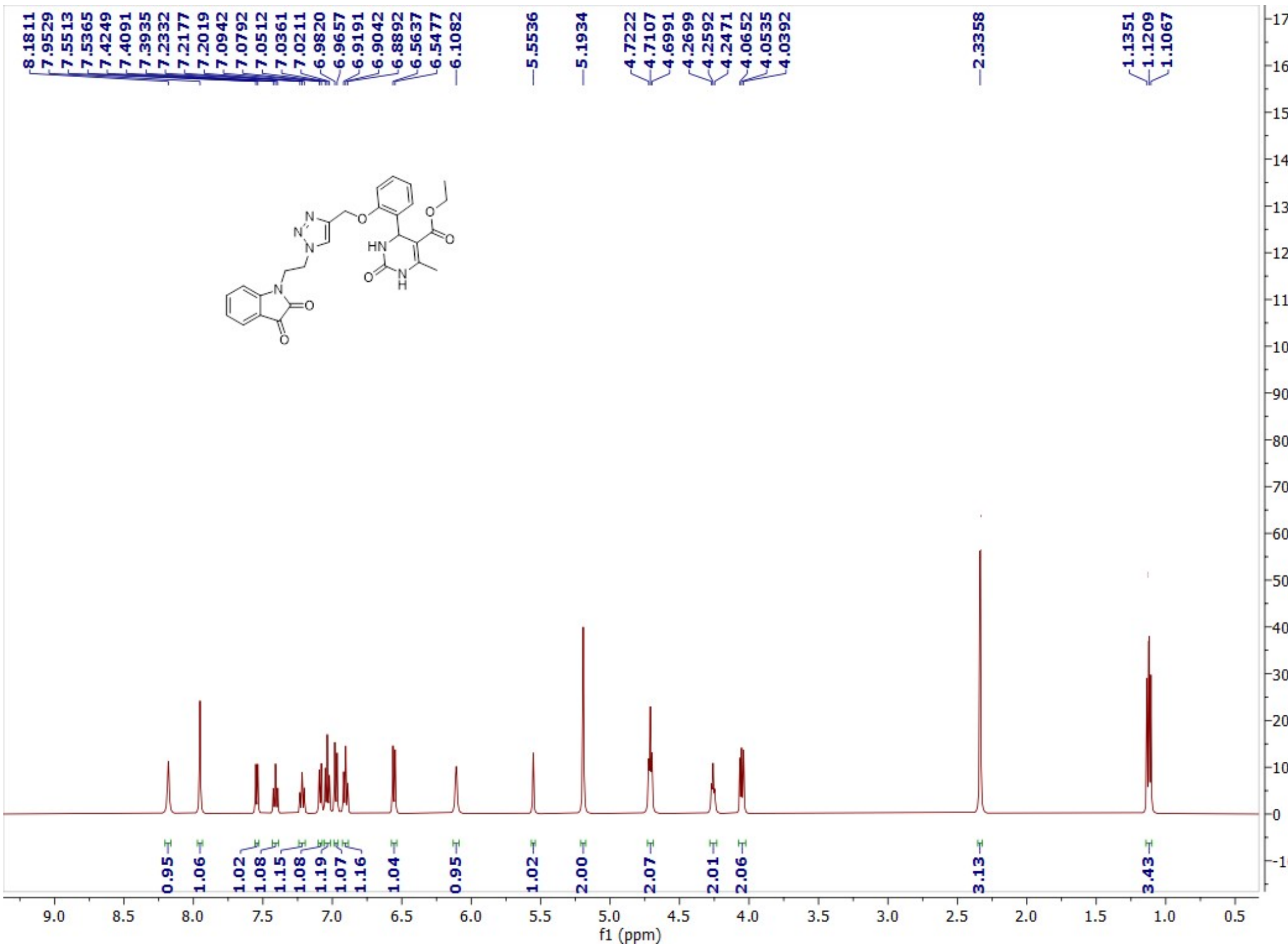

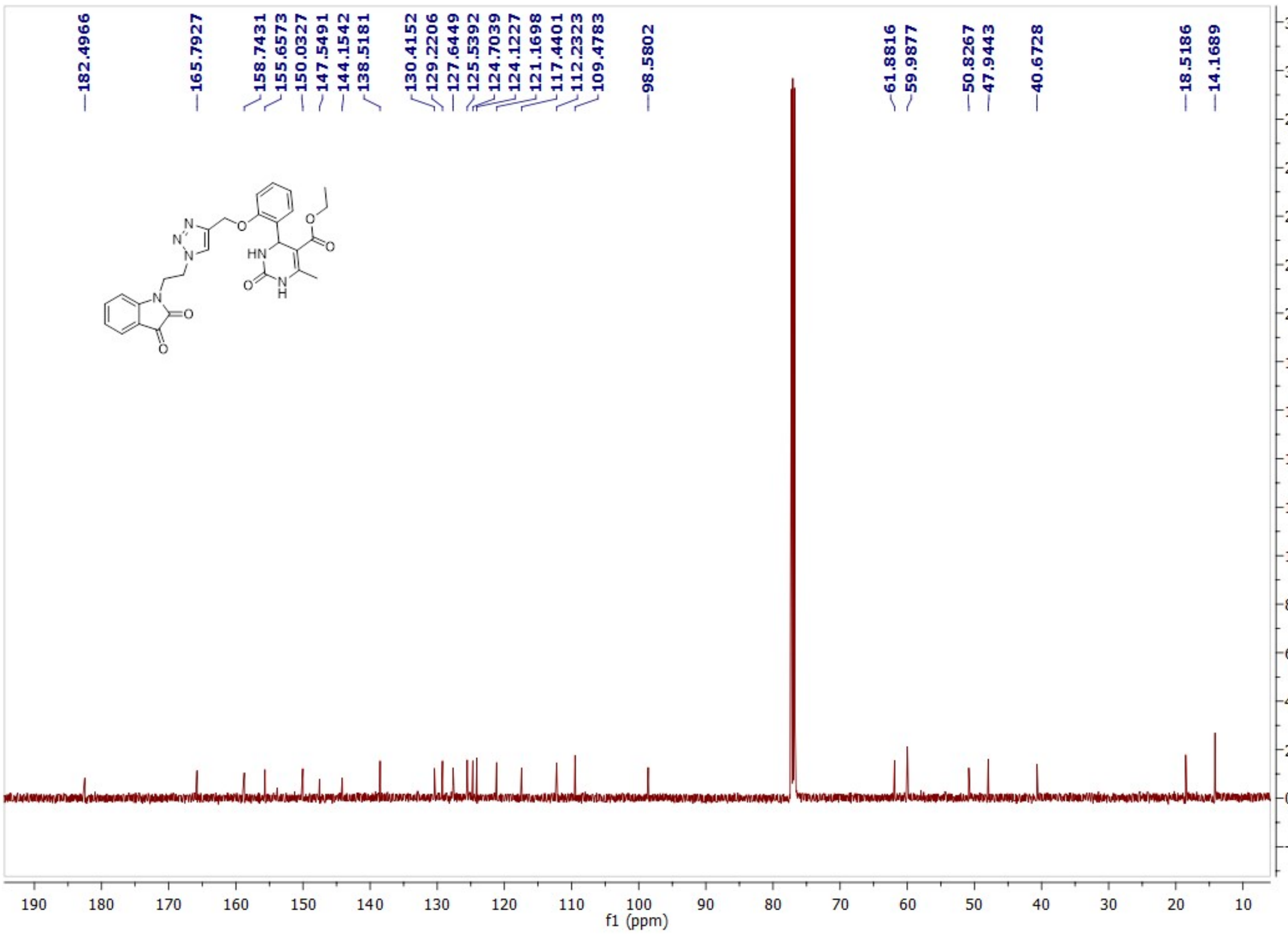

Ethyl 4-(2-((1-(3-(2,3-dioxoindolin-1-yl)propyl)-1H-1,2,3-triazol-4-yl)methoxy)phenyl)-6-methyl-2-oxo-1,2,3,4-tetrahydropyrimidine-5-carboxylate (7h):

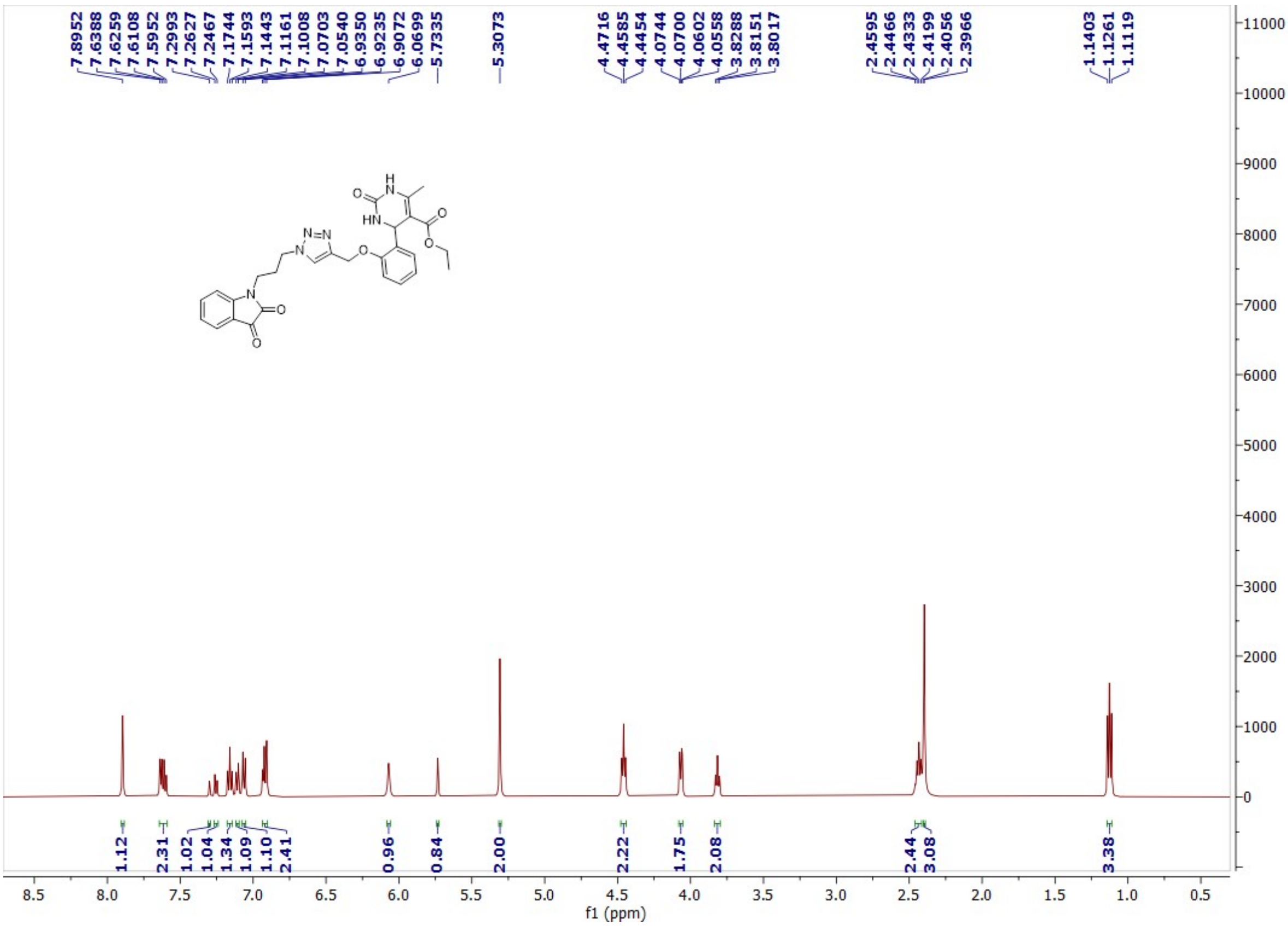

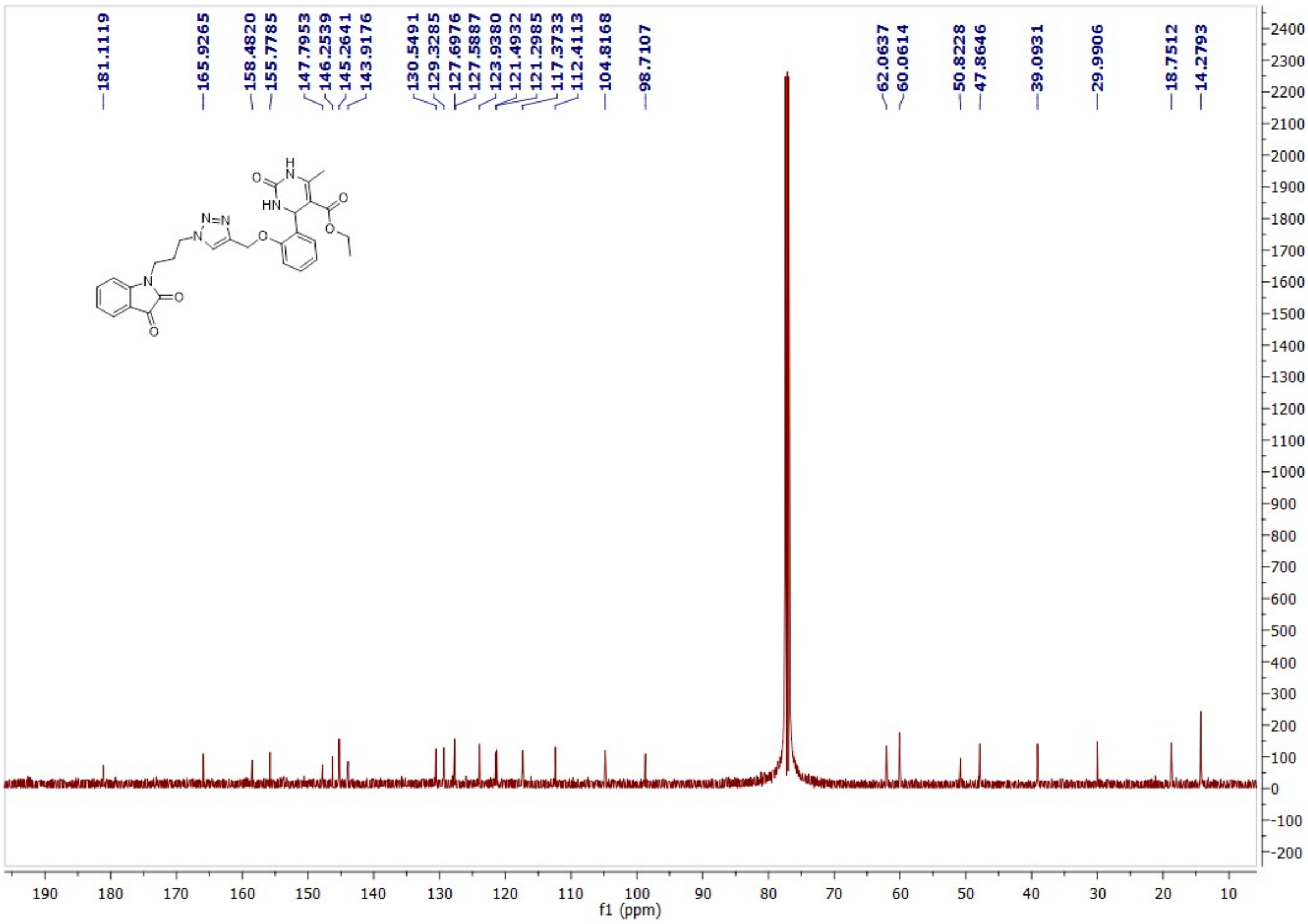

Ethyl 4-(2-((1-(4-(2,3-dioxoindolin-1-yl)butyl)-1H-1,2,3-triazol-4-yl)methoxy)phenyl)-6-methyl-2-oxo-1,2,3,4-tetrahydropyrimidine-5-carboxylate (7i):

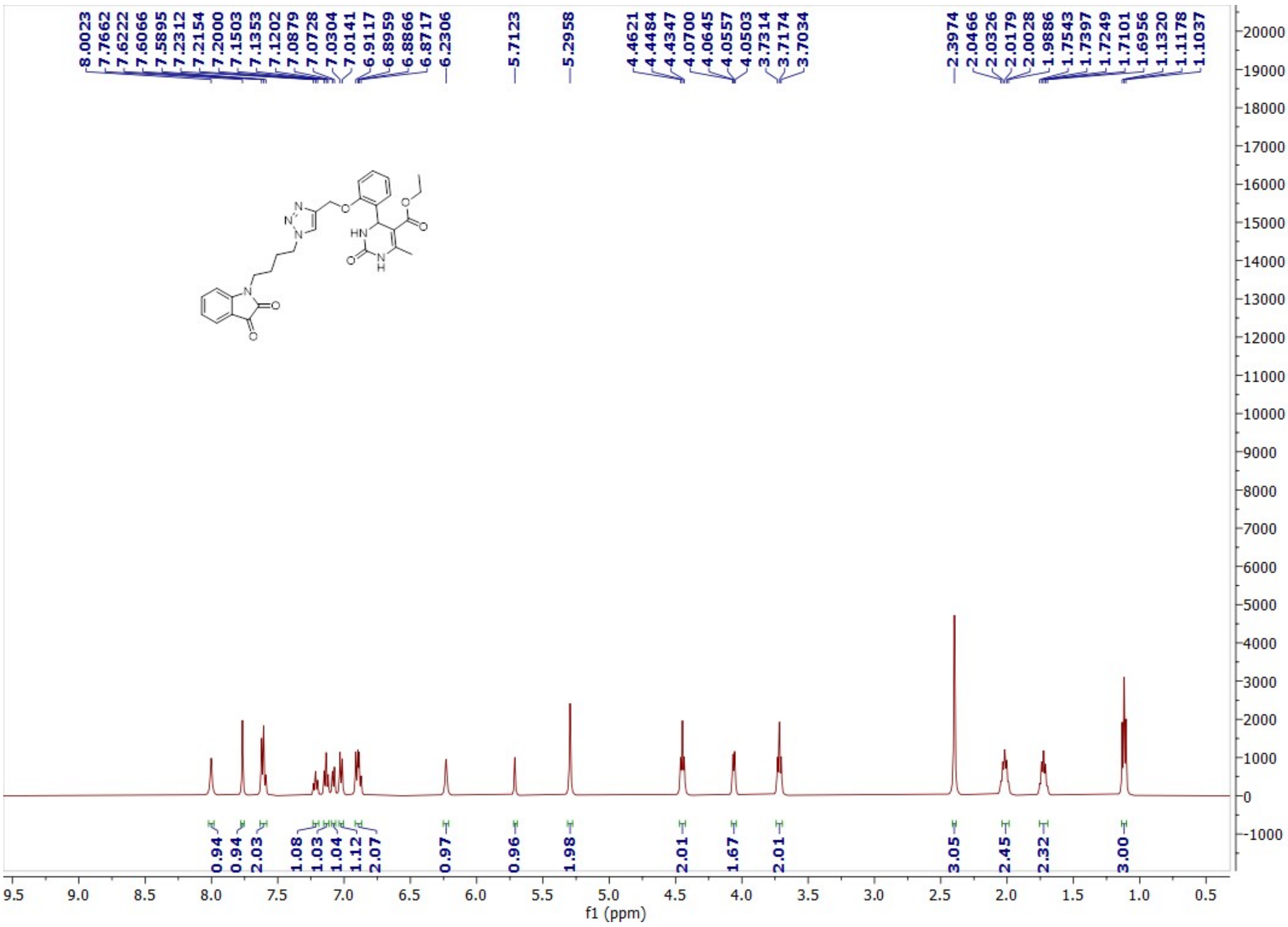

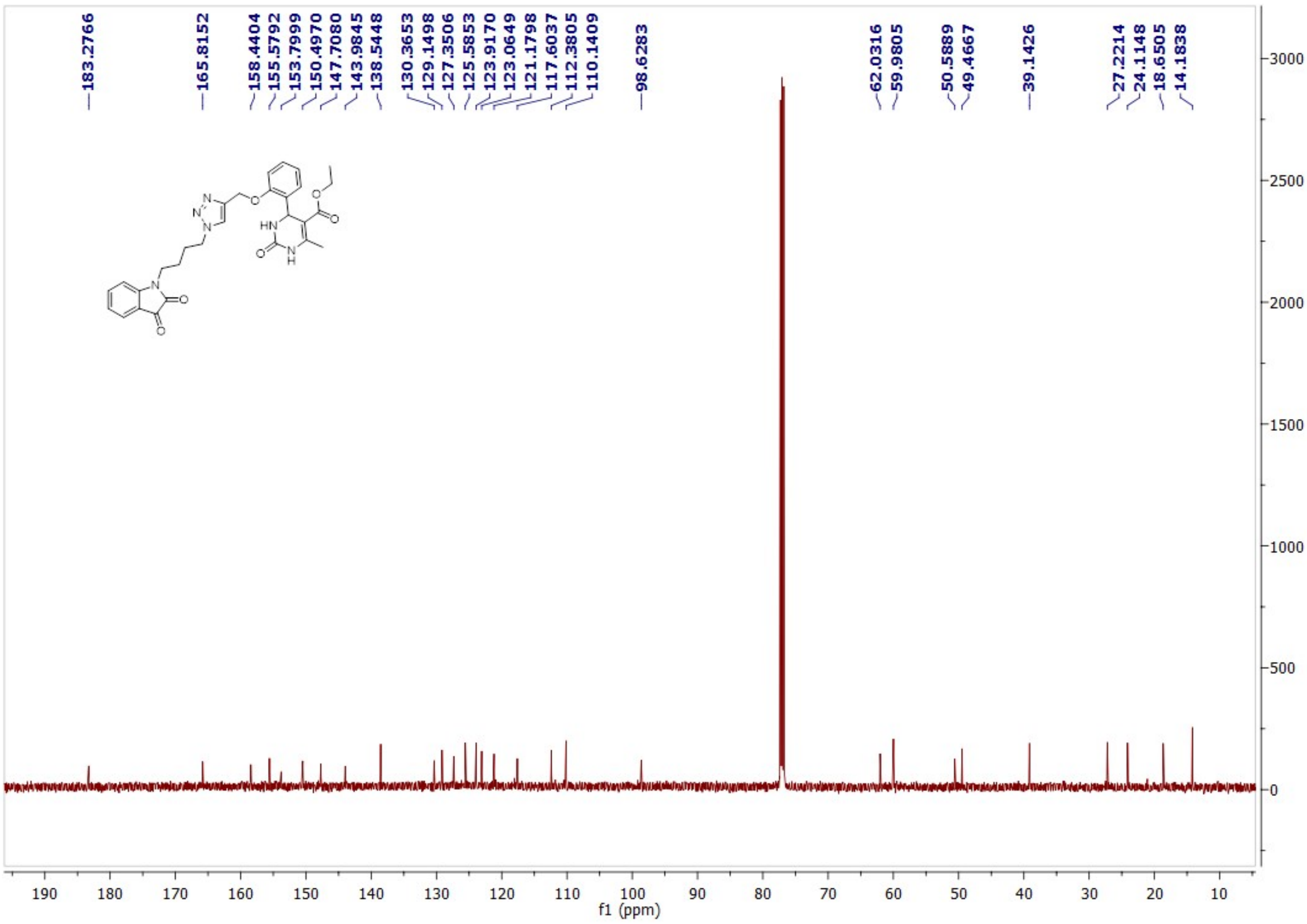

Ethyl 4-(2-((1-(2-(5,7-dibromo-2,3-dioxoindolin-1-yl)ethyl)-1H-1,2,3-triazol-4-yl)methoxy)phenyl)-6-methyl-2-oxo-1,2,3,4-tetrahydropyrimidine-5-carboxylate (7j):

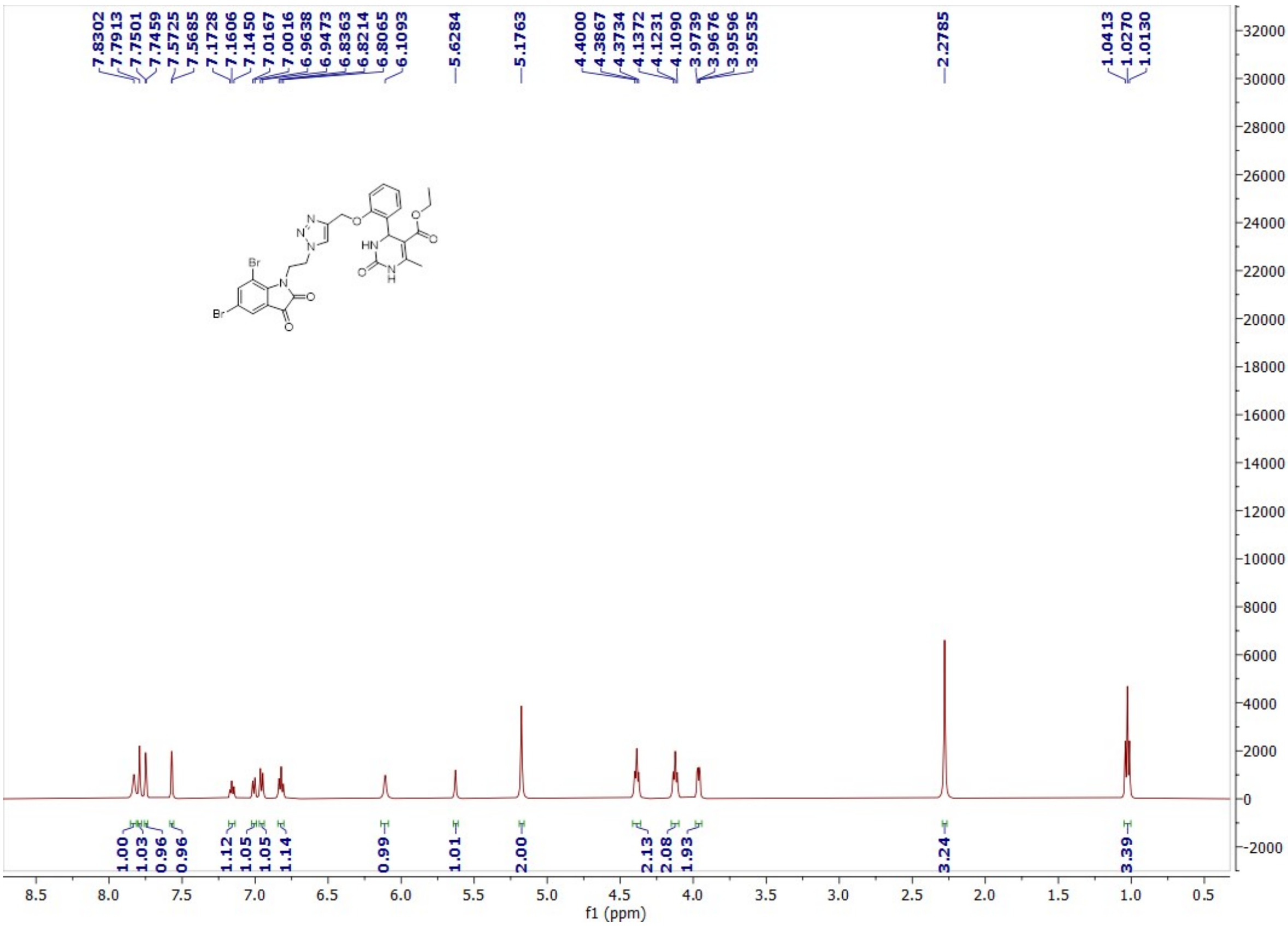

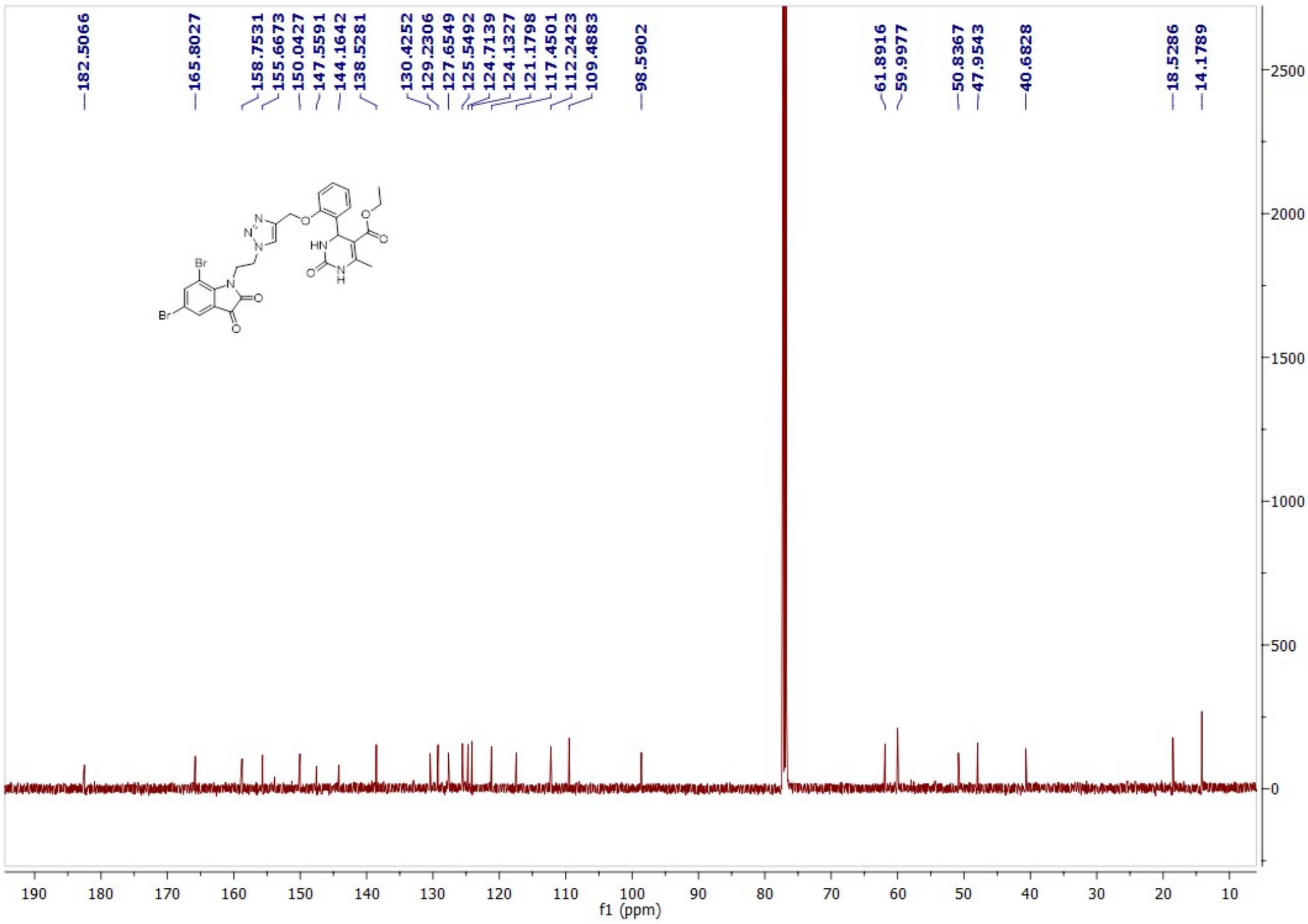

Ethyl 4-(2-((1-(3-(5,7-dibromo-2,3-dioxoindolin-1-yl)propyl)-1H-1,2,3-triazol-4-yl)methoxy)phenyl)-6-methyl-2-oxo-1,2,3,4-tetrahydropyrimidine-5-carboxylate (7k):

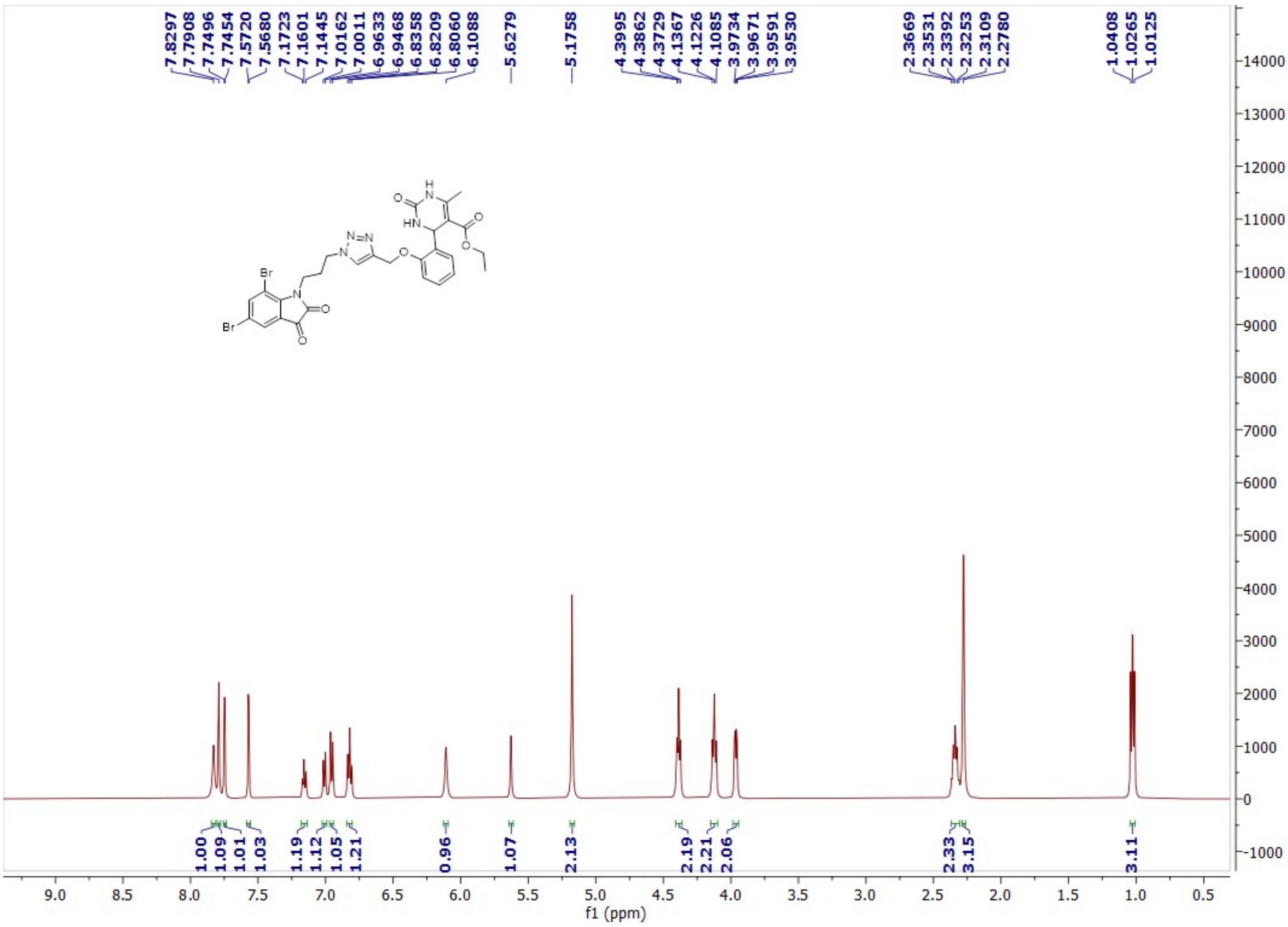

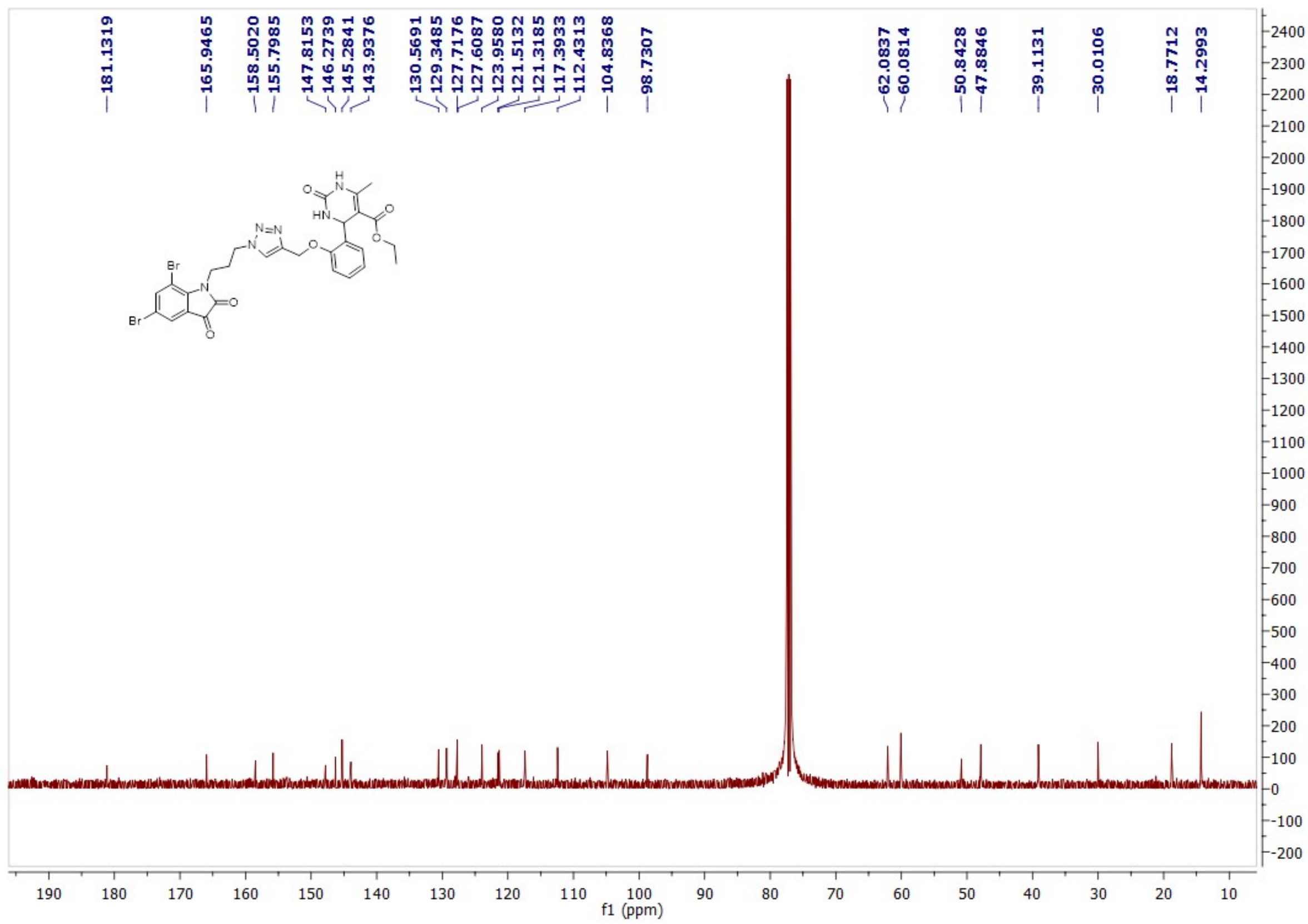

Ethyl 4-(2-((1-(4-(5,7-dibromo-2,3-dioxoindolin-1-yl)butyl)-1H-1,2,3-triazol-4-yl)methoxy)phenyl)-6-methyl-2-oxo-1,2,3,4-tetrahydropyrimidine-5-carboxylate (7l):

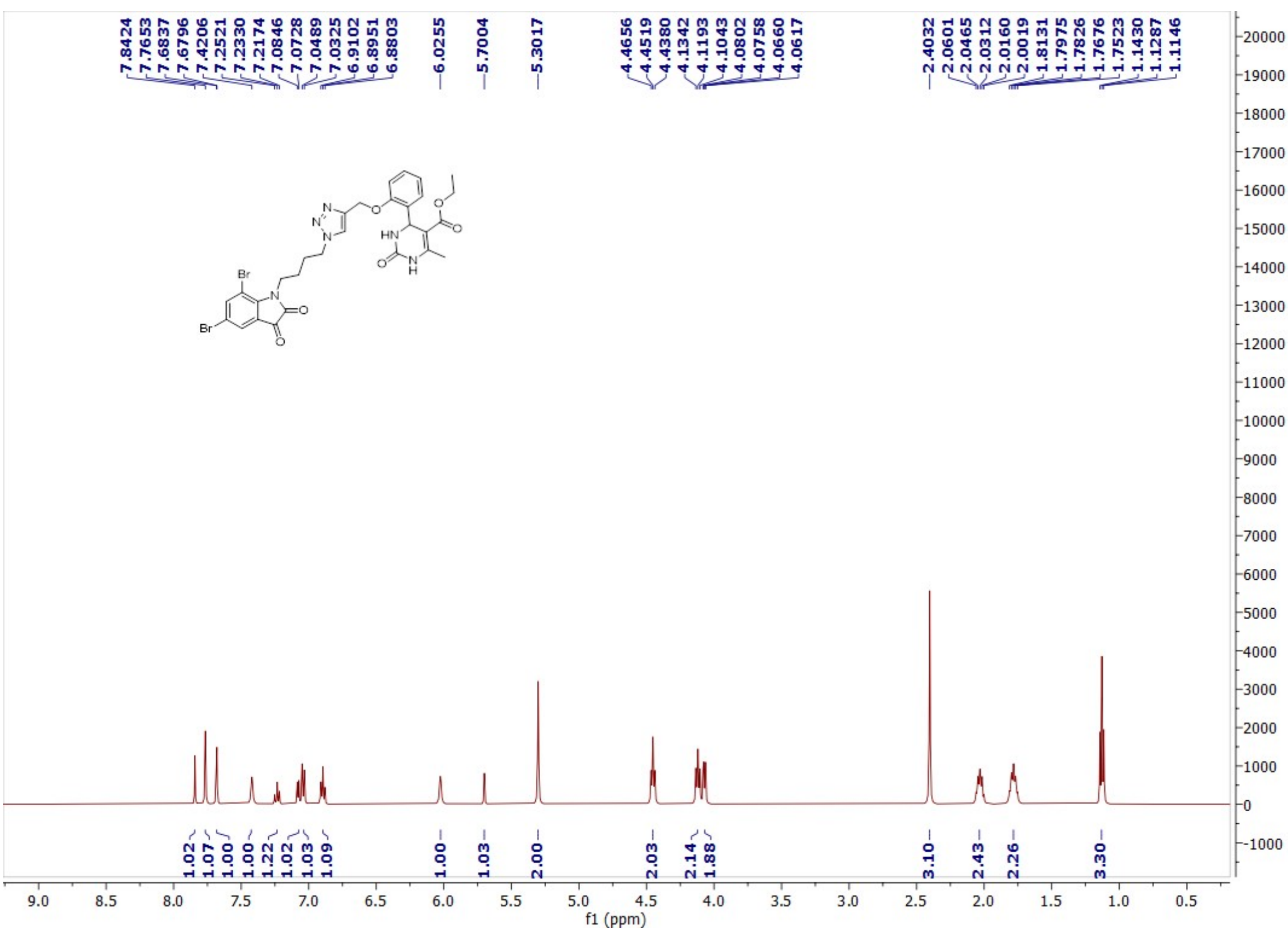

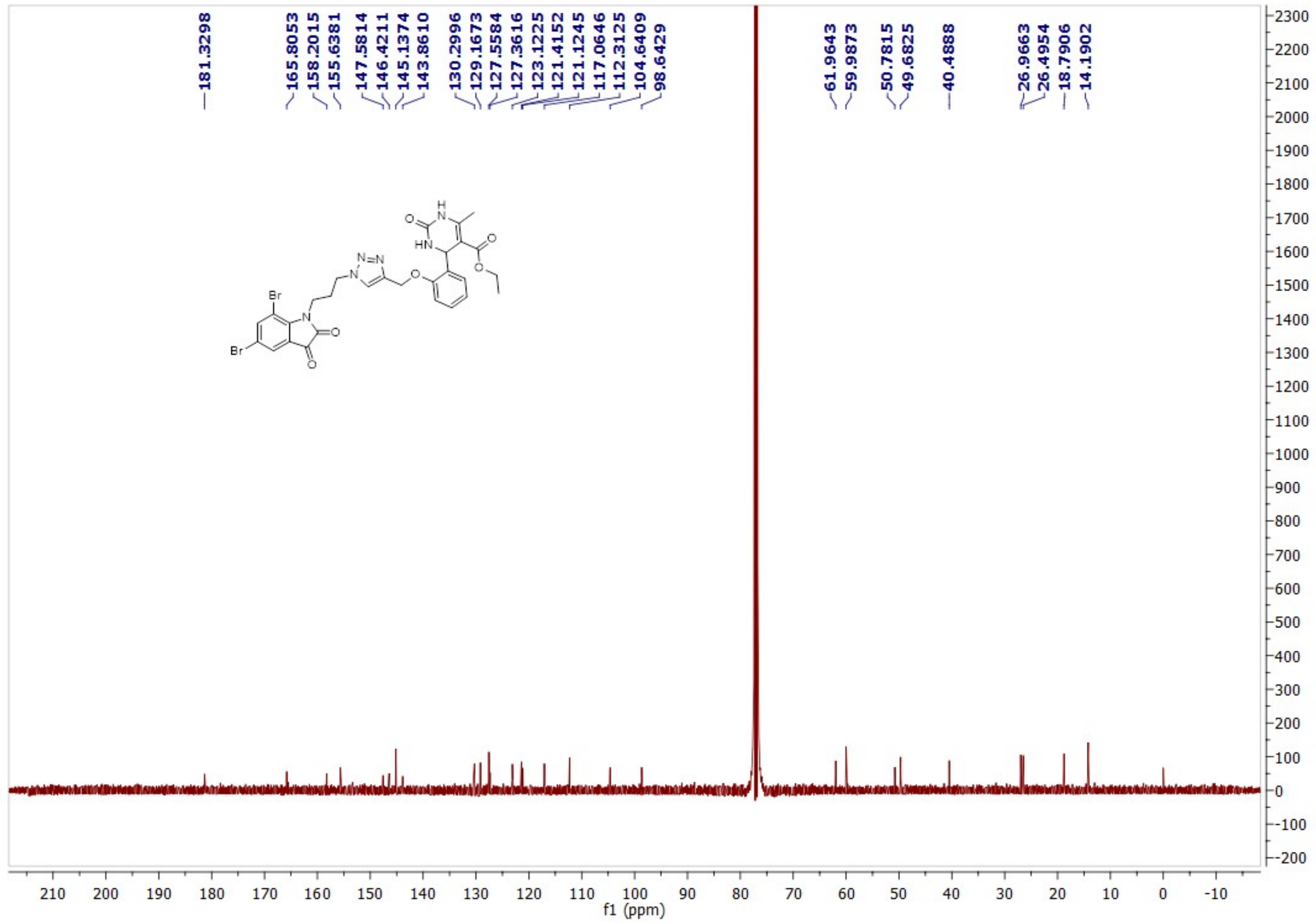

Ethyl 4-(4-((1-(2-(2,3-dioxoindolin-1-yl)ethyl)-1H-1,2,3-triazol-4-yl)methoxy)-3-methoxyphenyl)-6-methyl-2-oxo-1,2,3,4-tetrahydropyrimidine-5-carboxylate (7m):

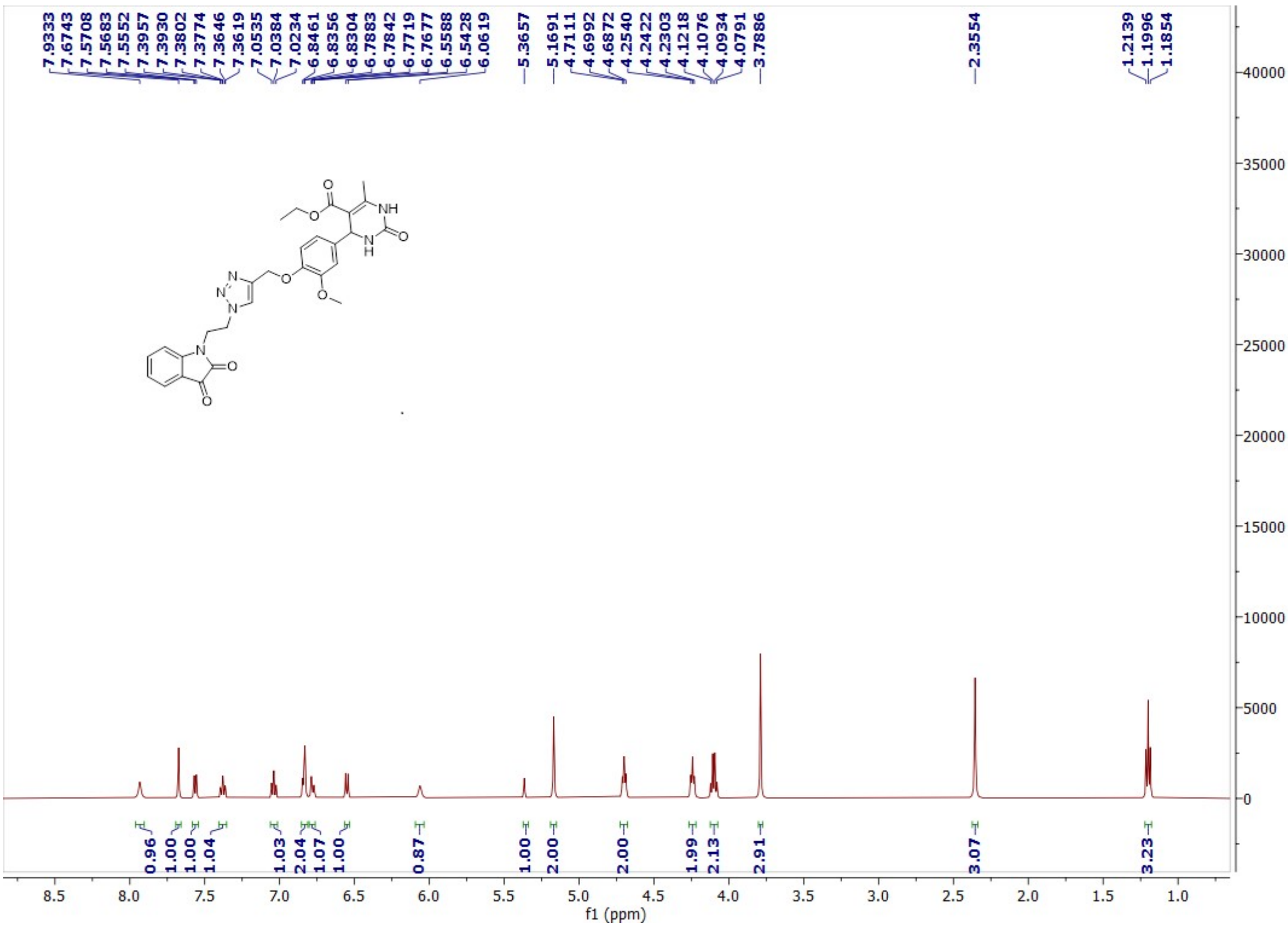

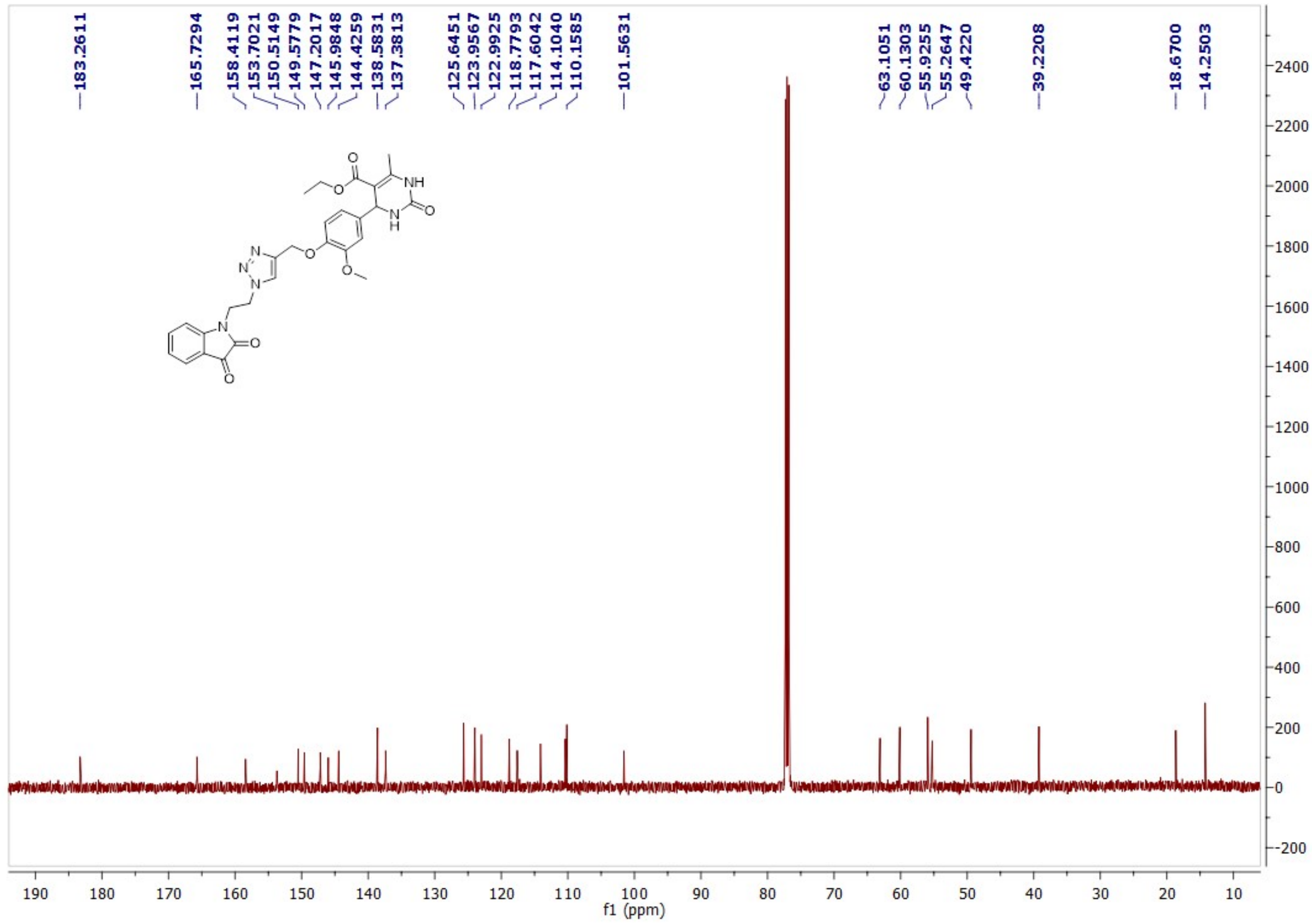

Ethyl 4-(4-((1-(3-(2,3-dioxoindolin-1-yl)propyl)-1H-1,2,3-triazol-4-yl)methoxy)-2-methoxyphenyl)-2-oxo-1,2,3,4-tetrahydropyrimidine-5-carboxylate (7n):

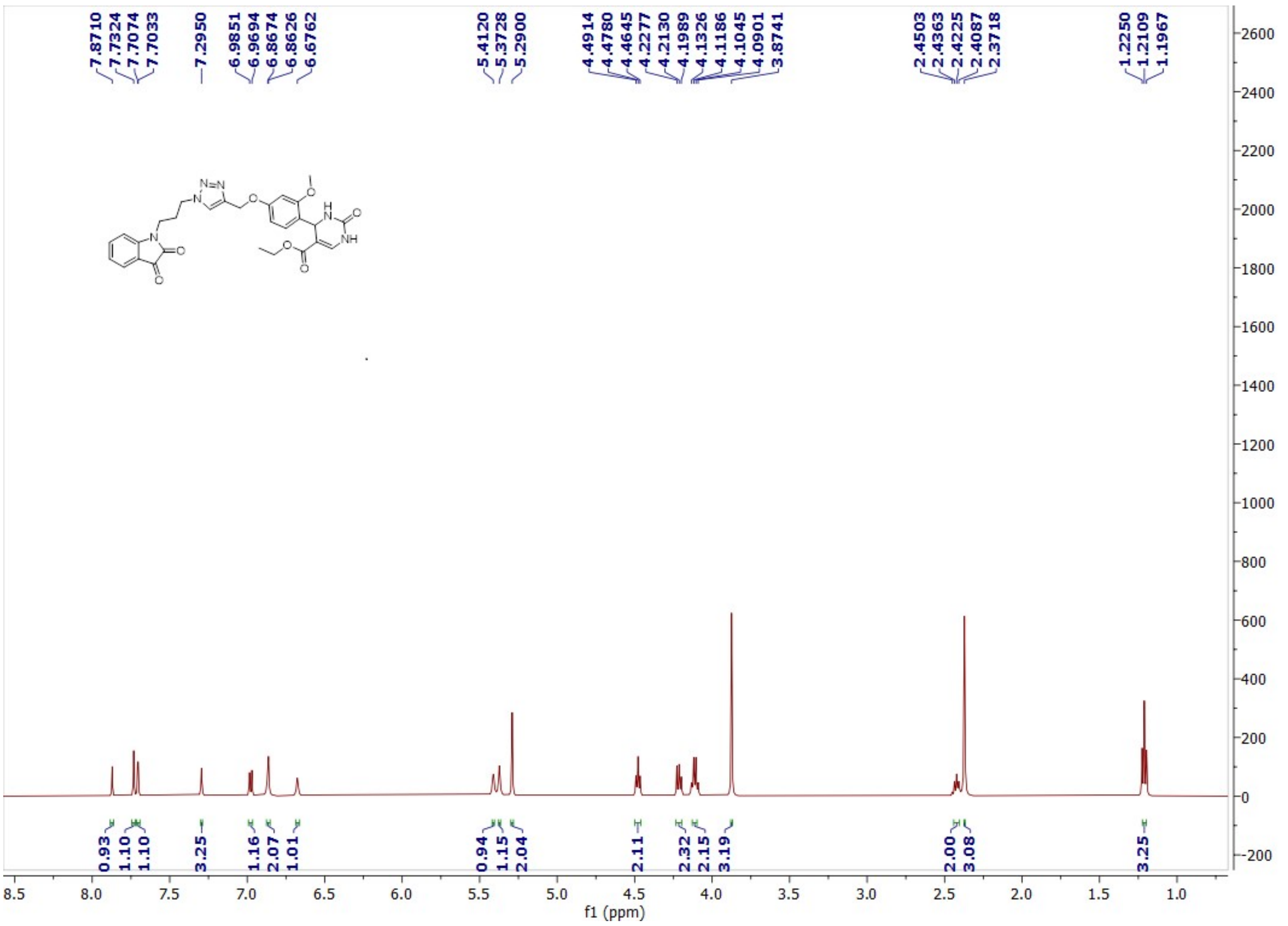

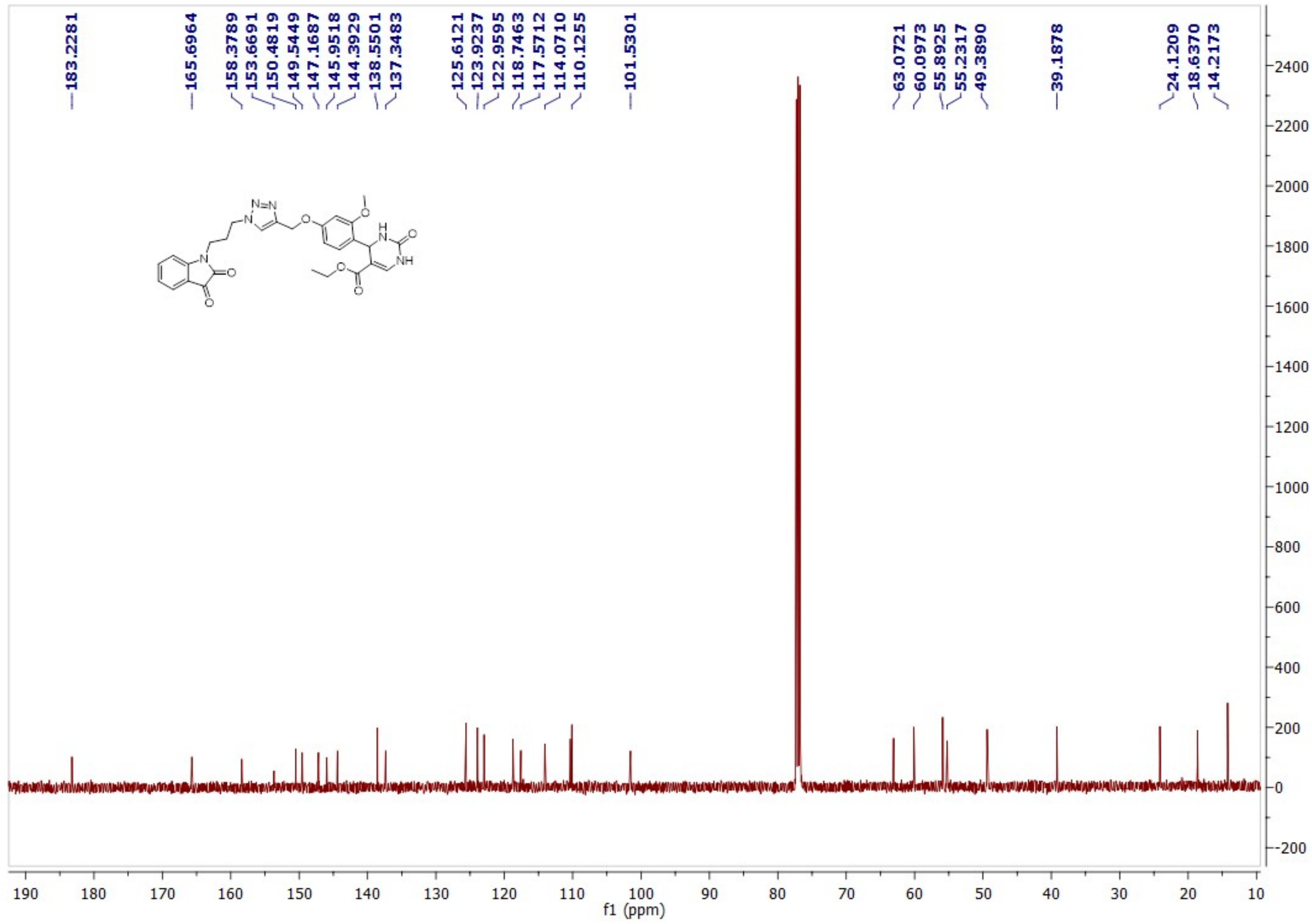

Ethyl 4-(4-((1-(4-(2,3-dioxoindolin-1-yl)butyl)-1H-1,2,3-triazol-4-yl)methoxy)-3-methoxyphenyl)-6-methyl-2-oxo-1,2,3,4-tetrahydropyrimidine-5-carboxylate (7o):

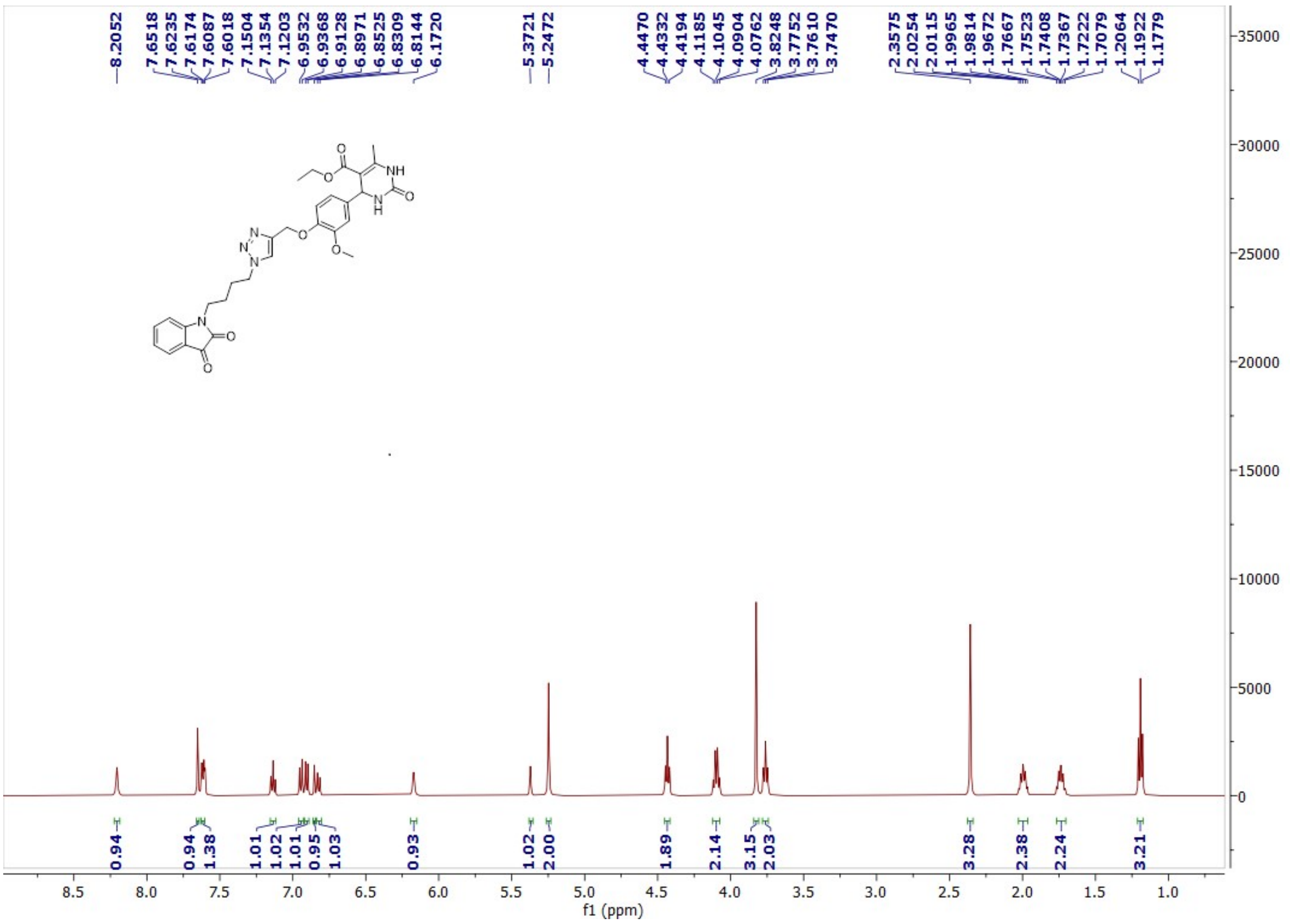

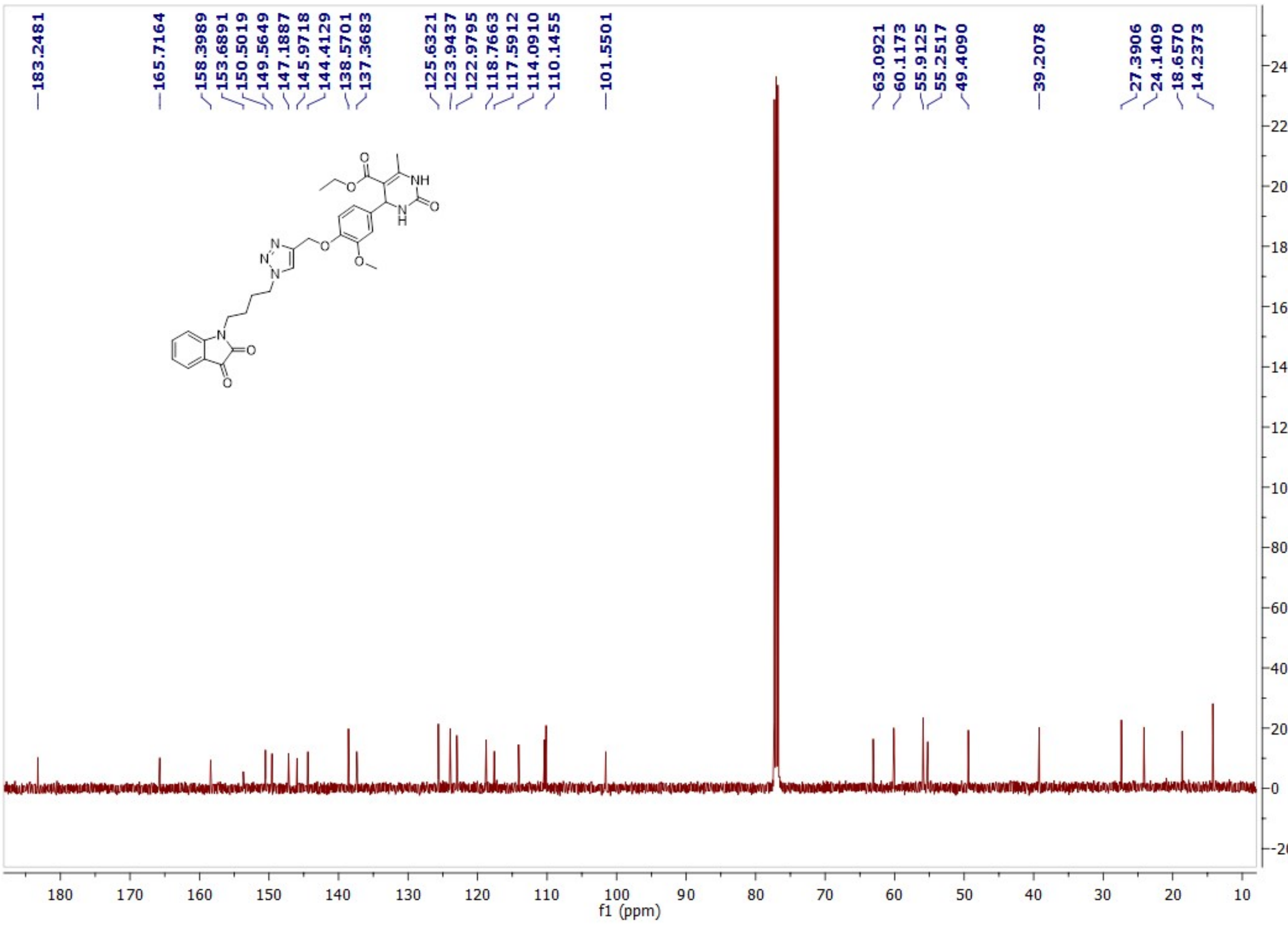

Ethyl 4-(4-((1-(2-(5,7-dibromo-2,3-dioxoindolin-1-yl)ethyl)-1H-1,2,3-triazol-4-yl)methoxy)-3-(l1-oxidanyl)phenyl)-6-methyl-2-oxo-1,2,3,4-tetrahydropyrimidine-5-carboxylate (7p):

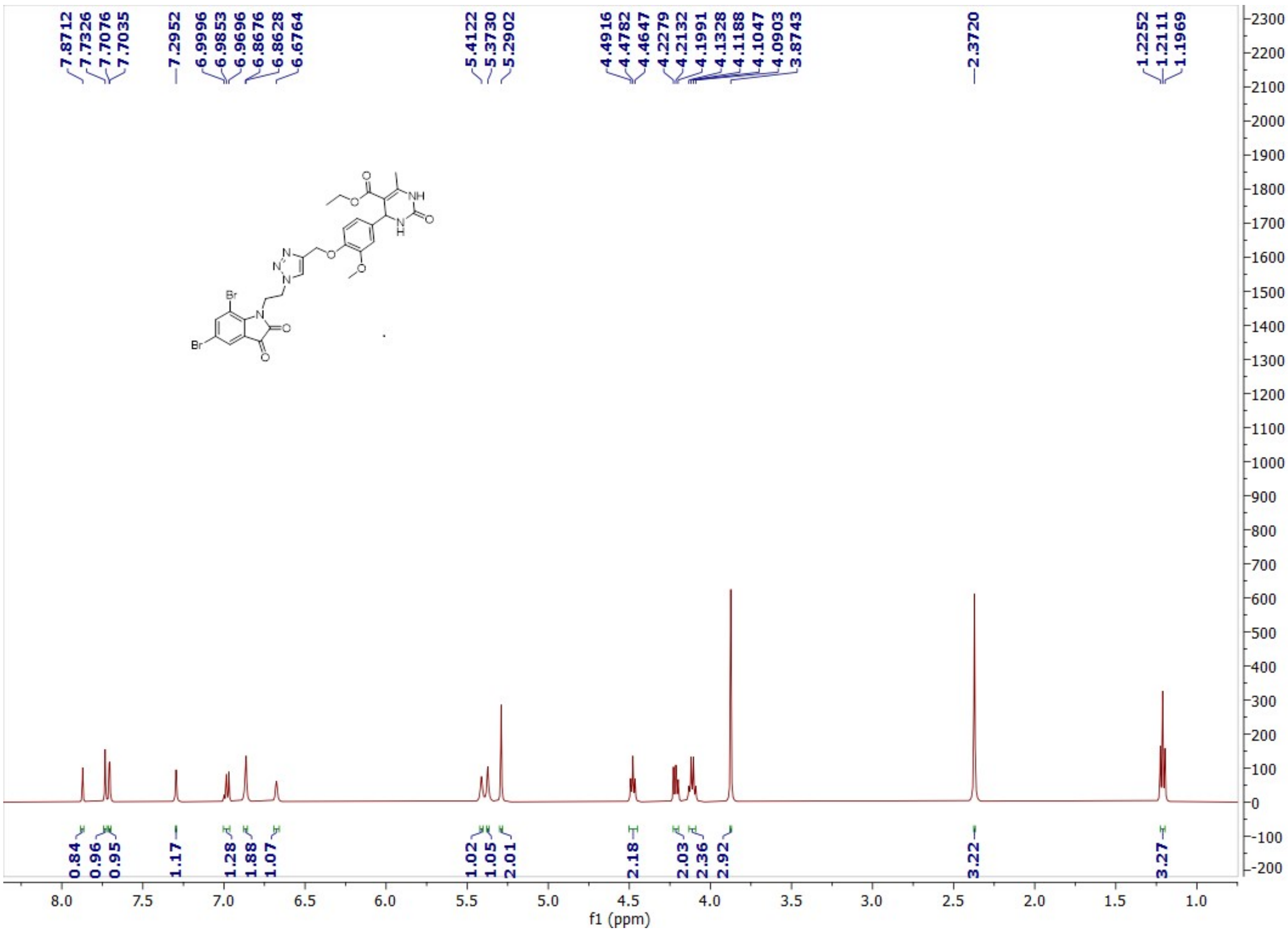

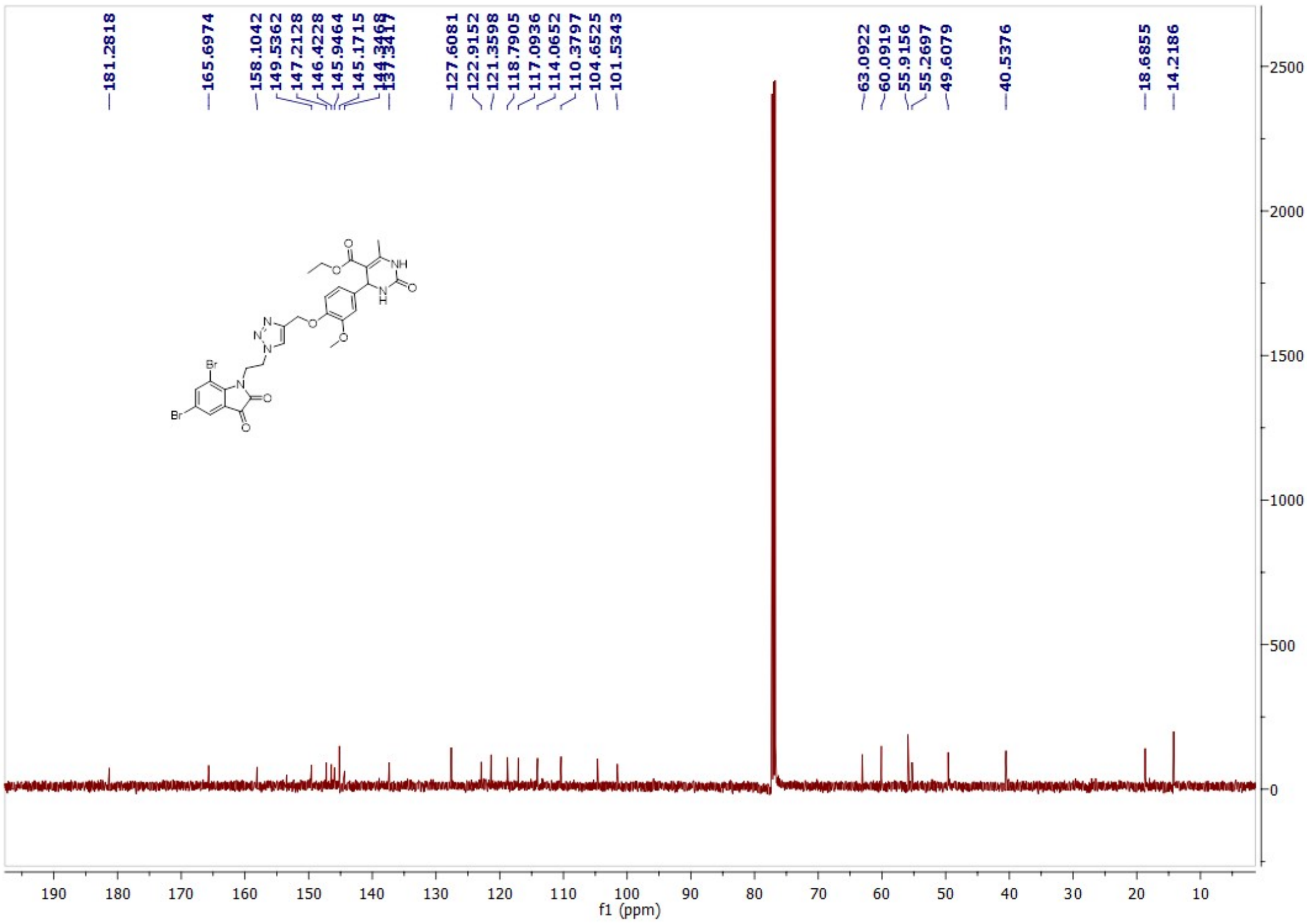

Ethyl 4-(4-((1-(3-(5,7-dibromo-2,3-dioxoindolin-1-yl)propyl)-1H-1,2,3-triazol-4-yl)methoxy)-3-methoxyphenyl)-6-methyl-2-oxo-1,2,3,4-tetrahydropyrimidine-5-carboxylate (7q):

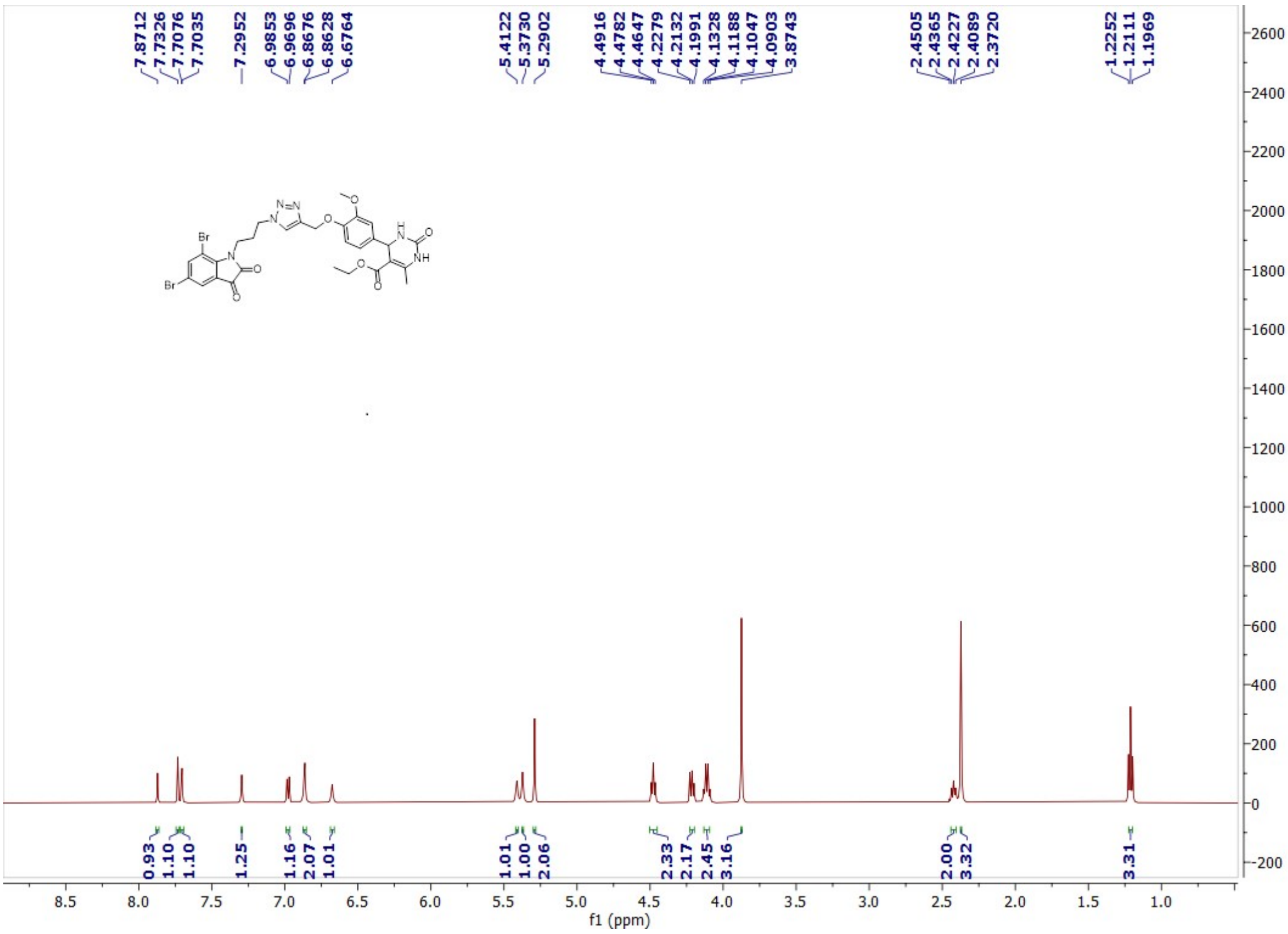

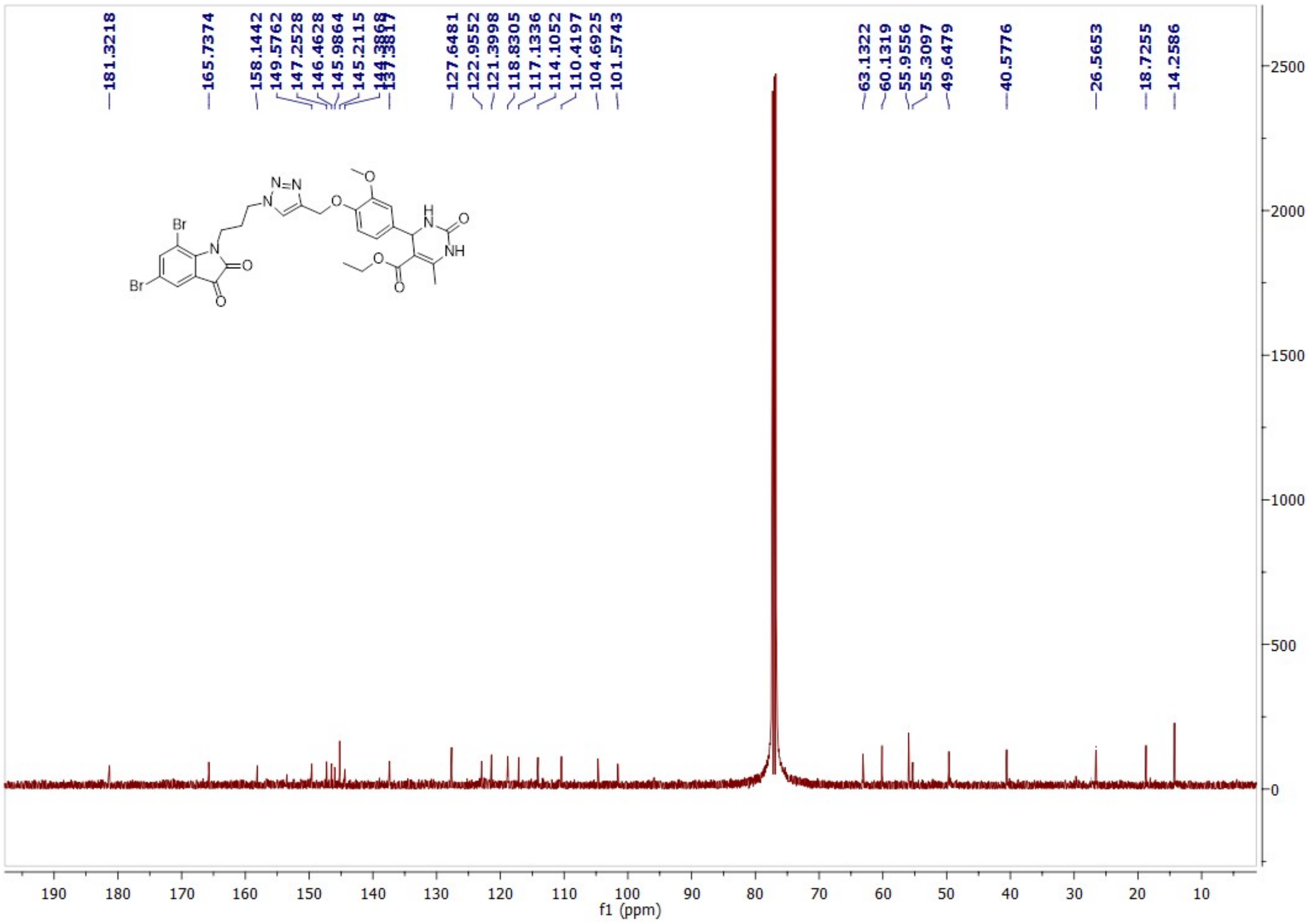

Ethyl 4-(4-((1-(4-(5,7-dibromo-2,3-dioxoindolin-1-yl)butyl)-1H-1,2,3-triazol-4-yl)methoxy)-3-methoxyphenyl)-6-methyl-2-oxo-1,2,3,4-tetrahydropyrimidine-5-carboxylate (7r):

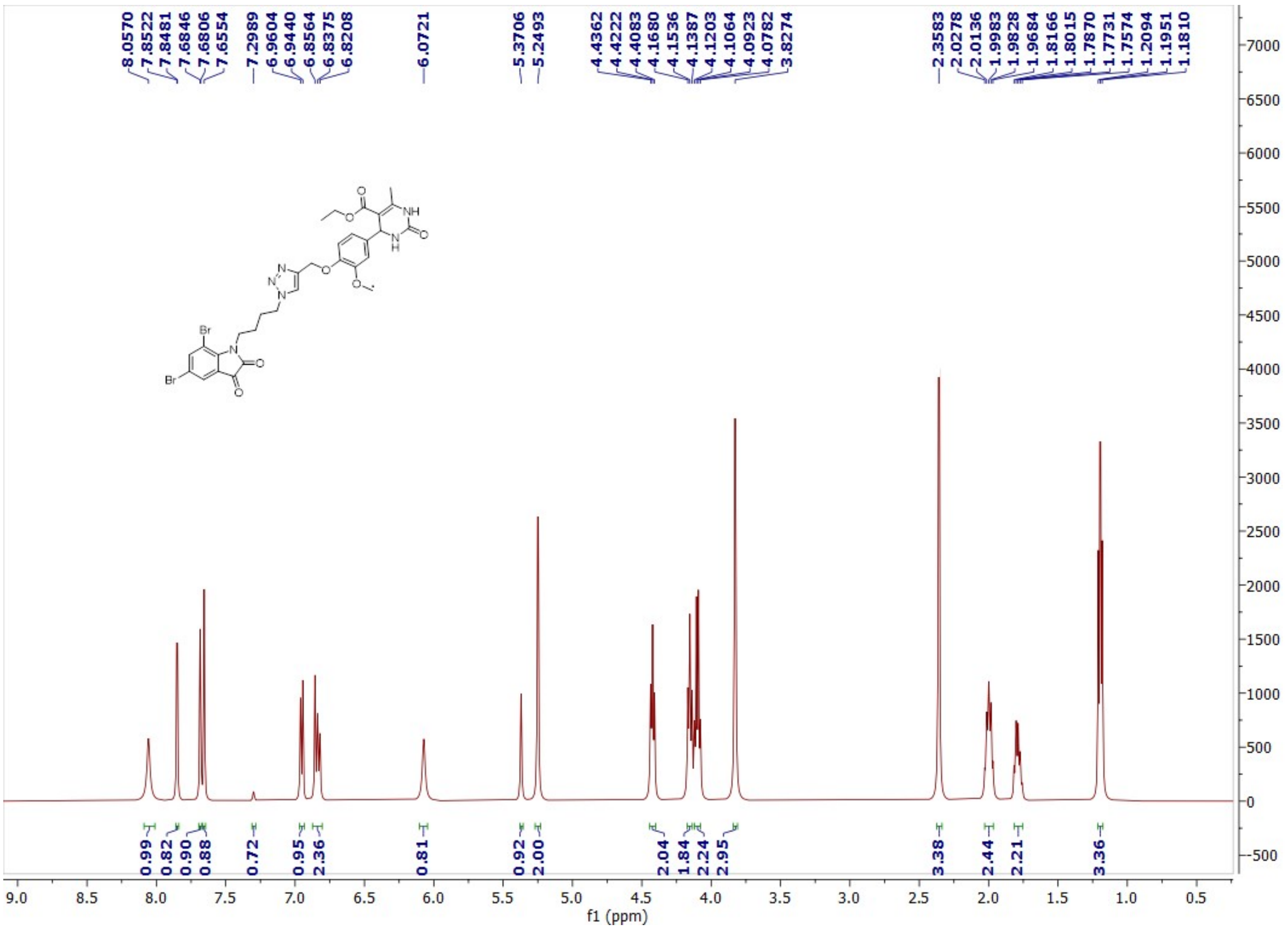

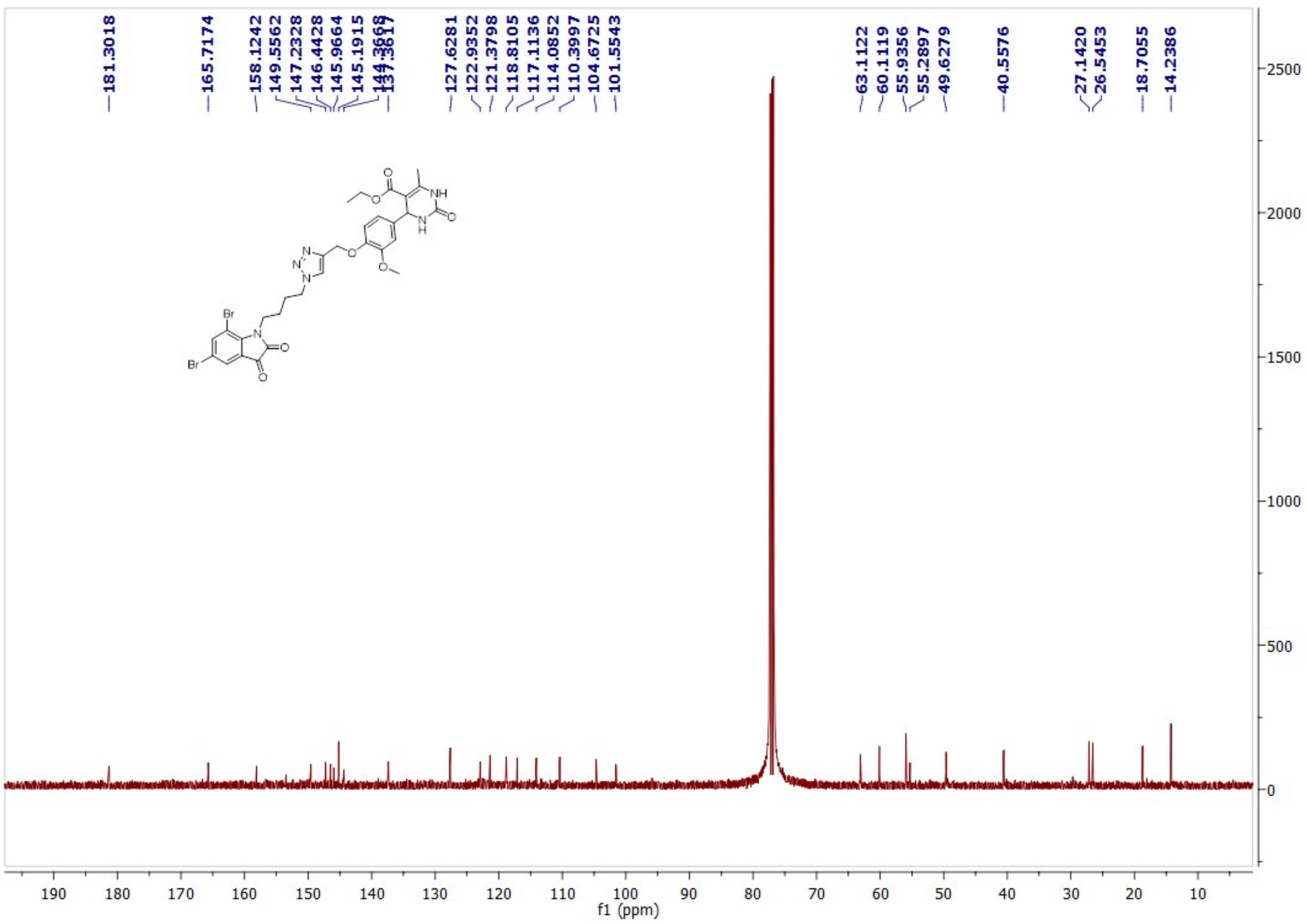

4. References

1. T. Al-Warhi, M. F. Abo-Ashour, H. Almahli, O. J. Alotaibi, M. M. Al-Sanea, G. H. Al-Ansary and H. A. Abdel-Aziz, *J. Enzyme Inhib. Med. Chem.*, 2020, **35**, 1300–1309.
2. J. R. Greenwood, D. Calkins, A. P. Sullivan and J. C. Shelley, J. Comput.-Aided Mol. Des., 2010, 24, 591–604.
3. J. C. Shelley, A. Cholleti, L. L. Frye, J. R. Greenwood, M. R. Timlin and M. Uchimaya, J. Comput.-Aided Mol. Des., 2007, 21, 681–691.
4. D. Shivakumar, J. Williams, Y. Wu, W. Damm, J. Shelley and W. Sherman, J. Chem. Theory Comput., 2010, 6, 1509–1519.
5. W. L. Jorgensen, D. S. Maxwell and J. Tirado-Rives, J. Am. Chem. Soc., 1996, 118, 11225–11236.
6. W. L. Jorgensen and J. Tirado-Rives, J. Am. Chem. Soc., 1988, 110, 1657–1666.
